# Functional and structural basis of Omicron BA.3.2.1 spike

**DOI:** 10.1016/j.celrep.2026.117812

**Published:** 2026-08-08

**Authors:** Yan Wang, Yanping Hu, Zhenhang Chen, Jing Zou, Ke Zhang, Ping Ren, Pei-Yong Shi, Bo Liang, Xuping Xie

**Affiliations:** 1 Department of Microbiology and Immunology, University of Texas Medical Branch, Galveston, TX, USA; 2 Department of Biochemistry, Emory University School of Medicine, Atlanta, GA, USA; 3 Department of Pathology, University of Texas Medical Branch, Galveston, TX, USA; 4 Department of Biochemistry and Molecular Biology, University of Texas Medical Branch, Galveston, TX, USA; 5 Sealy Institute for Drug Discovery, University of Texas Medical Branch, Galveston, TX, USA; 6 These authors contributed equally; 7 Senior author; 8 Lead contact

## Abstract

SARS-CoV-2 BA.3.2 sublineages, derived from BA.3 and carrying substantial spike divergence, raised concerns about altered fitness and antigenicity. Using BA.3.2.1 as a representative strain, we engineered live-attenuated SARS-CoV-2 encoding BA.3.2.1, LP.8.1, or XEC spikes and benchmarked them against BA.3 and KP.3. BA.3.2.1 outcompetes BA.3 in primary human airway epithelium but replicates less efficiently than JN.1 descendants and shows the greatest resistance to neutralization by KP.2/KP.3 convalescent sera. Although BA.3.2.1 RBD binds hACE2 with high affinity, its trimeric spike engages hACE2 less efficiently than LP.8.1. Cryoelectron microscopy structures reveal that BA.3.2.1 spike predominantly adopts a compact, asymmetric closed conformation stabilized by protomer rearrangements, N-linked glycosylation, and a distinct fusion-peptide-proximal region. This architecture increases spike stability, limits receptor engagement, reduces fusogenicity, and masks antibody-sensitive epitopes. Thus, BA.3.2.1 enhances immune evasion at the cost of replication fitness, providing a structural-functional explanation for BA.3.2’s limited prevalence and underscoring the need for continued variant surveillance.

## In brief

Wang et al. show that SARS-CoV-2 BA.3.2.1 escapes KP.2/KP.3 infection sera but replicates less efficiently than JN.1-lineage variants. Structural and functional analyses indicate that altered glycosylation and unique spike architecture confer immune escape while stabilizing a closed, asymmetric conformation that limits receptor engagement and viral fitness.

## Graphical Abstract

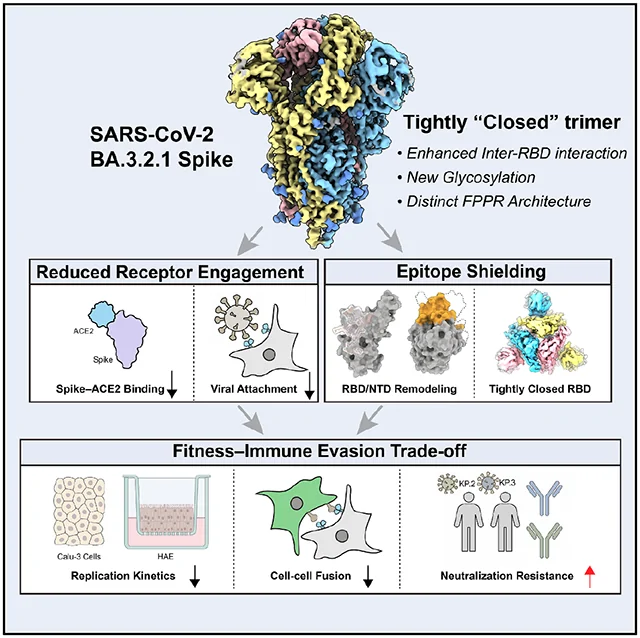

## INTRODUCTION

Severe acute respiratory syndrome coronavirus 2 (SARS-CoV-2), the etiologic agent of the coronavirus disease 2019 (COVID-19) pandemic, has caused substantial global morbidity and mortality.^1,2^ Continued antigenic drift and episodic saltation have generated variants with altered transmissibility and immune escape, most notably within the Omicron variants.^3^ The initial Omicron BA.1 marked a major evolutionary leap, characterized by extensive mutations and broad immune escape.^4-7^ Subsequent Omicron sublineages, such as BA.2, BA.3, BA.4/BA.5, and XBB, drove additional waves of infections.^8-11^ Since mid-2023, the highly divergent BA.2.86 lineage and its descendants, including JN.1, KP.2/KP.3, LP.8.1, NB.1.8.1, and XFG, have subsequently predominated globally and reshaped the SARS-CoV-2 evolution.^12-16^ In parallel, BA.3.2 emerged as a long-branch descendant of the rarely circulating BA.3. It was first detected in South Africa in November 2024 and later designated by WHO as a variant under monitoring.^17^ BA.3.2 has been detected across multiple continents with overall low prevalence compared to co-circulating JN.1 descendants.^18^

SARS-CoV-2 is an enveloped, positive-sense single-stranded RNA virus. SARS-CoV-2 entry is mediated by the trimeric spike glycoprotein, which binds angiotensin-converting enzyme 2 (ACE2) and undergoes protease-triggered conformational transitions.^19,20^ Each spike protomer contains S1 and S2 subunits separated by a furin cleavage site. S1 contains the N-terminal domain (NTD), receptor-binding domain (RBD), and subdomains SD1 and SD2. RBD often toggles between “down” and “up” conformations that regulate receptor accessibility.^21-23^ S2 includes the S2′ site (cleaved by TMPRSS2 or other host serine proteases), fusion peptide (FP), fusion-peptide proximal region (FPPR), heptad repeats (HR1 and HR2), central helix (CH), connector domain (CD), transmembrane domain (TM), and cytoplasmic tail (CT), which together mediate membrane fusion.^24^

Spike is the primary target of vaccines and therapeutic neutralizing antibodies (nAbs). RBD-directed nAbs are commonly grouped into several classes.^25^ Class I overlaps the ACE2-binding sites and engages only RBD-up.^26^ Class II competes with ACE2 but can bind RBD-up and RBD-down.^25^ Class III targets conserved outer RBD surfaces.^27-29^ Class IV recognizes cryptic epitopes exposed during RBD opening.^30,31^ Class V maps to additional cryptic epitopes opposite the ACE2 interface.^32^ NTD- and S2-directed nAbs provide complementary neutralization mechanisms.^33-36^ Thus, spike mutations can affect viral fitness and immune escape by altering receptor binding, conformational dynamics, proteolytic activation, fusion activity, and antibody epitope exposure.

BA.3.2 has drawn substantial attention because of its extensive spike divergence.^12,37^ Prior studies suggest that BA.3.2 exhibits pronounced immune evasion but reduced infectivity, fusogenicity, and replication compared with JN.1-descendant variants.^12,18,37,38^ However, many analyses have relied on pseudovirus systems, with limited evaluation using authentic virus in physiologically relevant primary human airway epithelium (HAE). A rigorous functional-structural definition of BA.3.2 benchmarked against contemporaneous variants is therefore needed to define how its extensive mutations alter spike structure, replication fitness, and antigenicity, which is critical for understanding its evolutionary potential.

Here, we investigated BA.3.2.1 as a representative BA.3.2 subvariant. We engineered attenuated mNeonGreen (mNG) reporter SARS-CoV-2s encoding BA.3.2.1, LP.8.1, or XEC spikes and benchmarked them against BA.3 and KP.3 in Calu-3 cells and HAE. We assessed spike processing, replication, competition fitness, cell-cell fusion, virus-cell attachment, hACE2 binding, and neutralization by KP.2/KP.3 convalescent sera. We further determined cryoelectron microscopy (cryo-EM) structures of BA.3.2.1 and LP.8.1 spikes in apo and hACE2-bound states. Our findings reveal that BA.3.2.1 achieves broad immune escape through extensive antigenic remodeling and a compact closed spike architecture, but at the cost of reduced receptor accessibility and replication fitness, providing a structural-functional explanation for BA.3.2’s limited expansion.

## RESULTS

### Generation and characterization of BA.3.2.1-spike mNG SARS-CoV-2

**Figure 1.**
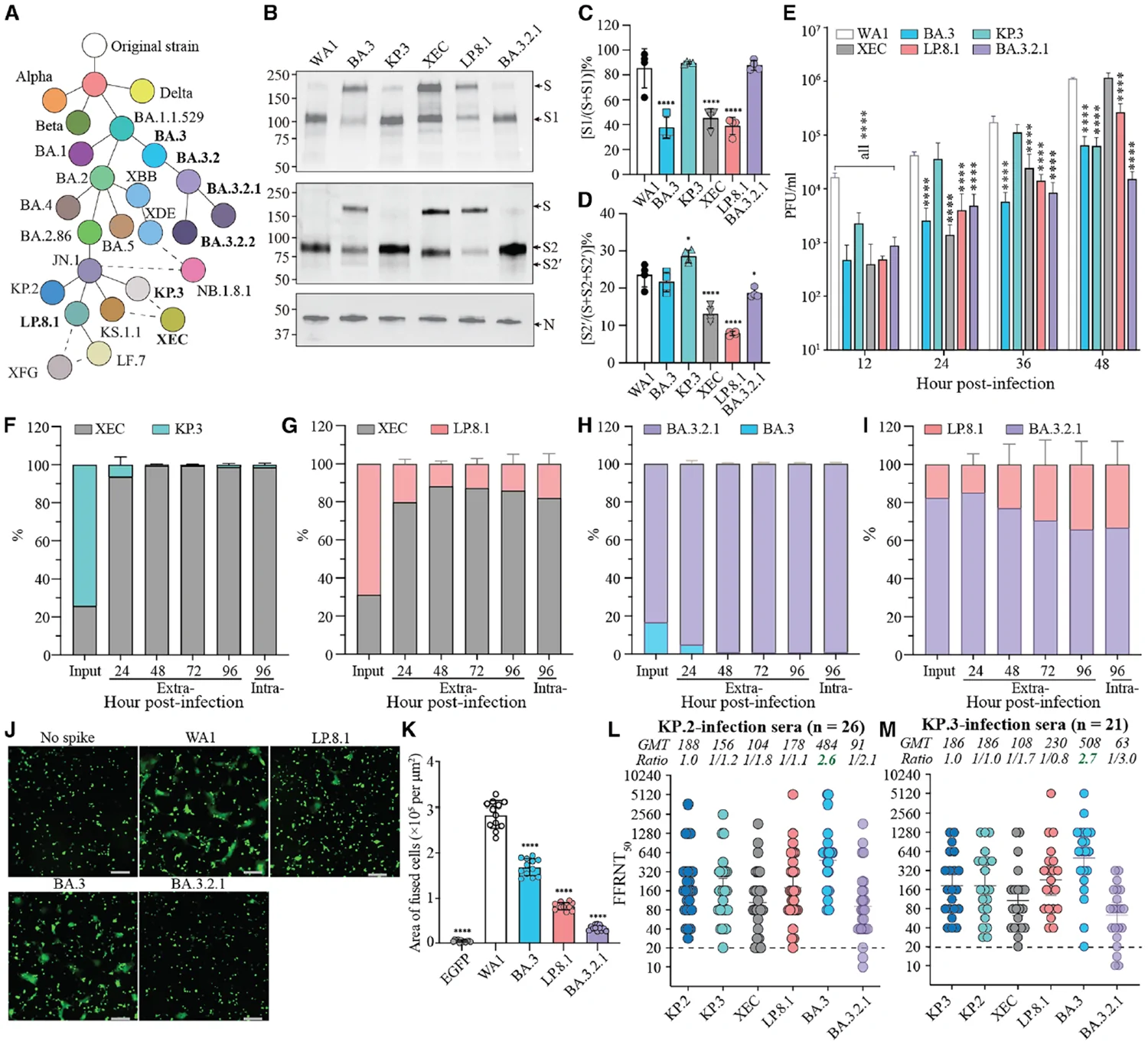
Characterization of mNG SARS-CoV-2 spike variants (A) Phylogenetic relationship of variants, adapted from CDC data (www.cdc.gov/covid/php/variants/variants-and-genomic-surveillance.html). The dashed line indicates variant recombination. (B) Western blot analysis of spike and nucleocapsid in virions using anti-S1 (top), anti-S2 (middle), and anti-N (bottom) antibodies. (C and D) Ratio of S1 to total spike (S plus S1; C) and S2′ to total spike (S plus S2 and S2′ ; D) in virions. Data shown (mean ± SD) are representative of four independent experiments. *p < 0.05; ****p < 0.0001, one-way ANOVA with Dunnett’s multiple-comparison corrections. (E) Growth kinetics of mNG SARS-CoV-2 spike variants in Calu-3 cells. Data shown (mean ± SD) are representative of four independent experiments. ****p < 0.0001, two-way ANOVA with Dunnett’s multiple-comparison corrections (compared with WA1). (F–I) Competition analysis of spike variants in HAE. The percentages of XEC- and KP.3-spike RNA (F), XEC- and LP.8.1-spike RNA (G), BA.3.2.1- and BA.3-spike RNA (H), LP.8.1- and BA.3.2.1-spike RNA (I) are shown. Data shown (mean ± SD) are representative of five independent experiments. (J) Representative images of cell-to-cell fusion. Scale bars, 200 μm. (K) Quantification of fusion. Data shown (mean ± SD) are representative of twelve independent experiments. ****p < 0.0001, one-way ANOVA with Dunnett’s multiple-comparisons corrections (compared with WA1). (L and M) FFRNT_50_ of KP.2- (L) or KP.3-infection (M) sera against spike variants. Solid lines and values indicate GMTs. Error bars show 95% confidence intervals. GMT ratios relative to KP.2- or KP.3-spike are shown. Dotted lines indicate the FFRNT limit of detection. *p* values (Wilcoxon matched-pairs signed-rank test) are provided in Table S3.

BA.3.2 emerged in late 2024 while JN.1-descendant variants co-circulated in a successive epidemiological pattern: KP.3→XEC→LP.8.1 (Figure 1A). By the completion of this study, XFG and NB.1.8.1 had become dominant. Two BA.3.2 subline-ages were reported: BA.3.2.1, containing P681R and P1162R, and BA.3.2.2, carrying K356T, A575S, and P681H.^18^ We focused on BA.3.2.1 because it emerged earlier. To characterize BA.3.2 relative to JN.1-descendant spikes, we engineered BA.3.2.1, XEC, and LP.8.1 spike genes into a reporter SARS-CoV-2 derived from USA-WA1/2020 (WA1), in which ORF7 is replaced by mNG (Figures S1A and S1B). This reporter virus is highly attenuated *in vivo* and suitable for BSL-3 variant studies.^13,39,40^ Recombinant viruses were recovered in Vero E6-TMPRSS2 cells, with P1 titers exceeding 10^6^ plaque-forming unit (PFU)/mL. Previously described BA.3- and KP.3-spike mNG SARS-CoV-2s were also included for comparison.^4,40^ All Omicron spike variants formed plaques smaller than WA1 but similar to each other (Figure S2A).

Western blot analysis of virions released from Vero E6 cells revealed distinct S cleavage patterns. KP.3 and BA.3.2.1 showed efficient S1/S2 cleavage (>80%), comparable to WA1, whereas BA.3, XEC, and LP.8.1 showed lower cleavage (~35%–45%) (Figures 1B, 1C, and S2C). The S2′ band was more abundant in WA1, BA.3, and KP.3 (22%–30%), intermediate in BA.3.2.1 (18%), and lowest in XEC and LP.8.1, with LP.8.1 below 10% (Figures 1B and 1D). Total spike-to-N ratios were similar across variants, except for LP.8.1, which was reduced to ~70% of WA1 levels (Figures S2C and S2D).

We next compared viral replication in Calu-3 cells (Figure 1E). As expected, all Omicron spike variants replicated less efficiently than WA1. Among JN.1 descendants, replication ranked KP.3 > XEC > LP.8.1, contrasting with their epidemiological succession. BA.3- and BA.3.2.1-spike viruses replicated below the JN.1 descendants, with BA.3.2.1 being the most attenuated.

### BA.3.2.1 spike is more attenuated than JN.1 descendants in HAE

We also compared viral replication in HAE culture using a paired competition assay, a well-established model for assessing SARS-CoV-2 replication fitness.^4,40-42^ Four variant pairs were tested based on evolutionary and epidemiological relevance: KP.3 versus XEC, XEC versus LP.8.1, BA.3 versus BA.3.2.1, and LP.8.1 versus BA.3.2.1. Although viruses were mixed at equal PFU, input RNA ratios varied among pairs, indicating differences in specific infectivity (Figures S2E-S2H).

Competition assays revealed marked fitness differences (Figures 1F-1I and S2E-S2H). Among JN.1 descendants, XEC-spike rapidly outcompeted KP.3-spike, increasing from 25% at input to >90% by 24–96 h post-infection (p.i.). XEC-spike also dominated LP.8.1-spike, rising from 30% to >80% over 24–96 h p.i. BA.3.2.1-spike strongly outgrew BA.3-spike but was less fit than LP.8.1-spike, with its relative abundance declining from 24 to 96 h p.i.

### BA.3.2.1 spike exhibits reduced cell-cell fusion activity

We assessed spike-mediated membrane fusion using a cell-cell fusion assay.^43^ 293T cells co-transfected with spike and eGFP were co-cultured with 293T-ACE2 cells, and fusion was quantified. WA1 spikes showed the strongest fusion activity. Relative to WA1, fusion activity was reduced to 59% for BA.3, 29% for LP.8.1, and 12% for BA.3.2.1 (Figures 1J and 1K), indicating that the BA.3.2.1 spike is less fusogenic than BA.3 and LP.8.1.

### BA.3.2.1 spike exhibits the greatest immune escape among tested variants

We evaluated the neutralization sensitivity using convalescent sera from 47 adults with documented KP.2 or KP.3 infection (Tables S1; S2) by a fluorescent focus reduction neutralization test (FFRNT).^40^ KP.2-infection sera neutralized KP.2-, KP.3-, XEC-, LP.8.1-, BA.3-, and BA.3.2.1-spike mNG SARS-CoV-2 with geometric mean titers (GMT) of 188, 156, 104, 178, 484, and 91 (Figure 1L), respectively. KP.3-infection sera showed GMTs of 186, 186, 108, 230, 508, and 63 against the same variants (Figure 1M). KP.2- and KP.3-spike exhibited similar neutralization sensitivity, consistent with previous findings.^40^ BA.3-spike was most sensitive, whereas BA.3.2.1-spike showed the strongest resistance. XEC-spike displayed modest resistance, while LP.8.1-spike remained comparable to KP.2-/KP.3-spike. These results indicate BA.3.2.1 has the greatest escape from neutralization elicited by prior KP.2 or KP.3 infection.

### BA.3.2.1 spike exhibits less structural flexibility than the LP.8.1 spike

**Figure 2.**
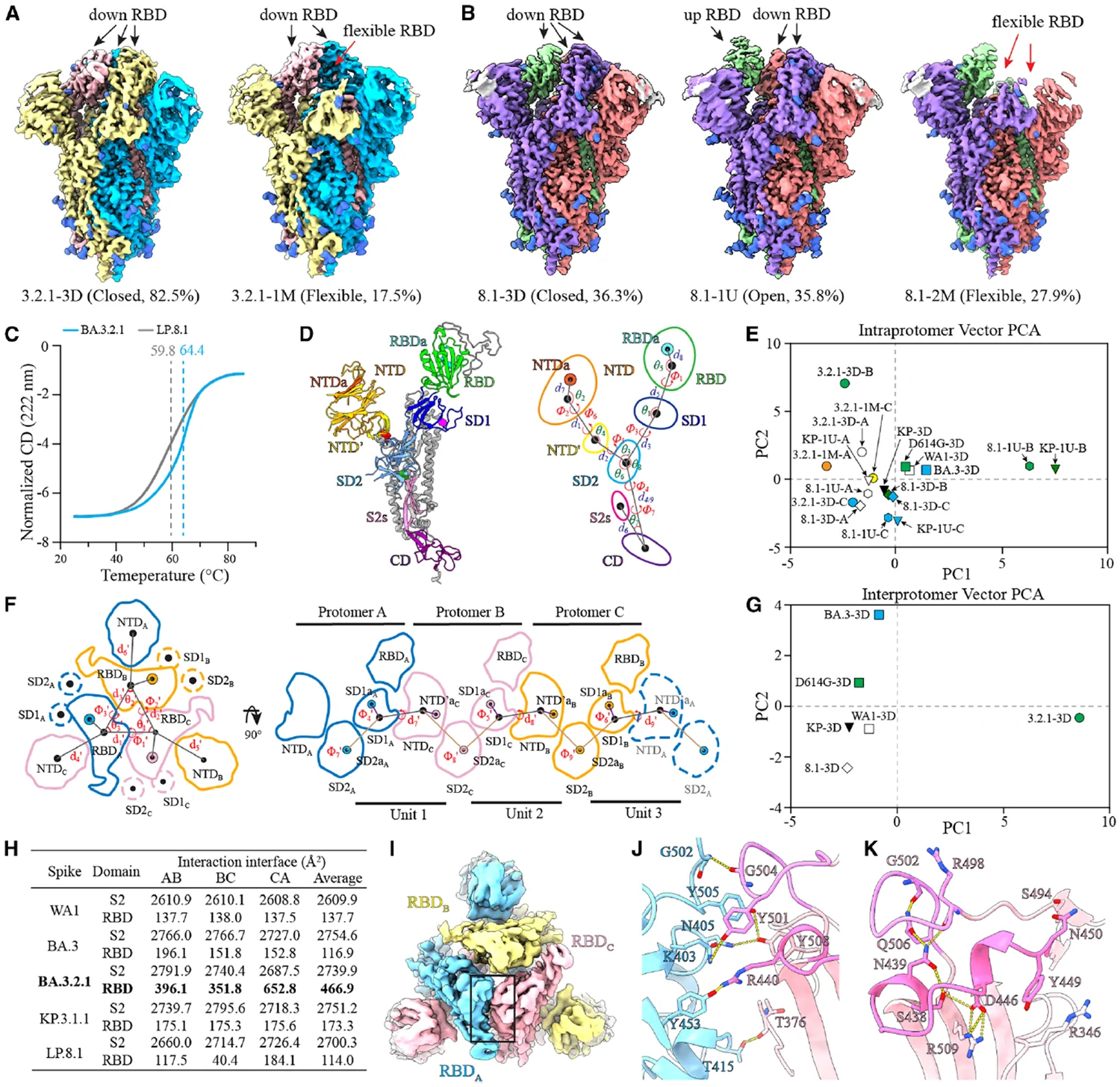
Structures of BA.3.2.1 and LP.8.1 spikes (A) Cryo-EM maps of BA.3.2.1 spike in closed and flexible states, with protomers colored in light blue, yellow, and light pink. (B) Cryo-EM maps of LP.8.1 spike in closed, open, and flexible states, with protomers colored in light magenta, green, and salmon. (C) CD melting curves of BA.3.2.1 and LP.8.1 spikes. (D) Intraprotomer vector analysis. Left: atomic model of protomer A from closed BA.3.2.1 spike highlighting vector positions. Right: schematic annotating vectors, angles, and dihedrals. (E) PCA of the intraprotomer vectors. (F) Interprotomer vector analysis. Left, top view of S1 domains. Right, schematic of interprotomer vectors, angles, and dihedrals. (G) PCA of the interprotomer vectors. (H) Interface areas between neighboring S2 subunits and RBDs in closed spike conformations. (I) Top view of the closed BA.3.2.1 spike cryo-EM map. The black box highlights the RBD_A_/RBD_C_ interface. (J) Interaction networks between RBDA and RBDC. The 497–505 and 438–451 loops are colored plum and violet, respectively. Dashed lines indicate hydrogen bonds. (K) Intramolecular interactions within RBDC that stabilize the remodeled 497–505 and 438–451 loops. Dashed lines indicate hydrogen bonds and salt bridges.

To investigate mechanisms underlying BA.3.2.1 spike function, we determined its trimeric structure by single-particle cryo-EM (Figures S3A-S3C; Table S4). 82.5% BA.3.2.1 spike particles adopted a closed 3-RBD-down conformation (3.2.1-3D), while 17.5% displayed a flexible state (3.2.1-1M) with two down RBDs and one unresolved RBD (Figure 2A). These structures were resolved at 2.6 Å and 3.0 Å, respectively (Figures S3D and S3E). For comparison, we determined the cryo-EM structure of LP.8.1 spike (Figures S4A-S4C; Table S5). Similar to JN.1.11 and KP.3.1.1,^44^ LP.8.1 spike displayed three conformational states: closed (8.1-3D, 36.3%), open (1-RBD-up, 8.1-1U, 35.8%), and flexible (2-RBD-unresolved, 8.1-2M, 27.9%), resolved at 2.5–2.6 Å (Figures 2B and S4D-S4F).

3D variability analysis (3DVA) further showed limited conformational heterogeneity in the closed BA.3.2.1 spike, mainly localized to HR2 with minor NTD and RBD movement (Figures S5A and S5B; Video S1). Its flexible state overall resembled the closed conformation (root-mean-square deviation [RMSD] = 0.40 Å) (Figure S5C), except for one unresolved RBD with greater mobility (Figures S5D and S5E; Video S2). In contrast, LP.8.1 displayed broader and higher-amplitude variability across the NTDs and RBDs in closed, open, and flexible states (Figures S5F-S5K; Videos S3, S4, and S5). Consistently, circular dichroism (CD) analysis revealed that BA.3.2.1 spike was more thermostable, with a melting temperature 4.6°C higher than LP.8.1 (Figure 2C). Together, these findings demonstrate that the prefusion BA.3.2.1 spike is more conformationally constrained than LP.8.1.

### Quaternary structure of BA.3.2.1 spike

Structural alignment revealed greater variations among BA.3.2.1 RBDs and SD1s than LP.8.1 (Figures S5L and S5M). To quantify these differences, we performed vector-based quaternary analysis as described previously (Figure 2D).^45,46^ We also included WA1, BA.3, D614G, and KP.3.1.1 spike structures for comparison.

Principal component analysis (PCA) of 25 intra-protomer descriptors separated RBD-down and RBD-up conformations (Figure 2E; Table S6). BA.3.2.1-Closed (3.2-3D) localized within the RBD-down cluster but occupied a distinct position, reflecting compact hinge geometry and altered SD1-SD2 spacing. It also showed marked protomer asymmetry along PC2, with protomer B displaying the largest auxiliary shift. By contrast, LP.8.1-Open (8.1-1U-B) and KP.3.1.1-Open (KP-1U-B) populated the RBD-up cluster, driven by increased hinge openness. LP.8.1-Closed remained compact but differed from BA.3.2.1 in its auxiliary configuration, whereas WA1, D614G, and BA.3 clustered near the origin.

Descriptor-level analysis confirmed asymmetric protomer organization in closed BA.3.2.1 (Figures S6A-S6C). Protomer C showed a more down-oriented and twisted RBD-SD2/NTD′ relationship (increased θ_1_/θ_5_ and lower Φ_1_/Φ_3_), whereas protomer B exhibited outward SD1 rotation (reduced θ_3_/θ_8_, with corresponding Φ_5_/Φ_7_/d_3_ shifts) and a modest RBD elevation (lower θ_1_, increased d_3_/d_5_, and decreased d_9_). These placements supported a compact intra-protomer hinge and a distinct SD1–SD2 configuration in BA.3.2.1-Closed spike.

We next performed inter-protomer vector analysis of closed spike trimers using SD1/RBD-NTD/NTD′ conformational units relative to SD2 (Figure 2F).^45,46^ PCA of cross-protomer descriptors resolved variant-specific trimer arrangements (Figure 2G; Table S7). BA.3.2.1-Closed occupied the PC1-positive extreme, indicating a compact quaternary architecture with contracted inter-RBD spacing and constrained RBD-centric torsions. By contrast, BA.3 lay at the PC2-positive extreme, consistent with pronounced SD1/SD2-NTD′ rearrangements; LP.8.1 was both PC1- and PC2 negative, reflecting expanded inter-protomer RBD spacing with comparatively reduced orthogonal torsion. WA1, D614G, and KP.3.1.1 occupied intermediate positions.

Consistently, inter-protomer descriptors showed that BA.3.2.1 had a contracted inter-RBD triad (reduced d_1_′-d_3_′/Φ_1_′ and increased Φ_2_′/Φ_9_′), tightened RBD angular arrangement (decreased θ_3_′/θ_1_′ and increased θ_2_’), and pronounced cross-protomer SD1-NTD′ rearrangements (increased d_7_′, decreased d_8_′-d_9_′, and increased Φ_6_′) (Figures S6D-S6F). Together, these analyses identify BA.3.2.1-closed spike as a compact, “locked” quaternary architecture with contracted inter-RBD geometry, compact intra-protomer hinging, and a distinct SD1/SD2 auxiliary signature.

### Unique RBD-RBD interface in closed BA.3.2.1 spike

The compacted BA.3.2.1 spike prompted closer examination of interprotomer RBD-RBD interfaces. Protein interfaces, surface, and assemblies (PISA) analysis revealed that BA.3.2.1 has a substantially larger RBD-RBD interface than other variants, including LP.8.1, while S2 contact areas remained similar (Figure 2H). The largest interface was observed between RBD_A_ and RBD_C_, supported by cryo-EM density (Figure 2I). Local refinement identified multiple contacts across this interface, including T376-T415, R440-Y453, Y501-K403, G504-G502, and Y508-N405/Y505 (Figure 2J). These interactions were accompanied by remodeling of RBDC, including bending of the 497–505 loop toward the interface and reorganization of the 438–451 segment into two short helices (438–440 and 446–448) flanking a protruding loop (441–445) that extends toward RBD_A_ (Figure S7A). The RBD_B_ 443–450 loop was also re-oriented. Additional intramolecular contacts further stabilized this region (Figure 2K). In contrast, LP.8.1 RBDs were highly similar across protomers and closely matched WA1 and KP.3 RBDs (pairwise RMSD 0.62–0.69 Å), aside from minor, Omicron-conserved changes in the 365–373 loop due to convergent mutations S371F, S373P, and S375F (Figure S7B).^47,48^ These features explain enhanced RBD packing, restricted RBD mobility, and clamping of the BA.3.2.1 spike in a tightly closed conformation.

### BA.3.2.1 NTD remodeling

Beyond the RBD, BA.3.2.1 showed substantial remodeling of the NTD, a major target of nAbs. The NTD antigenic supersite comprises five surface-exposed loops, N1-N5 (Figure S8A).^49^ To compare this region between BA.3.2.1 and LP.8.1, we performed local refinement of the NTDs (Figures S3I and S4G; Tables S8 and S9). Unresolved loops were subsequently modeled using Phenix’s predict-and-build workflow guided by AlphaFold.^50,51^

**Figure 3.**
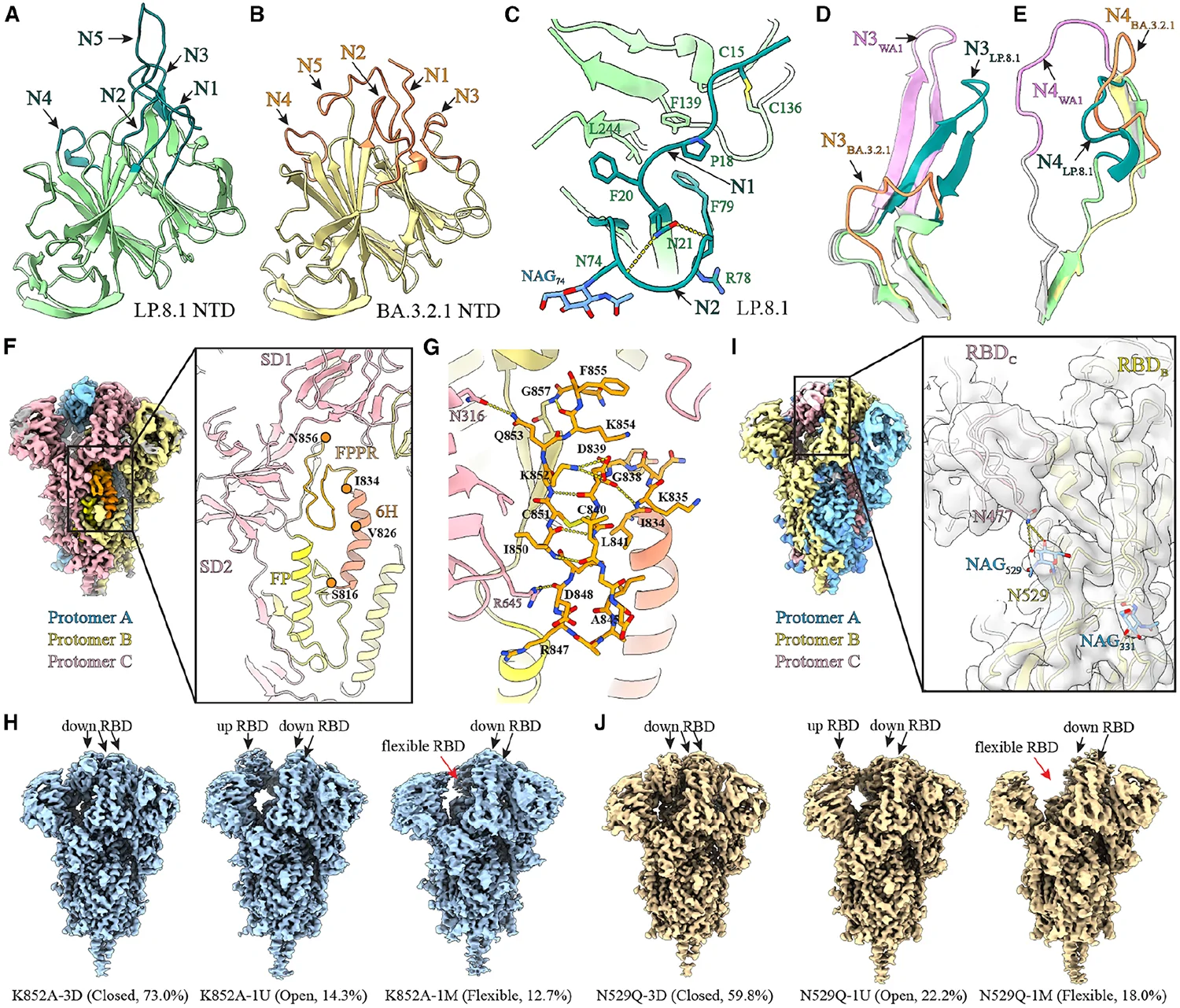
Structural analysis of BA.3.2.1 spike (A and B) AlphaFold/cryo-EM model of LP.8.1 NTD (A) and BA.3.2.1 NTD (B). LP.8.1 and BA.3.2.1 loops are colored teal and orange, respectively. (C) Interaction network stabilizing the N1/N2 thread-through arrangement in LP.8.1 NTD. (D and E) Close-up view of the N3 (D) and N4 loops (E) in WA1 (plum), BA.3.2.1 (orange), and LP.8.1 (teal) NTDs. (F) BA.3.2.1 FPPR structure. Left, side view of the closed BA.3.2.1 spike cryo-EM map with the FPPR boxed. Right, enlarged view of the boxed region. FP, FPPR, and 6H are colored yellow, orange, and salmon, respectively. (G) Interaction network stabilizing the BA.3.2.1 FPPR conformation. Dashed lines indicate hydrogen bonds or salt bridges. (H) Cryo-EM maps of BA.3.2.1 K852A spike in closed, open, and flexible states. (I) Cryo-EM density around the N529 glycan in protomer B of BA.3.2.1 spike. (J) Cryo-EM maps of BA.3.2.1 N529Q spike in closed, open, and flexible states.

Structural comparison revealed marked differences between LP.8.1 and BA.3.2.1 NTDs (Figures 3A and 3B). In LP.8.1, N1 and N2 were well resolved (Figure S8B). The 17–20 Met-Pro-Leu-Phe (MPLF) insertion, Δ24-27, and Δ69-70 deletions positioned N1 to thread through N2. This unique N1-N2 crossing was stabilized by the C15-C136 disulfide bond, hydrogen bonds involving N21, N74, and R78, and hydrophobic interactions involving F79-P18-F139 and F20-L244 (Figures 3C and S8B). This crossing has not been observed in other SARS-CoV-2 spike structures except in XBB.1.5 (Figure S8C), requiring stabilization by an NTD-directed monoclonal antibody.^52,53^ In contrast, BA.3.2.1 showed poorly resolved N1 and N2, with modeling suggesting a shorter N2 and an N1 position outside N2, akin to WA1 (Figures 3B, S8D, and S8E).

BA.3.2.1 carried a rare Δ136-147 that remodels N3, replacing its original β sheets with a shortened loop. In addition, a Δ242-243 deletion shortens and repositions N5 away from N3, further reshaping the NTD supersite (Figures 3D, S8A, and S8F). The N3 with Δ136-147, previously reported in BA.2.87.1 but structurally unresolved,^54,55^ was characterized here at high resolution (Figure 3D). Finally, S172F and K187T substitutions alter the orientation of N4 relative to WA1 (Figures 3E and S8A). Together, these features demonstrate extensive remodeling of the BA.3.2.1 NTD.

### BA.3.2.1 spike adopts a distinct FPPR conformation

We identified a distinct FPPR (residues 834–856) conformation in protomer B of the closed BA.3.2.1 spike. Unlike the canonical loop-helix-loop FPPR, this region adopted a loop-only configuration displaced from SD1, with residues D839-A845 running parallel to R847-F855 (Figures 3F and S9A). This conformation was stabilized by a C840-C851 disulfide bond, a D839-K852 salt bridge, and backbone hydrogen bonds and additional contacts with neighboring regions, including K835/G838, D848/R645, and Q853/N316 (Figure 3G). Notably, residues 826–833, which form a flexible loop in WA1 and B.1.1.529^56^(Figures S9B and S9C), adopted additional α-helical turns in BA.3.2.1, extending the canonical three-helix (3H) segment into a six-helix segment, hereafter termed 6H, spanning residues 816–833 (Figures 3F and 3G). 3DVA suggested that the 6H-loop FPPR in BA.3.2.1 protomer B was associated with reduced motion of neighboring SD1 and SD2 and stabilization of RBD_C_ in the closed state (Video S6).

In contrast, FPPR density was absent in BA.3.2.1 protomers A and C and in all LP.8.1 protomers (Figures S9A and S9D). These unresolved FPPRs likely adopt the canonical 3H-loop-helix-loop configuration (3H-LHL) previously reported for WA1 and B.1.1.519, which is associated with greater SD1/SD2 motion (Figures S9E and S9F; Video S7).

A852K is the only BA.3.2.1-specific change in the FPPR and forms the D839-K852 salt bridge (Figure 3G). To assess K852’s role, we generated a K852A revertant spike. Cryo-EM analysis showed that K852A increased conformational heterogeneity, with 73.0% closed, 14.3% one-RBD-up, and 12.7% flexible particles (Figures 3H and S10A-S10F). Although the closed K852A structure remained similar to BA.3.2.1 and retained inter-RBD contacts, FPPR density was unresolved in all protomers (Figures S10G-S10J; Tables S10 and S11). 3DVA suggested that K852A shifts the FPPR toward the 3H-LHL conformation with increased FPPR, SD1, SD2, and global spike motion (Figure S10K; Videos S8 and S9). Together, these data support an important role of K852 in stabilizing the FPPR 6H-loop architecture and the closed BA.3.2.1 spike conformation.

### N529 glycosylation stabilizes the closed BA.3.2.1 spike

Spike glycans regulate folding, conformational dynamics, receptor binding, and immune evasion.^57-59^ WA1 spike carries 22 conserved N-linked glycosylation sites. BA.3.2.1 and LP.8.1 spikes shared most core N-glycosylation sites but differed at several additional sites (Figure S11A). BA.3.2.1 has three new sites, including N99 (I101T), N185 (K187T), and N529 (K529N). In our cryo-EM maps, N99 and N529 glycans were resolved. The N99 glycan formed an H-bond with R102, while Δ242-243 enabled H245 to π-stack with R102 (Figure S11B). The N529 glycan projected toward N477 on an adjacent down-RBD (Figure 3I).

LP.8.1 lost N17 (due to T19I) but gained N31 (ΔS31), N188 (R190S), and N354 (K356T) (Figure S11A). N31 and N188 glycans were unresolved, reflecting local flexibility, whereas N354 was well resolved (Figure S11C). N354 glycosylation, conserved in BA.2.86-descended lineages, has been linked to closed-state stabilization and epitope masking.^58^ The independent acquisition of K356T in BA.3.2.2 suggests convergent evolution at this site.

Because N529 glycosylation is unique to BA.3.2 and located at the interprotomer interface, we tested its role by generating a BA.3.2.1 N529Q spike. Cryo-EM analysis showed that N529Q reduced the closed population from 82.5% in WT BA.3.2.1 to 59.8%, while increasing one RBD-up and flexible states to 22.2% and 18.0%, respectively (Figures 3J and S12A-S12F). 3DVA further confirmed enhanced conformational dynamics within the closed N529Q spike (Video S10). The closed N529Q structure also differed markedly from WT BA.3.2.1 (C_α_ RMSD = 4.19 Å), with RBD loops resembling WA1 and LP.8.1 rather than the remodeled RBD_C_ of WT BA.3.2.1 (Figures S12G-S12J; Tables S10 and S11). Together, these data indicate that the N529 glycan helps maintain the closed-state population and supports the distinctive closed geometry of BA.3.2.1 spike.

To define the structural basis of this effect, we compared N529Q with WT BA.3.2.1 and K852A using intra- and interprotomer PCA. In the intraprotomer PCA, N529Q showed a much larger shift from WT BA.3.2.1 than K852A (Figure S13A). Unlike the asymmetric protomers in WT BA.3.2.1, all three N529Q protomers clustered tightly at strongly negative PC1 values, indicating a more uniform and symmetric conformation (Figure S13B). The shift was driven primarily by SD2-centered changes, including altered θ_1_, θ_3_, θ_6_, d_4_/d_9_, and SD2-coupled torsions Φ_1_, Φ_2_, Φ_4_, and Φ_7_ (Figures S13C-S13E and Data S1), suggesting SD2-centered repacking after loss of the N529 glycan. K852A caused only minor intraprotomer changes.

Interprotomer PCA further separated N529Q, but not K852A, from WT BA.3.2.1 (Figure S13F). This separation reflected expanded RBD-RBD distances (d_1_′/d_2_′/d_3_′), shorter interprotomer RBD-NTD distances (d_4_′/d_5_′/d_6_′), and altered cross-protomer torsions, particularly Φ_1_′/Φ_2_′/Φ_4_′/Φ_5_′ (Figure S13G and Data S1). Thus, loss of the N529 glycan remodels trimer-apex packing across protomers.

PISA analysis showed that N529Q redistributed, rather than eliminated, inter-RBD contacts: A-B and C-A interfaces decreased, whereas the B-C interface increased; the closed K852A spike retained an inter-RBD interface pattern similar to WT BA.3.2.1 (Figure S13H). Although N529Q disrupted the N529 glycan contact with N477 on the neighboring RBD, new inter-RBD contacts, including N405-Y369 and Y505-Y369/N370 interactions, likely stabilized the remodeled trimer apex (Figures 2H, S13H, and S13J). Overall inter-RBD contacts in N529Q remained greater than those in non-BA.3.2.1 spikes.

Notably, the closed N529Q spike allowed modeling of nearly the entire FPPR, which adopted the canonical 3H-LHL configuration in all three protomers despite retention of K852, instead of the 6H-loop conformation seen in WT BA.3.2.1 (Figure S13K). Consistently, 3DVA indicated increased FPPR, SD1, and SD2 flexibility (Video S11). These results support a close coupling between the 6H-loop architecture and the tightly closed BA.3.2.1 spike conformation and indicate that K852 alone is insufficient to maintain the distinctive 6H-loop configuration.

### Less ACE2 binding to BA.3.2.1 spike than LP.8.1 spike

**Figure 4.**
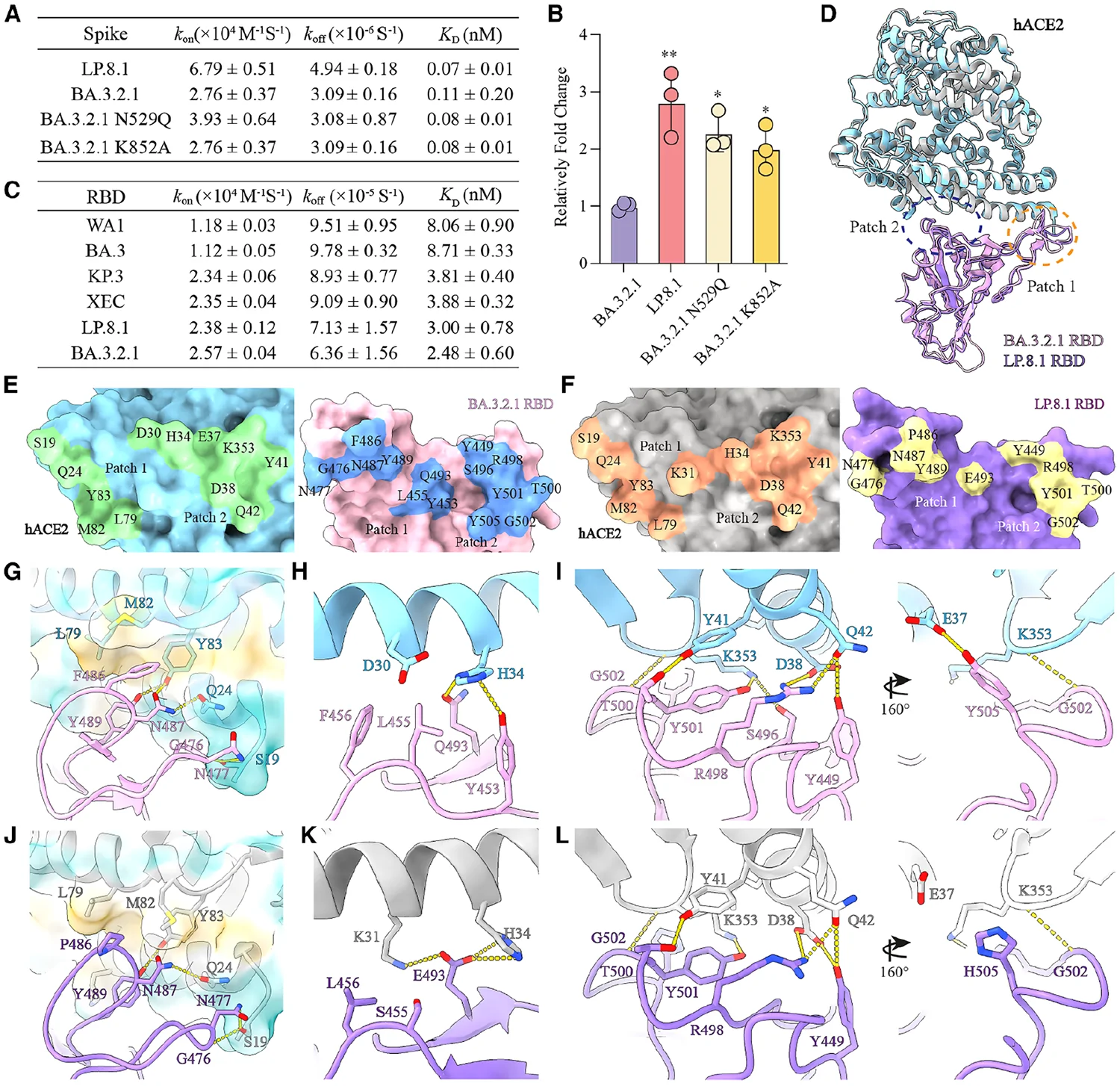
Interaction of BA.3.2.1 and LP.8.1 spike with hACE2 (A) BLI kinetics of spike trimer-hACE2 binding. Mean ± SD from three independent experiments is shown. (B) RT-qPCR quantification of virus binding to A549-ACE2 cells. Mean ± SD from three independent experiments is shown. *p < 0.05; **p < 0.01, one-way ANOVA with Dunnett’s multiple-comparison corrections (compared to BA.3.2.1). (C) BLI kinetics of RBD-hACE2 binding. Mean ± SD from three independent experiments is shown. (D) Structural alignment of hACE2-bound BA.3.2.1 and LP.8.1 RBDs, with patches 1 and 2 indicated. (E and F) Residue-level views of the BA.3.2.1 (E) and LP.8.1 (F) RBM-hACE2 interfaces. (G–I) Interaction networks mediating BA.3.2.1 RBD-hACE2 binding. (J–L) Interaction networks mediating LP.8.1 RBD-hACE2 binding. The hydrophobic hACE2 surface is highlighted. Complexes are shown as cartoons, with contact residues displayed as sticks. Dashed lines denote hydrogen bonds or salt bridges; hydrophobic contacts are indicated by residue proximity.

To define the functional consequences of the structural differences between BA.3.2.1 and LP.8.1 spikes, we compared their hACE2 binding by biolayer interferometry (BLI). BA.3.2.1 spike exhibited lower association (2.2-fold) and dissociation (1.6-fold) constants than LP.8.1, resulting in a 1.4-fold reduction in hACE2 affinity (Figures 4A, S14A, and S14B). Consistently, BA.3.2.1-spike virions attached less efficiently than LP.8.1-spike virions to A549-hACE2 cells at 4°C (Figure 4B).

To determine whether these differences were attributable to the RBD, we measured hACE2 binding to recombinant RBDs from BA.3.2.1, LP.8.1, BA.3, KP.3, XEC, and WA1. RBD affinity ranked BA.3.2.1 > LP.8.1 > XEC ≈ KP.3 > WA1 > BA.3 (Figure 4C and S14C-S14H). BA.3.2.1 RBD exhibited a *k*_on_ comparable to KP.3, XEC, and LP.8.1 but a markedly slower *k*_off_, explaining its stronger affinity. Notably, this high RBD affinity contrasted with reduced binding by the BA.3.2.1 spike, consistent with restricted RBD accessibility in its compact closed trimer.

We further tested BA.3.2.1 K852A and N529Q spikes, which shift the trimer toward more open conformations (Figures 3H and 3J). Both mutant spikes had hACE2 binding comparable to LP.8.1 (Figures 4A, S14I, and S14J). Consistently, N529Q or K852A virions showed a 2.3-fold and 2.0-fold higher attachment than WT BA.3.2.1 virions, respectively (Figures 4B and S15A). Together, these results indicate that reduced ACE2 binding by BA.3.2.1 spike is driven primarily by limited RBD accessibility imposed by its closed trimeric conformation.

### Virological characterization of N529 glycan and K852 within the loop FPPR

To define the function of the N529 glycan and K852, we introduced the N529Q or K852A mutation into the BA.3.2.1 spike mNG SARS-CoV-2 and characterized their virological properties (Figure S15A). Neither mutation altered S1/S2 cleavage (Figures S15B-S15D). However, K852A increased S2′ cleavage by 2.3-fold and reduced the spike-to-N ratio, whereas N529Q did not affect S2′ processing or spike-to-N ratio (Figures S15B and S15E-S15G).

We examined their impact on spike-mediated membrane fusion. Both mutations enhanced spike-mediated cell-cell fusion, increasing fusion by 1.9-fold for N529Q and 2.0-fold for K852A relative to WT BA.3.2.1 (Figures S15H and S15I). We then evaluated viral replication in Calu-3 cells. K852A replicated more efficiently than WT BA.3.2.1 at 48 and 72 h p.i., consistent with increased RBD accessibility, S2′ processing, and fusogenicity (Figure S15J). By contrast, N529Q did not alter viral growth, suggesting that other glycan-dependent effects may offset the fitness gains from increased RBD accessibility and fusion.

### Cryo-EM structures of BA.3.2.1 and LP.8.1 spike complexed with hACE2

BA.3.2.1 and LP.8.1 differ by >75 spike mutations, including many in the RBD (Figure S1). To understand their effects on receptor engagement, we determined cryo-EM structures of both spikes in complex with hACE2.

Initial incubation of BA.3.2.1 spike with hACE2 at a 1:2 molar ratio on ice yielded only apo-like closed and flexible particles (Figures S16A-S16C). Increasing hACE2 to a 1:3 ratio and incubating at 37°C produced predominantly hACE2-bound particles (Figures S16D and S16E). These were resolved into four conformations (Figures S16F-S16L; Table S4): three 3-RBD-up states with one to three hACE2 molecules bound, and one 2-RBD-up state with a single hACE2 bound (Figures S16I-S16L). In contrast, LP.8.1 spike formed hACE2-bound complexes on ice. Approximately half of the particles adopted 2-RBD-up states with one or two hACE2 molecules bound, whereas the remainder were unbound and resembled apo LP.8.1 spike (Figures S17A-S17E; Table S5).

Local refinement of the BA.3.2.1 RBD-hACE2 and LP.8.1 RBD-hACE2 interfaces yielded maps with 3.6 Å and 3.7 Å, respectively (Figures S16M, S16N, S17F, and S17G; Tables S8 and S9). These two interfaces were overall similar (C_α_ RMSD = 0.87 Å) and engaged hACE2 through two RBM contact patches spanning residues 437–451 and 498–507. However, their detailed RBM footprints differed (Figures 4D-4F).

Within the BA.3.2.1 RBM-hACE2 interface, patch 1 residues (G476, N477, F486, N487, Y489, L455, Q493, and Y453) contacted hACE2 residues S19, Q24, D30, H34, L79, M82, and Y83, whereas patch 2 residues (Y449, S496, R498, T500, Y501, G502, and Y505) engaged hACE2 residues E37, D38, Y41, Q42, and K353 (Figure 4E). BA.3.2.1 retained multiple conserved hACE2 contacts, including Y449-Q42, Y453-H34, L455-D30, F486-L79/M82/Y83, Y489-Y83, Q493-H34, T500-Y41, and G502-K353 (Figures 4G-4I). It also preserved the BA.3-associated affinity-enhancing contacts involving N477 (interacting with hACE2 S19), R498 (contacting hACE2 D38 and Q42), and Y501 (contacting hACE2 K353), lacked the hACE2-weakening E493,^60^ gained an S496-K353 contact, and retained the Y505-E37 interaction (Figure 4I).

LP.8.1 RBD engaged hACE2 through fewer residues in patch 1 (G476, N477, N487, Y489, and E493) and patch 2 (Y449, R498, T500, Y501, and G502). It shared several essential hACE2-contacting residues with BA.3.2.1, including Y449, G476, N477, N487, Y489, R498, T500, Y501, and G502 (Figure 4F). However, LP.8.1 used a distinct interaction network: P486 contacted hACE2 L79/M82/Y83, while the L455S/F456L “Slip” mutations positioned E493 to form a salt bridge with hACE2 K31 and interact with H34, thereby compensating for the hACE2-weakening E493 (Figures 4J and 4K). LP.8.1 also lacked BA.3.2.1 contacts with hACE2 K353 and E37 due to G496 and H505, respectively (Figure 4L). Collectively, these results indicated that BA.3.2.1 and LP.8.1 maintain a broadly conserved RBD-hACE2 binding architecture but use distinct RBM interaction networks.

### Structural basis for BA.3.2.1 spike’s antigenicity shift

**Figure 5.**
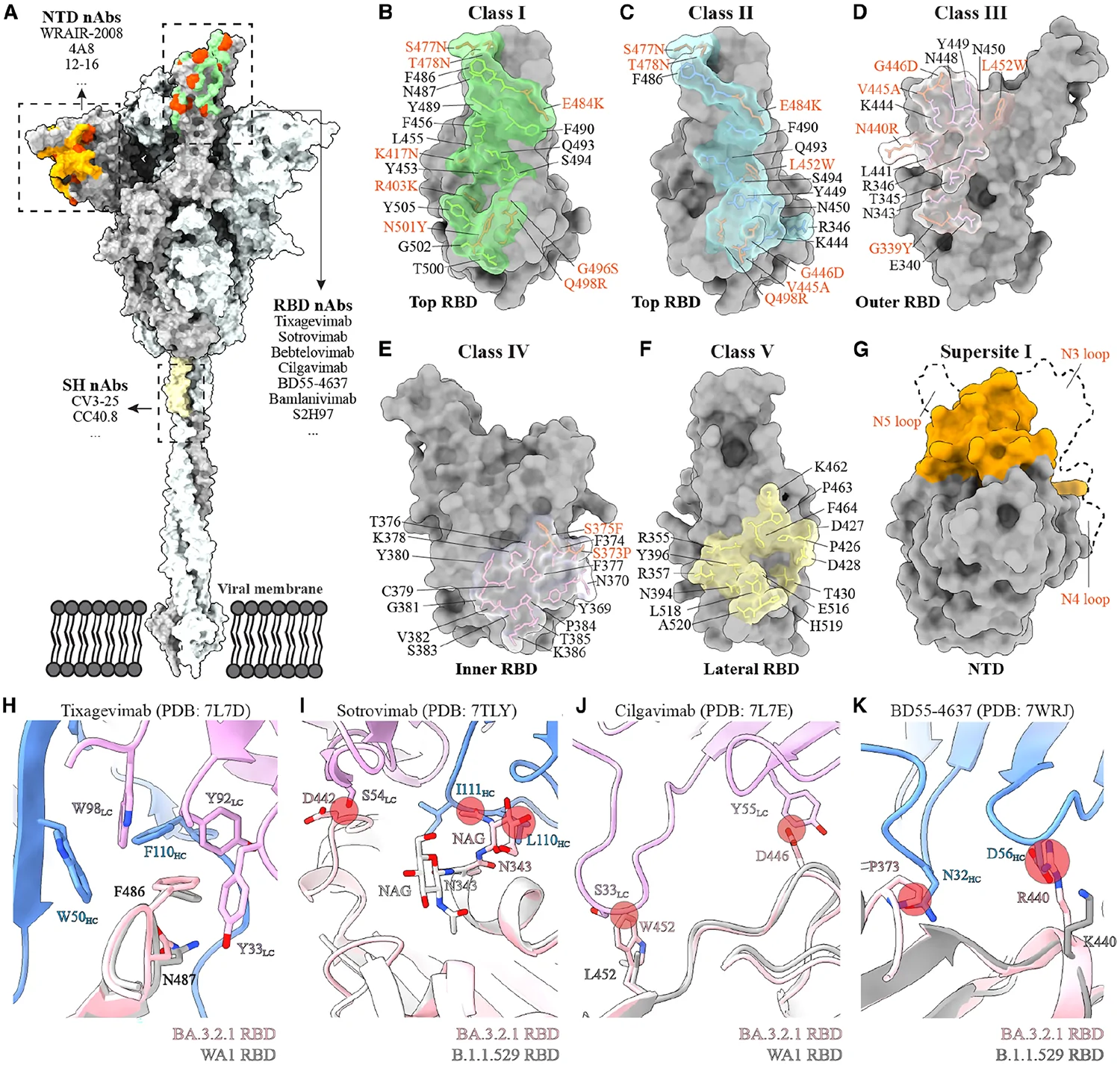
Structural basis for altered antigenicity of BA.3.2.1 spike (A) Representative NTD-, RBD-, and S2 stem-helix (SH)-targeting nAbs mapped onto open BA.3.2.1 spike. NTD supersite-1, RBD Class I-V, and SH antibody footprints are colored orange, green, and wheat, respectively; BA.3.2.1 mutations are highlighted in red. (B–F) Primary epitopes of class I (B), class II (C), class III (D), class IV (E), and class V (F) RBD-directed nAbs. BA.3.2.1 RBD is shown as a gray surface. Antibody footprints are shown as colored surfaces, epitope residues as sticks, and BA.3.2.1 substitutions within each epitope in red-orange. (G) Surface view of BA.3.2.1 NTD with supersite-1 highlighted in orange. The dashed outline indicates the corresponding supersite-1 region in WA1 NTD. (H–K) Close-up views of the BA.3.2.1 RBD superimposed on reference antibody complexes: WA1 RBD-Tixagevimab (H), B.1.1.529 RBD-Sotrovimab (I), WA1 RBD-Cilgavimab (J), or B.1.1.529 RBD-BD55-4637 (K). BA.3.2.1 residues predicted to interfere with antibody binding, and the corresponding antibody-contact residues are shown as sticks. Steric clashes are circled in red. Antibody heavy and light chains are colored blue and plum, respectively.

Consistent with our neutralization assays and prior reports, BA.3.2.1 showed a marked antigenicity shift (Figure 1L and 1M).^12,18,37,38^ To define the structural basis, we mapped BA.3.2.1 substitutions onto antibody-targeted spike regions (Figure 5A). BA.3.2.1 encodes 23 RBD substitutions that span epitopes of five major classes of RBD-directed nAbs, reshaping multiple antigenic surfaces (Figures 5B-5F).

For class I antibodies, which target the ACE2 ridge of up-RBDs,^32^ BA.3.2.1 carries R403K, K417N, S477N, T478N, E484K, G496S, Q498R, and N501Y within or near the antibody footprint (Figure 5B). The overall impact of these substitutions can vary by the exact epitope and angle of approach.^12^ The K417-E484-N501 escape-associated triad is expected to reduce many class I antibodies.^61^ However, tixagevimab (AZD8895) remained active against BA.3.2.1.^37^ Structural modeling suggests that tixagevimab binding is preserved because key F486/N487 contacts remain intact and BA.3.2.1 lacks Q493E/R, avoiding unfavorable electrostatic effects (Figure 5H and S18A).

For class II antibodies, which bind to lateral RBM,^25,62^ BA.3.2.1 substitutions V445A, G446D, L452W, S477N, T478N, E484K, and Q498R clustered at the interface (Figure 5C). These changes likely impair antibody binding through altered charge and steric effects. For example, E484K ablated bamlanivimab (LY-CoV555) by disrupting central RBM contacts (Figure S18B).

For class III antibodies, which bind to the outer RBD face, often at or near the N343-glycan patch and the N440-K444-V445-G446 loop region,^25,30^ BA.3.2.1 carries G339Y, N440R, V445A, G446D, and remodels the 438–451 and 497–505 loops (Figures 5D and S7A). These changes could (1) penalize Sotrovimab by perturbing the glycan environment and introducing steric clashes (Figures 5I and S18C), (2) reduce Bebtelovimab complementarity via V445A and loop shifts (Figure S18D), and (3) disrupt Cilgavimab binding through clashes (W452-S33/N34 and D446-Y55) in the RBM-shoulder (Figure 5J). These structural insights accord with their loss of activity against BA.3.2.^37^

For class IV antibodies, which target a largely conserved cryptic site near the RBD base (e.g., S373/S375/K378/Y380/V382/T385),^25,63^ BA.3.2.1 S373P remodels the 371–375 loop and introduces clashes predicted to disrupt BD55-4637 binding (Figures 5E and 5K), explaining the loss of neutralization for BD55-4637.^12,37^ In contrast, class V antibody epitopes are largely conserved,^32,64^ suggesting potential preservation of S2H97-like binding (Figures 5F and S18E). However, the closed BA.3.2.1 trimer may influence access to these cryptic epitopes.

BA.3.2.1 also extensively remodels the NTD antigenic supersite, including Δ136–147, Δ211, Δ242–243, and additional substitutions affecting the N3 and N4 loops (Figures 3B and 5G, and S18F-S18H). These changes are expected to disrupt binding by NTD-directed nAbs, such as 4A8, WRAIR-2008, and 12–16.^36,65,66^ Despite the absence of EM density in our maps, the conserved linear stem-helix epitope (residues 1,140–1,167), targeted by antibodies such as CV3-25 and CC40.8,^34,67^ remains unchanged (Figure S1). These antibodies were therefore expected to be unaffected.

Collectively, our analyses indicate that BA.3.2.1 broadly recontours RBD/NTD antigenic surfaces. In concert with its closed trimer, which may occlude cryptic/shoulder sites, these changes provide a structural explanation for the broad immune escape and antigenic shift of BA.3.2.1.

## DISCUSSION

BA.3.2 represents a divergent SARS-CoV-2 sublineage with antigenic and functional properties distinct from contemporaneous JN.1 descendants. Using BA.3.2.1 as a representative subvariant, we show that its heavily mutated spike balances broad immune escape with reduced replication fitness. Cryo-EM structures of apo and hACE2-bound BA.3.2.1 spike reveal a compact, glycan-reinforced closed trimer that limits receptor accessibility while shielding antibody-targeted epitopes. This trade-off likely contributed to the limited expansion of BA.3.2 relative to co-circulating JN.1 descendants.

In the mNG-SARS-CoV-2 backbone, BA.3.2.1 spike virus replicates less efficiently than JN.1 descendant spike viruses in Calu-3 cells and HAE, although it outcompeted BA.3 in HAE. These findings are consistent with the low prevalence of BA.3.2 sublineages since their emergence.^18^ Notably, replication among JN.1 descendants did not mirror their epidemiological succession, indicating that variant success is shaped by factors beyond spike-encoded replication properties, including non-spike mutations, host immunity, and ecological context.^68-70^

Spike cleavage during coronavirus entry is an important activation step that influences fusion, lung pathogenesis, and transmission.^71-73^ However, our data further indicate that spike cleavage alone does not predict viral fitness. Although S1/S2 and S2′ processing varied among variants, neither correlated with replication, cell-cell fusion, or epidemiological success. These differences may instead reflect variant-specific spike conformational dynamics that alter protease accessibility, as supported by increased S2′ cleavage after K852A-induced spike opening. Thus, spike processing contributes to viral entry but acts within a broader network of structural, cellular, and host determinants.

A central insight of this study is that BA.3.2.1 adopts a compact, “locked” spike architecture. Compared with LP.8.1, BA.3.2.1 shows reduced conformational heterogeneity, increased thermostability, contracted inter-RBD spacing, enhanced RBD-RBD contacts, altered SD1/SD2 organization, and a distinct FPPR architecture. The BA.3.2.1-specific N529 glycan further reinforces this closed state through interprotomer interactions. Disrupting this network by N529Q or K852A increased spike flexibility, enhanced receptor engagement, virion attachment, and cell-cell fusion, demonstrating that quaternary spike organization is a key regulator of BA.3.2.1 function.

This closed architecture provides a mechanistic explanation for the apparent disconnect between BA.3.2.1 RBD affinity and whole-spike ACE2 binding. Although the BA.3.2.1 RBD retained high intrinsic hACE2 affinity, the trimeric spike bound hACE2 less efficiently than LP.8.1, consistent with restricted RBD accessibility. Thus, BA.3.2.1 decouples intrinsic RBD affinity from trimer-level receptor engagement: the prefusion spike favors a closed state that limits premature receptor and antibody access, while individual RBDs retain sufficient affinity to support entry once exposed. Such conformational gating may underlie “high-escape/low-entry” phenotypes observed in some other Omicron subvariants.^12,74-77^

The same closed conformation likely contributes to immune escape by occluding cryptic and RBD-opening-dependent epitopes. In addition, BA.3.2.1 extensively remodels RBD and NTD antigenic surfaces through numerous substitutions and deletions, including changes in the NTD supersite such as Δ136-147 and Δ242-243. These alterations likely reduce recognition by both RBD- and NTD-directed neutralizing antibodies,^78,79^ and may also affect NTD-RBD crosstalk, sialic-acid binding, and modulation of entry and fusogenicity.^80-82^ Together, epitope remodeling and closed-state shielding provide a structural basis for the marked antigenic shift of BA.3.2.1.

Our study also highlights the important role of N-glycosylation in balancing immune evasion and viral fitness. The N529 glycan stabilizes the closed BA.3.2.1 spike and limits receptor accessibility while contributing to antibody shielding.^83^ However, N529Q did not improve replication in Calu-3 cells despite increasing receptor engagement and fusion. This suggests that gains in accessibility may be offset by loss of glycan-dependent functions or broader structural rearrangements that compromise spike performance.

In summary, BA.3.2.1 achieves broad neutralizing-antibody escape through extensive RBD and NTD remodeling and a glycan-reinforced quaternary architecture. However, this architecture restricts RBD accessibility and imposes a fitness cost, providing a structural-functional explanation for the persistent but limited expansion of BA.3.2 relative to JN.1 descendants. Continued surveillance will be essential to identify future variants that may rebalance immune escape and replication through compensatory adaptation.

### Limitations of the study

This study has several limitations. First, the mNG SARS-CoV-2 does not capture potential contributions of non-spike genes or spike/non-spike epistasis. Nevertheless, the strong concordance between spike structure and phenotype supports spike as a major, though not exclusive, determinant of the observed differences. Second, our serum cohorts were limited to KP.2- or KP.3-infected individuals from a single geographic region. Vaccination history, time since infection, and hybrid immunity may influence neutralization breadth. Third, we did not assess mucosal immunity or T cell responses, which also contribute to protection independently of neutralizing antibodies. Fourth, site-specific glycoproteomic analysis will be needed to define glycan occupancy and microheterogeneity and their roles in conformational regulation and epitope masking. Finally, although cryo-EM captured multiple apo and ACE2-bound spike states, time-resolved studies are needed to define spike-opening kinetics and membrane-fusion dynamics. Continued characterization of other emerging subvariants, including BA.3.2.2, NB.1.8.1, and XFG, will be important for monitoring SARS-CoV-2 evolution.

## STAR★METHODS

### EXPERIMENTAL MODEL AND STUDY PARTICIPANT DETAILS

Cell lines used in this study are listed in the key resources table. Cell growth conditions can be found in the method details.

#### Ethical statement

The use of human serum specimens in this study was reviewed and approved by the University of Texas Medical Branch (UTMB) Institutional Review Board (IRB number 20–0070). No informed consent was required because these deidentified sera were leftover specimens from the routine standard of care and diagnostics. No diagnosis or treatment was involved either.

All virus infections were conducted in a biosafety level 3 (BSL-3) facility with redundant fans in the biosafety cabinets at UTMB. All personnel wore powered air-purifying respirators (Breathe Easy, 3M) with Tyvek suits, aprons, booties, and double gloves.

#### Cells

Vero E6 (ATCC CRL-1586) cells, Calu-3 cells (ATCC HTB-55), and 293T cells (ATCC CRL-3216) were obtained from the American Type Culture Collection (ATCC, Bethesda, MD). 293T-hACE2 cells were generated using a lentiviral hACE2 expression vector kindly provided by Dr. Weiyi Peng from the University of Houston. Vero E6 cells expressing TMPRSS2 (JCRB1819) were purchased from SEKISUI XenoTech, LLC. All cells were cultured in Dulbecco’s modified Eagle’s medium (DMEM) supplemented with 10% fetal bovine serum (FBS; HyClone Laboratories, South Logan, UT) and 1% penicillin/streptomycin (P/S). The A549 cells stably expressing hACE2 were generated previously^93^ and cultured in the culture medium supplemented with 10 μg/mL Blasticidin (Thermo Fisher Scientific). All cultures were maintained at 37°C with 5% CO_2_. The 293F Freestyle cells were obtained from Thermo Fisher Scientific and maintained in FreeStyle 293 Expression Medium at 37°C with 8% CO_2_. Cells were tested Mycoplasma negative by nested PCR at the Tissue Culture Core Facility (TCCF) at UTMB.

#### Human serum

Two panels of human sera collected at UTMB were used in the study. The first sample panel, consisting of 26 sera, was collected 21–99 days (median 42.5) after KP.2 infection (as determined by RT-PCR) from individuals aged 22–82 years (median 38.5) who had received 0–4 doses of mRNA vaccines. The second sample panel, consisting of 21 sera, was collected 23–105 days (median 50) after KP.3 infection (as determined by RT-PCR) from individuals aged 26–96 years (median 54) who had received 0–6 doses of mRNA vaccines. Patient information was completely deidentified from all specimens. The de-identified human sera were heat-inactivated at 56°C for 30 min before the neutralization test. Tables S1 and S2 show the summary of serum information.

### METHOD DETAILS

#### Generation of recombinant SARS-CoV-2 mutant viruses

Recombinant BA.3.2.1 (GISAID: EPI_ISL_19771108)-, XEC (GISAID: EPI_ISL_19283891)-, LP.8.1 (GISAID: EPI_ISL_19674199)-mNG SARS-CoV-2 spike-variants were constructed by engineering the complete *spike* gene from the indicated variants into an infectious cDNA clone of mNG USA-WA1/2020 as reported previously.^39^ Briefly, standard overlap PCRs were conducted to introduce the spike mutations into the infectious clone of mNG USA-WA1/2020. The full-length infectious cDNA clones were assembled by *in vitro* ligation. Subsequently, the genome-length RNAs were synthesized by *in vitro* transcription. The full-length RNA and N gene RNA transcripts were electroporated into Vero E6-TMPRSS2 cells to rescue the recombinant viruses. After 48–72 h of transfection, the supernatants (referred to as P0) were harvested. The P0 stocks were then inoculated into freshly prepared Vero E6 cells for further amplification. At 48 h p.i., supernatants (P1) were harvested, clarified by centrifugation at 1000 × g for 10 min, and stored at −80°C.

#### RNA extraction, RT-PCR, and cDNA sequencing

Cell culture supernatants were mixed with a 5-fold excess of TRIzol LS Reagent (Invitrogen). Viral RNAs were extracted according to the manufacturer’s instructions. The extracted RNAs were dissolved in 50 μL nuclease-free water. For sequence validation of mutant viruses, 10 ng of RNA samples were used for reverse transcription by using the SuperScript IV One-Step RT-PCR System (Thermo Fisher Scientific) with CoV-21115V and CoV-YH5 (Table S12). The resulting DNAs were purified by the QIAquick Gel Purification Kit (Qiagen) and sequenced by Sanger sequencing at GENEWIZ (South Plainfield, NJ).

#### Plaque assay

1 × 10^6^ Vero E6-TMPRSS2 cells per well were seeded into 6-well plates. The next day, 200 μL of 10-fold serially diluted virus was added to pre-seeded cells and incubated at 37°C for 1 h. After that, the inoculum was replaced with 2 mL of overlay medium containing DMEM with 2% FBS, 1% penicillin/streptomycin, and 1% SeaPlaque agarose (Lonza, Walkersville, MD, USA). After 2 days of incubation at 37°C, 2 mL of overlay medium supplemented with neutral red (Sigma-Aldrich, St. Louis, MO, USA) was added to stain the cells, and plaques were counted on the next day.

#### Growth kinetics of recombinant SARS-CoV-2 in cell culture

Approximately 3 × 10^5^ Calu-3 cells were seeded into each well of 12-well plates and cultured at 37°C, 5% CO_2_ for 24 h. WA1, BA.3, BA.3.2.1, KP.3, XEC, or LP.8.1 was inoculated into the cells at an MOI of 0.05. Back titration of the inoculum confirmed an equal amount of input viruses. Following virus adsorption, cells were washed three times with PBS (each time 1 mL) to remove unbound virus before adding fresh medium. The virus was incubated with the cells at 37°C for one hour. After infection, the cells were washed 3 times with DPBS to remove unattached virus. One milliliter of culture medium was added to each well to maintain the cells. At each time point, 200 μL of culture supernatant was collected for the plaque assay. Meanwhile, 200 μL of fresh medium was added to each well to replenish the culture volume. The cells were infected with triplicate for each virus. All samples were stored at −80°C until plaque analysis.

#### Virion purification and spike protein cleavage analysis

One milliliter of each virus from the P1 stocks collected from Vero E6 cells was combined with polyethylene glycol (PEG)-8000 (Sigma-Aldrich) to achieve a final concentration of 10% w/v. The mixtures were incubated at room temperature for 30 min, then centrifuged at 4000 × g for 10 min to pellet the virions. The resulting pellets were washed once with 70% ethanol and subsequently resuspended in 2 × Laemmli sample buffer (Bio-Rad) containing 0.7 M β-mercaptoethanol (Sigma-Aldrich). Samples were heat-in-activated at 95°C for 15 min, centrifuged at 13,000 rpm for 10 min, and then separated on a 4–15% gradient SDS-PAGE gel. Viral spikes and nucleocapsid proteins were detected by Western blot using specific antibodies against S1, S2, and N, respectively. Densitometric quantification was performed using ImageJ.^92^

#### Fluorescent focus reduction neutralization test (FFRNT)

Neutralization titers of human sera were evaluated using FFRNT against BA.3-, BA.3.2.1-, KP.3-, KP.2-, XEC-, and LP.8.1-spike mNG SARS-CoV-2s, following a previously established FFRNT protocol.^13^ In brief, 3 × 10^4^ Vero E6 cells were plated per well in 96-well plates (Greiner) and incubated overnight. The following day, sera were serially diluted 2-fold starting at 1:20, covering a final dilution range of 1:20 to 1:20,480. Each diluted serum sample was mixed with 100–150 focus-forming units (FFUs) of mNG SARS-CoV-2 and incubated at 37°C for 1 h. Afterward, the mixtures were added to the Vero E6 cell monolayers. After a 1-h infection period, the inoculum was removed and replaced with 100 μL overlay medium containing 0.8% methylcellulose. Plates were then incubated at 37°C for 16 h. Fluorescent foci were imaged using a Cytation 7 system (BioTek) equipped with a 2.5× FL Zeiss objective, and images were processed via Gen5 software with settings optimized for GFP detection (wavelengths 469–525 nm, threshold 4000, object size 50–1000 μm). The number of foci per well was quantified and normalized to controls without serum to calculate relative infectivity. The FFRNT_50_ titer was defined as the lowest serum dilution reducing fluorescent foci by more than 50%. Each serum was tested in duplicate, and the geometric mean was calculated. Data visualization was performed in GraphPad Prism 9, and figures were prepared using Adobe Illustrator. For analysis and plotting, an FFRNT_50_ of <20 was assigned a value of 10.

#### Pairwise competition experiment

The human primary airway epithelial (HAE) culture (EpiAirway) was purchased from MatTek. The 3D mucociliary tissue model is generated by differentiating primary normal human tracheal/bronchial epithelial cells on a semi-permeable PermaCell insert (MatTek) under air-liquid interface (ALI) conditions. According to the manufacturer’s instructions, HAEs were maintained in EpiAirway Maintenance Medium (MatTek) in a humidified incubator at 37°C with 5% CO_2_. After receiving, the tissues were maintained in the laboratory for one week prior to use.

The titers of viral stocks were determined by plaque assays on Vero E6-TMPRSS2 cells using the same assay batch. At least three technical replicates were performed, and the average titer was used to calculate input virus volumes for competition experiments. All viruses were normalized to 1 × 10^7^ PFU/mL in culture medium containing 2% FBS. For inoculum preparation, 100 μL of each virus was mixed with 800 μL DPBS to yield a 1:1 PFU ratio. An aliquot of the virus mixture was stored to verify the input ratio of the two variants.

Before infection, HAE cultures were incubated with DPBS at 37°C for 30 min. Following removal of DPBS, 200 μL of the viral inoculum was applied to the apical surface of each well. After a 2-h incubation at 37°C with 5% CO_2_, the inoculum was removed, and cultures were washed three times with DPBS to eliminate unbound viruses. At each collection time point, 300 μL of DPBS was added to the apical surface and incubated at 37°C with 5% CO_2_ for 30 min to recover released viruses. The DPBS washes were collected into 2-mL tubes. On day 4 post-infection, after collecting the apical washes, 300 μL of TRIzol reagent (Invitrogen) was added to the cells to lyse them and preserve total cellular RNA. All samples were stored at −80°C prior to processing.

For analysis, 100 μL of each sample was mixed with TRIzol LS reagent at a 1:5 ratio. Viral RNA was extracted using the Direct-zol RNA Miniprep Plus kit (Zymo Research) following the manufacturer’s protocol. To identify and differentiate between the variants, cDNA fragments (100–200 bp) containing strain-specific mutations were amplified using the SuperScript IV One-Step RT-PCR System with designated primer pairs. Primer set 1: K529N-F and K529N-R targeted *spike* gene residues 499–570, distinguishing BA.3 from BA.3.2.1. Primer set 2: A852K-F and A852K-R amplified residues 823–883 to differentiate BA.3.2.1 from LP.8.1. Primer set 3: K1086R-F and K1086R-R covered residues 1045–1122, distinguishing LP.8.1 from XEC. Primer set 4: F59S-F and F59S-R targeted residues 31–97 to differentiate KP.3 from XEC. Primers are listed in Table S12. The resulting cDNA amplicons were purified via gel extraction and submitted for Illumina next-generation sequencing (NGS) at the UTMB sequencing core facility.

#### Cell-cell fusion

The cell-cell fusion assay was performed using a previously described protocol.^43^ The cDNAs encoding the spike of WA1, BA.3, LP.8.1, BA.3.2.1, and BA.3.2.1 with N529Q or K852A mutation were cloned into the pXJ vector.^85^ The 293T cells were co-transfected with plasmids expressing spikes and pXJ-eGFP (donor cells). At 24 h post-transfection, cells were detached using trypsin and co-cultured with 293T-ACE2 cells (recipient cells) at 15,000 cells each per well in a 96-well plate. After 16 h of co-culture, cells were stained with Hoechst 33342 and imaged using an ImageXpress Confocal HT system. ImageJ was used to quantify GFP-positive areas. Only GFP-positive areas larger than 800 μm^2^ were scored as fusion events, and the group using GFP-alone as donor cells was used to define the background. Twelve experimental replicates from each group were used for quantification and analysis.

#### Protein expression and purification

The cDNAs encoding the ectodomain (residues 1–1208) of BA.3.2.1 (GISAID: EPI_ISL_19771108) and LP.8.1 spike (GISAID: EPI_ISL_19674199) with HexaPro (F817P, A892P, A899P, A942P, K986P, and V987P), a mutated fusion cleavage site (_682_GSAS_685_), and a C-terminal T4 fibritin trimerization domain, a strep-tag II, and a 6x His tag, were cloned into the mammalian cell expression vector pαH.^23,77^ The BA.3.2.1 spike carrying the N529Q or K852A mutation was generated by site-directed mutagenesis using primer N529Q-F/N529Q-R or K852A-F/K852A-R (Table S12) and cloned into the same vector. To express the spike protein, 500 mL FreeStyle 293-F cells were transiently transfected with 0.5 mg of plasmid using Polyethylenimine Hydrochloride 40,000 (Polysciences). Cultures were harvested five days after transfection, and the supernatant was collected by centrifugation. The secreted S protein in the supernatant was passed through a 0.22 μm filter and purified using Ni-NTA agarose (Qiagen). Spike protein was further purified by size-exclusion chromatography using a Superose 6 10/300 column (Cytiva) in a buffer composed of 20 mM Tris, pH 8.0, and 200 mM NaCl.

A gene encoding human ACE2 (1–615) with a C-terminal 6xHis tag was cloned into vector pCAG.^84^ The expression vectors were transiently transfected into FreeStyle 293-F cells as described above. Four days after transfection, the supernatant was harvested. The secreted ACE2 protein in the supernatant was passed through a 0.22 μm filter and purified using Ni-NTA agarose. ACE2 protein was further purified by size-exclusion chromatography using a Superdex 200 10/300 column (Cytiva) in a buffer composed of 20 mM Tris, pH 8.0, 150 mM NaCl.

The RBD (residues R319-F541) of WA1, BA.3, BA.3.2.1, KP.3, XEC, and LP.8.1 spike with an N-terminal signal peptide for secretion and a C-terminal 6×His tag for purification was inserted into the vector pCAG.^75^ The secreted RBD protein in the supernatant was passed through a 0.22 μm filter and purified using Ni-NTA agarose columns. RBD protein was further purified by size-exclusion chromatography using a Superdex 200 10/300 column in a buffer composed of 20 mM Tris, pH 8.0, 150 mM NaCl.

#### Cryo-EM sample preparation and imaging

For spike protein alone, a 3 μL sample at 1.3 mg/mL was applied to QUANTIFOIL R1.2/1.3 grid (Electron Microscopy Sciences) that had been plasma-cleaned by PELCO easiGlow Glow Discharge Cleaning System (TED PELLA, Inc.). The grid was blotted for 2 s and plunge-frozen in liquid ethane using Vitrobot Mark IV (Thermo Fisher Scientific) at 8°C and 100% humidity. For LP.8.1 spike-hACE2 complex, hACE2 was mixed with the spike at a 2:1 molar ratio to a final spike concentration of 1.2 mg/mL and incubated on ice for 30 min 3 μL samples were applied to the grid as described above. For the BA.3.2.1 spike-hACE2 complex, hACE2 was mixed with the spike at a molecular ratio of 3:1 to a final concentration of spike at 1.2 mg/mL and incubated at 37°C for 15 min, followed by incubation on ice for 30 min 3 μL samples were applied to the grid as described above.

Grids were loaded on either a Titan Krios G3i microscope (Thermo Fisher Scientific) at the UTMB Sealy Center for Structural Biology or a Titan Krios G4 microscope (Thermo Fisher Scientific) at the Texas A&M Laboratory for Biomolecular Structure and Dynamics. Both microscopes were operated at 300 keV and equipped with a K3 direct electron detector. The G3i microscope included a GIF Quantum energy filter (20-eV energy slit) (Gatan). The G4 microscope included a BioContinuum post-column imaging filter. Cryo-EM data were automatically acquired with a K3 camera using SerialEM (G3i) or EPU (G4) at a nominal magnification of 105,100× (corresponding to 0.832 Å per pixel) with a nominal defocus range of −0.9 to −2.5 μm. Forty-frame movie stacks were collected over an exposure time of one second with a total dose of 39.8–41.0 e^−^ Å^−1^. The detailed data collection parameters were summarized in Tables S5, S6, and S10.

#### Cryo-EM data processing

Collected movie frames were imported into CryoSPARC^91^ for image processing. All reconstructions were performed using C1 symmetry to avoid imposing symmetry-related bias and to capture potential structural heterogeneity. Movie data were motion-corrected using Patch Motion Correction. The contrast transfer function (CTF) was estimated using Patch CTF Estimation. Micrographs with CTF fit worse than 5 Å were excluded.

For BA.3.2.1 apo spike, 4,474,773 particles were selected from 12,806 micrographs using Template Picker and extracted with 2.1 × binning. 1,411,706 particles were selected after three rounds of two-dimensional (2D) classification. 400,000 particles were used to create four initial three-dimensional (3D) volumes using Ab Initio, followed by iterative heterogeneous refinement using all the particles. This yielded two conformations: closed, with 3-RBD-down; flexible, with 2-RBD-down and one RBD flexible. Particles were re-extracted without binning and refined by homogeneous refinement, global and local CTF refinement, and non-uniform refinement, yielding 2.6 Å and 3.0 Å reconstructions for closed and flexible conformations, respectively. To further resolve the NTD and RBD of the BA.3.2 apo spike, local refinement was performed using the closed conformation. Masks covering RBD_A_/RBD_C_/NTD_B_, RBD_A_/NTD_C_, RBD_B_/NTD_A_, and NTD_B_ were created in UCSF Chimera^86^ and were imported into cryoSPARC with dilation and soft padding. Local refinement was performed with particle re-centering, yielding 2.9–3.0 Å reconstructions.

For LP.8.1 apo spike, a total of 13,741,340 particles were selected from 15,414 micrographs using Template Picker and extracted with 2.1 × binning. 3,618,231 particles were selected after three rounds of 2D classification. Ab Initio reconstruction using 400,000 particles, followed by iterative heterogeneous refinement of all retained particles, identified three conformations: closed, 3-RBD-down; open, 1-RBD-up; flexible, 2-RBD-down and 1-RBD-flexible. Particles were re-extracted without binning and refined by homogeneous refinement, global and local CTF refinement, and non-uniform refinement, yielding 2.5 Å, 2.5 Å, and 2.6 Å reconstructions for closed, open, and flexible conformations, respectively. To further resolve the NTD and RBD of the LP.8.1 apo spike, local refinement was performed using the closed conformation. Masks covering each down RBD and its neighboring NTD were created using UCSF Chimera and were imported into CryoSPARC with dilation and soft padding. Local refinement was performed with re-centering, yielding 2.8 Å reconstructions.

For BA.3.2.1 spike with the N529Q mutation, a total of 7,289,403 particles were selected from 8,637 images using Template Picker and extracted with 2.2 × binning. 1,279,757 particles were selected after three rounds of 2D classifications. Ab Initio reconstruction using 400,000 particles, followed by iterative heterogeneous refinement of all retained particles, identified three conformations: closed, 3-RBD-down; open, 1-RBD-up; flexible, 2-RBD-down and 1-RBD-flexible. Particles were re-extracted without binning and refined by homogeneous refinement, global and local CTF refinement, 3D classification, and non-uniform refinement, yielding 2.4 Å, 2.7 Å, and 2.9 Å reconstructions for closed, open, and flexible conformations, respectively. To further resolve the inter-RBD interaction interface, Masks covering RBD_C_/NTD_B_ were created using UCSF Chimera and were imported into CryoSPARC with dilation and soft padding. Local refinement was performed with re-centering, yielding 2.8 Å reconstructions.

For BA.3.2.1 spike with the K852A mutation, a total of 7,289,403 particles were selected from 8,637 images using Template Picker and extracted with 2.2 × binning. 1,279,757 particles were selected after three rounds of 2D classifications. Ab Initio reconstruction using 400,000 particles, followed by iterative heterogeneous refinement of all retained particles, identified three conformations: closed, 3-RBD-down; open, 1-RBD-up; flexible, 2-RBD-down and 1-RBD-flexible. Particles were re-extracted without binning and refined by homogeneous refinement, global and local CTF refinement, 3D classification, and non-uniform refinement, yielding 2.4 Å, 2.7 Å, and 2.9 Å reconstructions for closed, open, and flexible conformations, respectively. To further resolve the inter-RBD interface, masks covering RBDA/RBDC/NTDB were created using UCSF Chimera and were imported into CryoSPARC with dilation and soft padding. Local refinement was performed with re-centering, yielding 2.8 Å reconstructions.

For the BA.3.2.1 spike/hACE2 complex, a total of 4,539,114 particles were selected from 12,806 micrographs using Blob Picker and extracted with 2.2 × binning. 979,353 particles were selected after three rounds of 2D classifications. Ab Initio reconstruction using 400,000 particles followed by iterative heterogeneous refinement of all retained particles, resulting in four conformations: conformation 1, 3-RBD-up, all were bound by hACE2; conformation 2, 3-RBD-up, one of the up-RBD was bound by hACE2; conformation 3, 3-RBD-up, two of the up-RBD was bound by hACE2; conformation 4, 2-RBD-up, one of the up-RBD was bound by hACE2. Particles were re-extracted without binning and refined by homogeneous refinement, global and local CTF refinement, 3D classification, and non-uniform refinement, yielding 3.3 Å, 3.2 Å, 3.5 Å, and 3.7 Å reconstructions for conformations 1–4, respectively, according to GSFSC at 0.143. To further resolve the binding interface between ACE2 and RBD, local refinement was performed using particles from conformational 1. A mask was created using UCSF Chimera that covers the up-RBD and ACE2 and was imported into CryoSPARC with dilation and soft padding. Local refinement was performed with particle re-centering, yielding a 3.6 Å reconstruction. The map was further sharpened using DeepEMhancer.

For LP.8.1 spike/hACE2 complex, a total of 3,194,764 particles were selected from 8,294 micrographs using Template Picker and extracted with 2.2 × binning. 1,140,024 particles were selected after three rounds of 2D classification. Ab Initio reconstruction using 400,000 particles, followed by iterative heterogeneous refinement of all retained particles, resulted in four conformations. Conformations 1 and 2 are LP.8.1 spike/hACE2 complex: conformation 1, 2-RBD-up, one of the up RBDs was bound by hACE2; conformation 2 also has two up RBDs, and both are bound by hACE2. Conformations 3 and 4 are apo spike: conformation 3 has one up RBD, and conformation 4 is in a closed conformation. Particles were re-extracted without binning and refined by homogeneous refinement, global and local CTF refinement, 3D classification, and non-uniform refinement, yielding 2.8 Å, 3.8 Å, 2.8 Å, and 3.2 Å reconstructions for conformations 1–4, respectively. To further resolve the RBD-hACE2 interface, local refinement was performed using particles from conformation 1. A mask covering the up-RBD with hACE2 binding was created using UCSF Chimera and imported into CryoSPARC with dilation and soft padding. Local refinement was performed without particle subtraction, yielding a 3.7 Å reconstruction. The map was further sharpened using DeepEMhancer. Local resolutions of final reconstructions were estimated by CryoSPARC’s blocres and displayed as resolution ranges in UCSF ChimeraX.

#### Model building, refinement, and analysis

Cryo-EM structures of the BA.3 spike (PDB: 7XIY) and KP.3.1.1 spike (PDB: 9ELI, 9ELH) were used to build an initial model of the apo BA.3.2.1 spike and the apo LP.8.1 spike, respectively. The initial model was built by rigid body fitting in UCSF Chimera and manual adjustments in Coot.^88^ The model was refined iteratively by real-space refinement in Phenix^89^ and by manual adjustments and improvements in Coot. Initial model building for BA.3.2.1 RBD/hACE2 complex, LP.8.1 RBD/hACE2 complex was built by rigid body fitting of BA.3.2.1 RBD, LP.8.1 RBD, and hACE2 in UCSF Chimera and manual adjustments in Coot. The model was refined as described above. The full-length BA.3.2.1 NTD and LP.8.1 NTD models were built using the Predict and Build tool in Phenix.^51^ The full-length BA.3.2.1 spike model was built as reported previously.^94^ For 3D variability analysis, EM maps of spikes were separately analyzed in CryoSPARC with resolution filtered at 5 Å. A movie of the component was generated in UCSF Chimera using frames from the 3DVA display program in simple mode in CryoSPARC (Videos S1, S2, S3, S4, S5, S6, S7, S8, S9, S10, and S11).^95^ Protein-protein interaction was analyzed using UCSF ChimeraX and PISA.^96^ Figures and movies were prepared using UCSF ChimeraX and Chimera. Final map and model statistics are summarized in Tables S5, S6, and S8-S11.

#### Negative-stain electron microscopy

BA.3.2.1 spike was incubated with hACE2 at a molar ratio of 1:3 at 37°C for 15 min. A 4 μL sample at 0.05 mg/mL was applied to a glow-discharged CF200-Cu carbon film grid (Electron Microscopy Sciences). The sample was left on the carbon film for 60 s, followed by negative staining with 2% uranyl formate for 60 s. Micrographs were recorded on a JEOL 2100FS (JEOL) microscope at 80,000× magnification, operated at 200 keV. The images were imported and processed in CryoSPARC.

#### Vector-based analysis

Vector analysis of intraprotomer and interprotomer domain positions was performed using the Visual Molecular Dynamics (VMD)^90^ sarbecovirus neutralizing developed by Rory Henderson et al.^45,46^ Briefly, a central co-ordinate was assigned to each spike domain. For cross-structure comparison, the protomer with the greatest conformational deviation was designated protomer B; A and C were then labeled clockwise from B when viewed from the extracellular side. For each protomer, Cα centroids were determined for the NTD (residue 27–43 and 54–271), NTD′ (residue 44–53 and 272–293), NTD sheet motif (NTDa, residue 116–129 and 169–172), NTD′ residue 276 (NTD′a), RBD (residue 330–443 and 503–528), RBD helix motif (RBDa, residue 403–410), SD1 (residue 323–329 and 529–590), SD1 residue 575 (SD1a), SD2 (residue 294–332, 591–620, 641–691, and 692–696), SD2 residue 671 (SD2a), CD (residue 711–716 and 1072–1121), and S2 sheet motif (S2s, residue 717–727 and 1047–1071). Intraprotomer vectors were calculated between the following within protomer centroids: NTD to NTD′, NTD′ to SD2, SD2 to SD1, SD2 to CD, SD1 to RBD, CD to S2s, NTDa to NTD, and RBD to RBDa. Vector magnitudes (d1-d10), angles (θ_1_-θ_8_), and dihedrals (Φ_1_-Φ_7_) were determined from these vectors and centroids (Table S6). PCA was performed in R with the vector data centered and scaled. Spike structures from WA1 (PDB 6VXX), BA.3 (PDB 7XIY), D614G (PDB 7KRQ), and KP.3.1.1 (PDB 9ELI, closed; 9ELH, open) were included for analysis.

Interprotomer vectors were calculated for the following: NTD′ to NTD′a, NTD′ to SD2, SD2 to SD2a, SD2 to SD1, SD1 to SD1a, and SD1 to NTD′. Vectors for the RBD to the adjacent RBD and RBD to the adjacent NTD were calculated using the above RBD, NTD, and RBDa centroids. Vectors were calculated for the following: RBD_A_ to RBD_C_, RBD_C_ to RBD_B_, and RBD_B_ to RBD_A_. Distances (d_1_′-d_9_′), angles (θ_1_′-θ_3_′), and dihedrals (Φ_1_′-Φ_9_′) were determined from these vectors and centroids (Table S7). We included the same set of closed spikes of WA1 (PDB 6VXX), BA.3 (PDB 7XIY), D614G (PDB 7KRQ), and KP.3.1.1 (PDB 9ELI) for this analysis. Computed descriptor values were shown in Data S1. R scripts used for PCA have been deposited at Zenodo (DOI: https://doi.org/10.5281/zenodo.20615912).

#### Circular dichroism

The thermal stability of BA.3.2.1 and LP.8.1 spike proteins was assessed by circular dichroism using a JASCO J-815 spectropolarimeter (Jasco). Protein samples were prepared at a final concentration of 0.1 mg/mL in PBS. CD spectra were recorded at 222 nm. A thermal melt was performed from 25°C to 85°C, increasing the temperature in 1°C increments, and the CD signal was monitored at each step after equilibration for 1 s. The resulting melting curve was fitted to determine the protein’s thermal denaturation midpoint (Tm).

#### Bio-layer interferometry analysis

The recombinant, purified RBD or spike proteins were used to measure affinity with hACE2 on the Octet R8 (Sartorius). For RBD/hACE2 interaction, 2 μg ACE2 was biotinylated with NHS-PEG_4_-Biotin (Thermo Fisher Scientific) and captured onto the SA sensor (Sartorius) for 300 s. The loaded biosensors were then quenched and dipped into PBS buffer for 60 s to adjust baselines. Subsequently, the biosensors were dipped into serially diluted RBD proteins (100~3.7 nM) for 300 s to record association kinetics and then dipped into PBS buffer for 600 s to record dissociation kinetics. PBS buffer without RBD was used as a background. For spike/hACE2 interaction, 6 μg of biotinylated hACE2 proteins were captured onto the SA sensor (Sartorius) for 300 s. The loaded biosensors were then quenched and dipped in PBS buffer for 60 s to adjust baselines. Subsequently, the biosensors were dipped into serially diluted spike proteins (50~1.9 nM) and then dipped into PBS buffer containing 0.5% Tween 20 for 600 s to record dissociation kinetics. All steps were performed at 25°C with shaking. Data was analyzed using the Octet Data Analysis software V13 (Sartorius), which was used to fit the curve using a 1:1 binding model and the global fitting method.

#### Virus attachment and RT-qPCR

A549-ACE2 cells were seeded into 96-well plates at 3 × 10^4^ cells per well 24 h before infection. BA.3.2.1 and LP.8.1 viruses were added at an MOI of 0.1, and cells were incubated at 4°C for 1 h to allow viral adsorption. After incubation, cells were washed three times with PBS to remove unbound virus and then lysed directly in TRIzol for total RNA extraction. Viral RNA levels were quantified by RT-qPCR using primers 2019-nCoV_N2-F and 2019-nCoV_N2-R targeting the viral N gene (Table S12). RT-qPCR was performed on the QuantStudio 7 Real-Time PCR instrument (Thermo Fisher Scientific). Relative RNA levels were obtained by normalizing the remaining viral RNA to input viral RNA using the 2^ΔCt^ method. Statistical significance was assessed using a two-tailed unpaired *t* test. Experiments were performed with three independent biological replicates.

### QUANTIFICATION AND STATISTICAL ANALYSIS

#### Statistics

Sample size estimation was based on previously published studies; no statistical methods were used to predefine sample sizes. All collected data were included in the analysis. The experiments were not randomized. Patient information was blinded, and investigators were blinded to sample identities during data collection and/or analysis. Neutralization assays were conducted in duplicate, and all replication attempts were successful. Continuous variables were reported as geometric means with 95% confidence intervals or as medians. For geometric mean titer (GMT) calculations and statistical comparisons, sera with undetectable antibody titers (<20) were assigned a value of 10. Comparison between neutralization titers was performed using the Wilcoxon matched-pairs signed-rank test with GraphPad Prism 9.

For western blot quantification, data are presented as means ± standard deviations from at least three independent experiments. Statistical significance was determined using one-way ANOVA with Dunnett’s multiple-comparisons test. For cell-cell fusion assay, data are presented as means ± standard deviations from at twelve independent experiments. Statistical significance was determined using one-way ANOVA with Dunnett’s multiple-comparisons test. For competition experiments, five independent infections were performed per group. Simple linear regression was used to assess the statistical significance of RNA ratios at each indicated time point relative to the input RNA ratio, with absolute *p*-values reported. A *p*-value of <0.05 was considered statistically significant. For viral growth kinetics experiments, four independent infections were conducted per group. Data were log10-transformed to approximate a normal distribution prior to statistical analysis. Data are presented as means ± standard deviations from four independent experiments. Statistical significance was determined using two-way ANOVA with Dunnett’s multiple comparison correction, with each variant compared with WA1. For virion attachment assay, data are presented as means ± standard deviations from at three independent experiments. Statistical significance was determined using one-way ANOVA with Dunnett’s multiple-comparisons test.

## Supplementary Material

1

2

3

4

5

7

8

9

10

11

12

13

14

Supplemental information can be found online at https://doi.org/10.1016/j.celrep.2026.117812.

## Figures and Tables

**Table:** KEY RESOURCES TABLE

REAGENT or RESOURCE	SOURCE	IDENTIFIER
Antibodies		
Rabbit polyclonal anti-SARS-CoV-2 spike S1	Sino Biology	Cat# 40591-T62
Rabbit monoclonal anti-SARS-CoV-2 N protein	Sino Biology	Cat# 40143-R001
Rabbit polyclonal anti-SARS-CoV-2 spike S2	Sino Biology	Cat# 40590-T62
Bacterial and virus strains		
*E. coli* strain Top10	Thermo Fisher Scientific	Cat# C404006
TransforMax EPI300 Chemically Competent *E. coli*	Thermo Fisher Scientific	Cat# C300C105
SARS-CoV-2 WA1 mNG	Kurhade et al.^10^	N/A
SARS-CoV-2 BA.3 mNG	Kurhade et al.^10^	N/A
SARS-CoV-2 BA.3.2.1 mNG	This study	N/A
SARS-CoV-2 KP.3 mNG	Hu et al.^40^	N/A
SARS-CoV-2 XEC mNG	This study	N/A
SARS-CoV-2 LP.8.1 mNG	This study	N/A
SARS-CoV-2 BA.3.2.1 N529Q mNG	This study	N/A
SARS-CoV-2 BA.3.2.1 K852A mNG	This study	N/A
Biological samples		
Human serum	This study	N/A
Chemicals, peptides, and recombinant proteins		
TRIzol LS Reagent	Invitrogen	Cat# 10296028
TRIzol Reagent	Invitrogen	Cat# 15596026
NHS-PEG4-Biotin	Thermo Fisher Scientific	Cat# 21330
Polyethylenimine Hydrochloride 40,000	Polysciences	Cat# 25322-68-3
SeaPlaque Agarose	Lonza	Cat# BMA50101
Laemmli Sample Buffer	Bio Rad	Cat# 1610737
β-mercaptoethanol	Sigma-Aldrich	Cat# M6250
Neutral Red Solution	Sigma-Aldrich	Cat# N2899
Dulbecco’s Modified Eagle Medium	Gibco	Cat# 11965092
FreeStyle 293 Expression Medium	Gibco	Cat# 12338018
Opti-MEM I Reduced Serum Medium	Gibco	Cat# 31985070
Critical commercial assays		
T7 mMessage mMachine kit	Thermo Fisher Scientific	Cat# AM1344
Ingenio^®^ Electroporation solution	Mirus Bio LLc	Cat# MIR50117
SuperScript IV One-Step RT-PCR System	Thermo Fisher Scientific	Cat# 12594100
iTaq SYBR Green One-Step Kit	Bio Rad	Cat# 1725151
CF200-Cu carbon film grid	Electron Microscopy Sciences	Cat# CF200-Cu-50
Quantifoil R1.2/1.3 Cu, 300 mesh	Electron Microscopy Sciences	Cat# Q350CR1.3
SA sensor	Sartorius	Cat# 18-5019
Deposited data		
Closed BA.3.2.1 spike	This study	PDB: 9YSJ; EMD: 73393
Local refinement of RBD_C_/RBD_A_/NTD_B_, closed BA.3.2.1 spike	This study	PDB: 9YX8; EMD: 73599
Local refinement of RBD_A_/NTD_C_, closed BA.3.2.1 spike	This study	PDB: 9YX7; EMD: 73597
Local refinement of RBD_B_/NTD_A_, closed BA.3.2.1 spike	This study	PDB: 9YXX; EMD: 73619
Local refinement of NTD_B_, closed BA.3.2.1 spike	This study	PDB: 9Z3Y; EMD: 73795
Flexible BA.3.2.1 spike	This study	PDB: 9YX6; EMD: 73395
hACE2/BA.3.2.1 spike complex conformation 1	This study	EMD: 73404
hACE2/BA.3.2.1 spike complex conformation 2	This study	EMD: 73405
hACE2/BA.3.2.1 spike complex conformation 3	This study	EMD: 73408
hACE2/BA.3.2.1 spike complex conformation 4	This study	EMD: 73409
Local refinement of hACE2/BA.3.2.1 RBD	This study	PDB: 9YSR; EMD: 73426
Closed LP.8.1 spike	This study	PDB: 9YSK; EMD: 73394
Local refinement of RBD_A_/NTD_C_, closed LP.8.1 spike	This study	PDB: 9YT4; EMD: 73441
Local refinement of RBD_B_/NTD_A_, closed LP.8.1 spike	This study	PDB: 9YT6; EMD: 73443
Local refinement of RBD_C_/NTD_B_, closed LP.8.1 spike	This study	PDB: 9YT7; EMD: 73444
Open LP.8.1 spike	This study	PDB: 9YW0; EMD: 73535
Flexible LP.8.1 spike	This study	EMD: 73396
hACE2/LP.8.1 spike complex conformation 1	This study	EMD: 73427
hACE2/LP.8.1 spike complex conformation 2	This study	EMD: 73428
hACE2/LP.8.1 spike complex conformation 3	This study	EMD: 73430
hACE2/LP.8.1 spike complex conformation 4	This study	EMD: 73439
local refinement of hACE2/LP.8.1 RBD	This study	PDB: 9YSS; EMD: 73429
Closed BA.3.2.1 spike with K852A mutation	This study	PDB: 12JT; EMD: 76493
Local refinement of RBD_C_/RBD_A_/NTD_B_, closed BA.3.2.1 spike with K852A mutation	This study	PDB: 13BD; EMD: 76936
Open BA.3.2.1 spike with K852A mutation	This study	EMD: 76850
Flexible BA.3.2.1 spike with K852A mutation	This study	EMD: 76706
Closed BA.3.2.1 spike with N529Q mutation	This study	PDB: 12JZ; EMD: 76501
Local refinement of RBD_C_/NTD_B_, closed BA.3.2.1 spike with N529Q mutation	This study	PDB: 12RP; EMD: 76713
Open BA.3.2.1 spike with N529Q mutation	This study	EMD: 76839
Flexible BA.3.2.1 spike with N529Q mutation	This study	EMD: 76694
Experimental models: Cell lines		
Vero E6	ATCC	CRL-1586
Vero E6-TMPRSS2	SEKISUI XenoTech	JCRB1819
Calu-3	ATCC	HTB-55
HEK293T	ATCC	CRL-3216
HEK293T-ACE2	This study	N/A
Freestyle 293-F	Thermo Fisher Scientific	Cat# R79007
Human primary airway epithelial (HAE)	MatTek Life Sciences	Cat# AIR-100
Experimental models: Organisms/strains		
Omicron variant sublineage XEC	GISAID: EPI_ISL_19283891	N/A
Omicron variant sublineage LP.8.1	GISAID: EPI_ISL_19674199	N/A
Omicron variant sublineage BA.3.2.1	GISAID: EPI_ISL_19771108	N/A
Oligonucleotides		
Primer: CoV-21115V	Integrated DNA Technologies	N/A
Primer: CoV-YH5	Integrated DNA Technologies	N/A
Primer: K529N-F	Integrated DNA Technologies	N/A
Primer: K529N-R	Integrated DNA Technologies	N/A
Primer: A852K-F	Integrated DNA Technologies	N/A
Primer: A852K-R	Integrated DNA Technologies	N/A
Primer: K1086R-F	Integrated DNA Technologies	N/A
Primer: K1086R-R	Integrated DNA Technologies	N/A
Primer: F59S-F	Integrated DNA Technologies	N/A
Primer: F59S-R	Integrated DNA Technologies	N/A
Primer: N529Q-F	Integrated DNA Technologies	N/A
Primer: N529Q-R	Integrated DNA Technologies	N/A
Primer: 2019-nCoV_N2-F	Integrated DNA Technologies	N/A
Primer: 2019-nCoV_N2-R	Integrated DNA Technologies	N/A
Primer: K852A-F	Integrated DNA Technologies	N/A
Primer: K852A-R	Integrated DNA Technologies	N/A
Recombinant DNA		
pαH-GSAS-6P-BA.3.2.1-spike ectodomain	This study	N/A
pαH-GSAS-6P-BA.3.2.1-spike ectodomain-N529Q	This study	N/A
pαH-GSAS-6P-BA.3.2.1-spike ectodomain-K852A	This study	N/A
pαH-GSAS-6P-LP.8.1-spike ectodomain	This study	N/A
pCAG-SARS-CoV-2 WA1 RBD	This study	N/A
pCAG-SARS-CoV-2 BA.3 RBD	This study	N/A
pCAG-SARS-CoV-2 KP.3 RBD	This study	N/A
pCAG-SARS-CoV-2 XEC RBD	This study	N/A
pCAG-SARS-CoV-2 LP.8.1 RBD	This study	N/A
pCAG-SARS-CoV-2 BA.3.2.1 RBD	This study	N/A
pCAG-Human ACE2 1-615	Yurkovetskiy et al.^84^	Addgene #158089
pXJ-SARS-CoV-2 WA.1 Spike	This study	N/A
pXJ-SARS-CoV-2 BA.3 Spike	This study	N/A
pXJ-SARS-CoV-2 BA.3.2.1 Spike	This study	N/A
pXJ-SARS-CoV-2 BA.3.2.1 N529Q Spike	This study	N/A
pXJ-SARS-CoV-2 BA.3.2.1 K852A Spike	This study	N/A
pXJ-SARS-CoV-2 LP.8.1 Spike	This study	N/A
pXJ-eGFP	Xie et al.^85^	N/A
Software and algorithms		
UCSF ChimeraX 1.10.1	Pettersen et al.^86^	PMID: 32881101
UCSF Chimera 1.17.2	Pettersen et al.^87^	PMID: 37774136
Coot 0.9.8.1	Emsley et al.^88^	PMID: 15572765
Phenix 1.21.2	Adams et al.^89^	PMID: 20124702
VMD 1.9.4a57	Humphrey et al.^90^	https://www.ks.uiuc.edu/
CryoSPARC 4.7.1	Punjani et al.^91^	PMID: 28165473
ImageJ	Schinder et al.^92^	https://imagej.net/ij/
GraphPad Prism 9	GraphPad	https://www.graphpad.com/
Illustrator CC	Adobe	N/A
Octet Data Analysis software V13	Sartorius	N/A
Other		
Vitrobot Mark IV	Thermo Fisher Scientific	N/A
Titan Krios G3i	Thermo Fisher Scientific	N/A
Titan Krios G4	Thermo Fisher Scientific	N/A
HPLC system-AKTA Pure	GE Healthcare	N/A
Superdex 200 10/300 GL column	Cytiva	Cat# 17517501
Superose 6 10/300 GL column	Cytiva	Cat# 29091596
His-tag Purification Resin	Qiagen	Cat# 30210
Octet R8	Sartorius	N/A

## Highlights

- BA.3.2.1 spike confers strong resistance to neutralization by KP.2/KP.3 sera
- BA.3.2.1 shows reduced replication in airway cultures versus JN.1 descendants
- Increased RBD affinity does not enhance hACE2 engagement by BA.3.2.1 spike
- N529 glycan and K852 stabilize a closed asymmetric spike, restricting BA.3.2.1 replication
