# Supplementary material for: Functional and structural basis of Omicron BA.3.2.1 spike
Source: Cell Rep. Author manuscript; Available in PMC 2026 Sep 26. (PMC13615522; doi:10.1016/j.celrep.2026.117812)
Supplement: Supplementary file 1 [file NIHMS2206921-supplement-1.pdf]

Cell Reports, Volume *45*

Supplemental information

Functional and structural basis of Omicron BA.3.2.1 spike

Yan Wang, Yanping Hu, Zhenhang Chen, Jing Zou, Ke Zhang, Ping Ren, Pei-Yong Shi, Bo Liang, and Xuping Xie

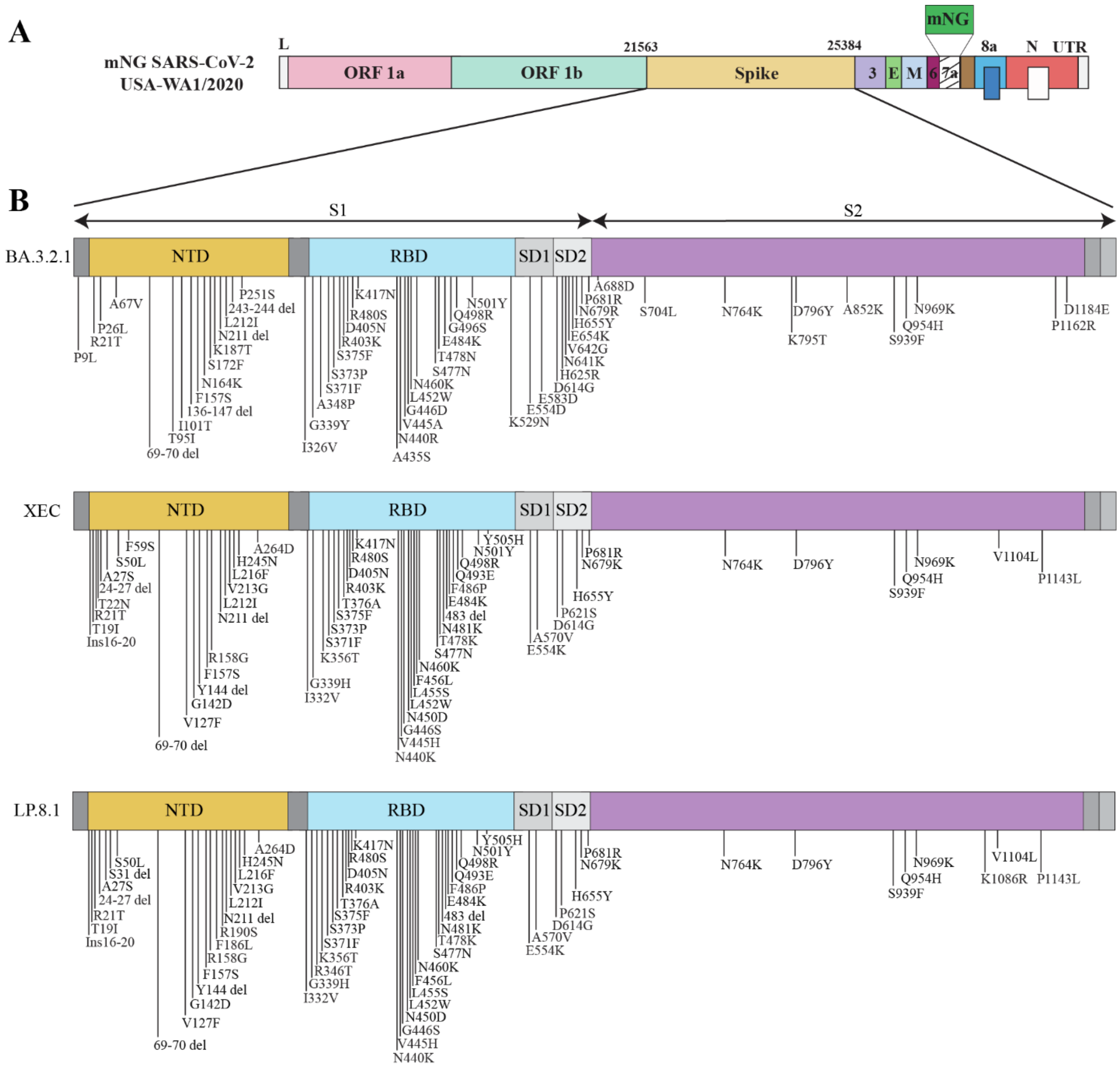

Supplemental Figure S1. Schematic of mNG SARS-CoV-2 spike-variant construction.

(A) The mNG SARS-CoV-2 derived from strain USA-WA1/2020 (WA1) was used as the backbone. The WA1 spike ORF was replaced with the spike gene from each variant to generate the corresponding variant-spike mNG SARS-CoV-2. L, leader sequence; ORF, open reading frame; E, envelope protein; M, membrane protein; N, nucleocapsid; UTR, untranslated region; mNG, mNeonGreen.

(B) Spike mutations across BA.3.2.1, XEC, and LP.8.1. Amino acid substitutions in each spike variant relative to WA1 are annotated along the domain map. NTD: N-terminal domain of spike; RBD: receptor binding domain; S1: N-terminal furin-cleavage fragment of S; SD: subdomain; S2: C-terminal furin-cleavage fragment of S.

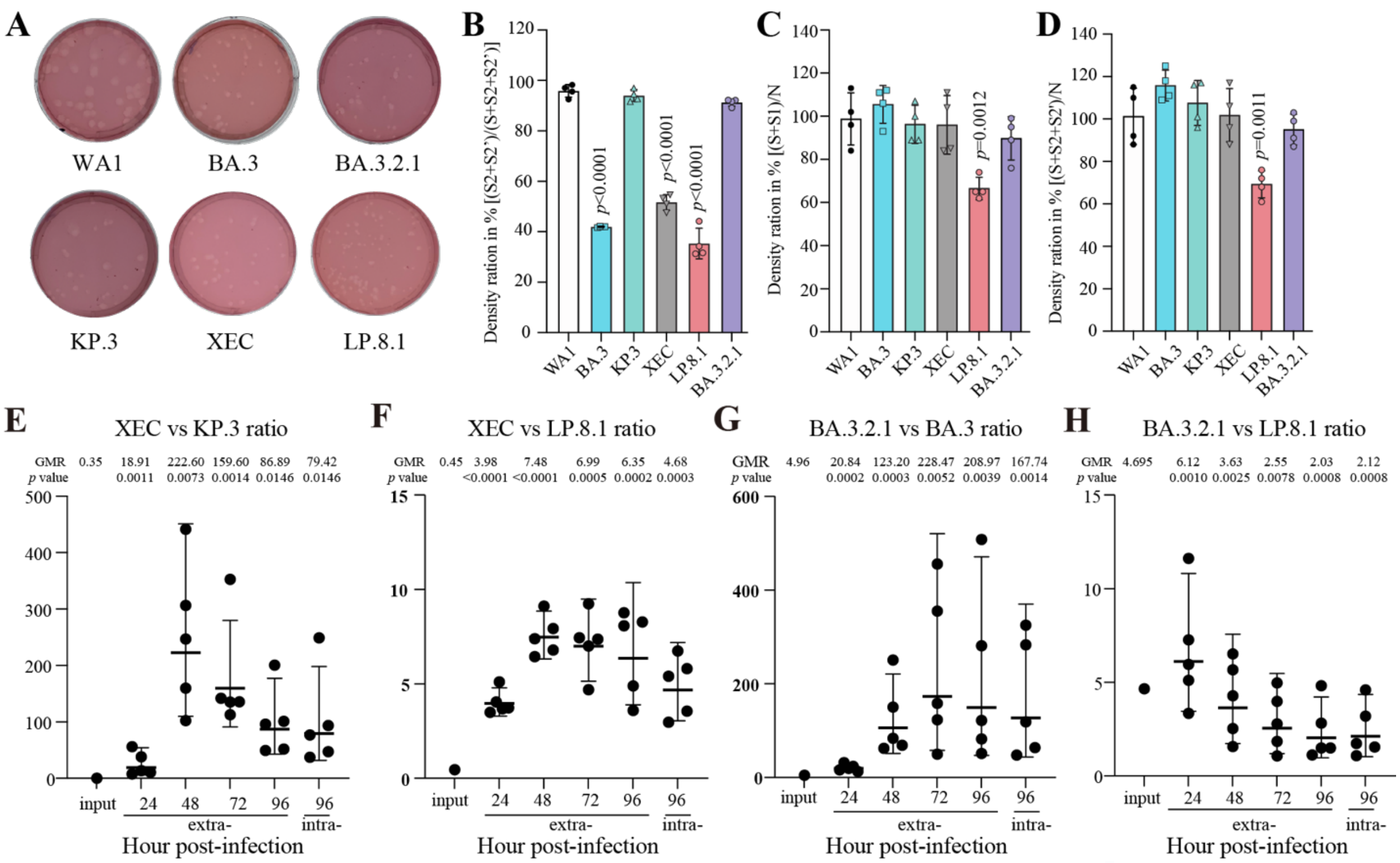

Supplemental Figure S2. Characterization of mNG SARS-CoV-2 spike variants.

(A) Representative plaque morphologies of mNG SARS-CoV-2 variants on Vero E6-TMPRSS2 cells.

(B) Ratio of S2 plus S2' to total spike (S+S2+S2') in virions.

(C-D) Ratio of total spike to N in virions quantified by Western blot using anti-S1 (C) or anti-S2 (D) antibody. Data in (B)-(D) are represented as mean ± SD. *$p < 0.05$; **$p < 0.01$; ****$p < 0.0001$, one-way ANOVA with multiple-comparison corrections.

(E-H) Scatter plot of RNA ratios measured in infected HAE: XEC: KP.3 (E), XEC: LP.8.1 (F), BA.3.2.1: BA.3 (G), and BA.3.2.1: LP.8.1 (H). Geometric mean ratios (GMRs) and 95% confidence intervals (CIs) are shown. *p* values were calculated from two-sided linear regression analyses comparing the observed RNA ratio at each time point to the input RNA ratio.

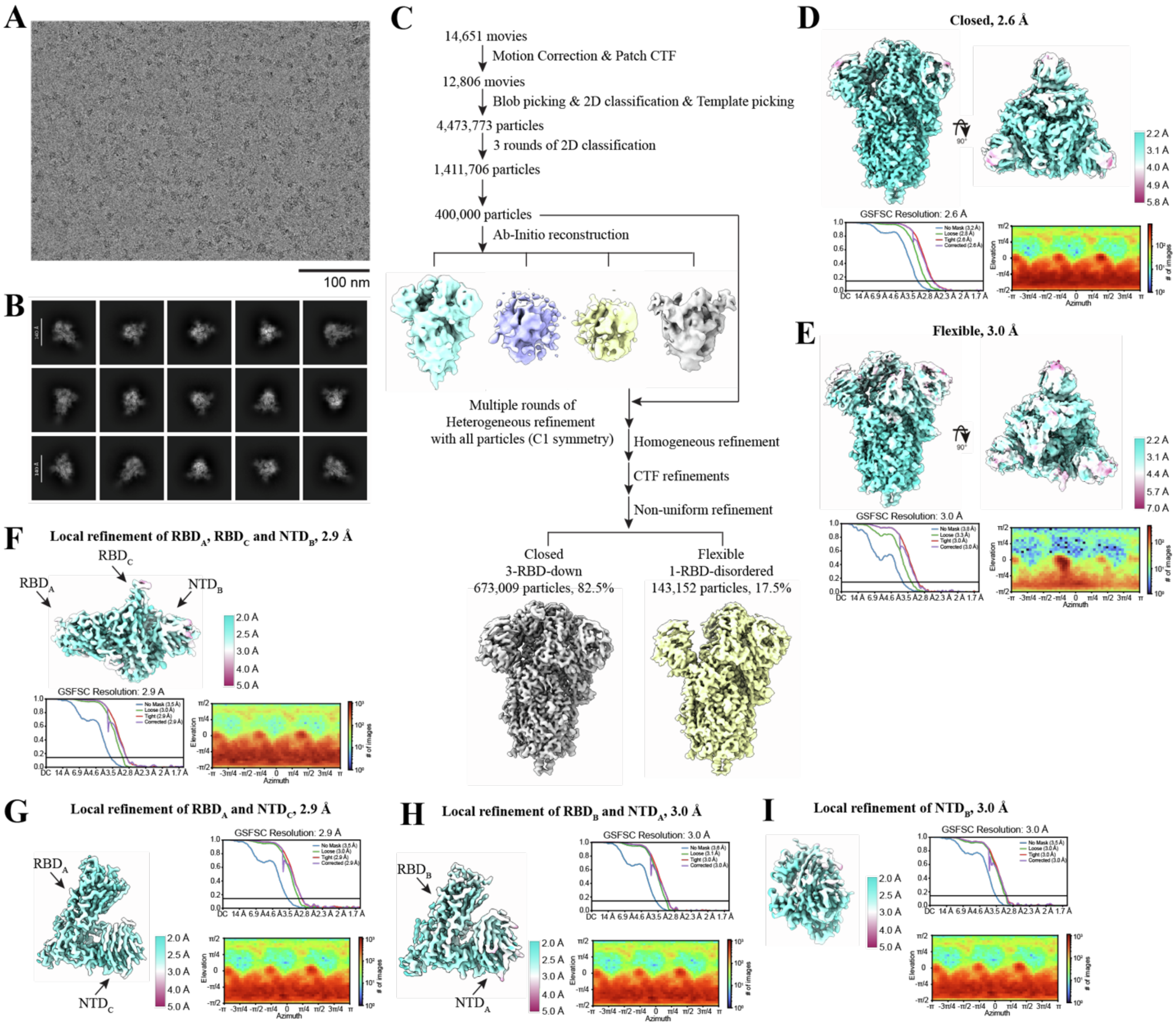

Supplemental Figure S3. Cryo-EM processing workflow for the BA.3.2.1 spike.

(A) Representative micrograph of the BA.3.2.1 spike. Scale bar, 100 nm.

(B) Representative 2D class averages of selected particles.

(C) Data processing flowchart.

(D-E) Global map quality for the non-uniform refinement maps of the closed (**D**) and flexible (**E**) conformations, showing local-resolution distributions, Fourier shell correlation (FSC) curves, and particle angular distributions.

(F-I) Local refinement of the RBD_A, RBD_C, and NTD_B **(F)**, RBD_A and NTD_C **(G)**, RBD_B and NTD_A (H), and NTD_B (**I**) in closed conformation, with corresponding local-resolution distributions, FSC curves, and angular distributions.

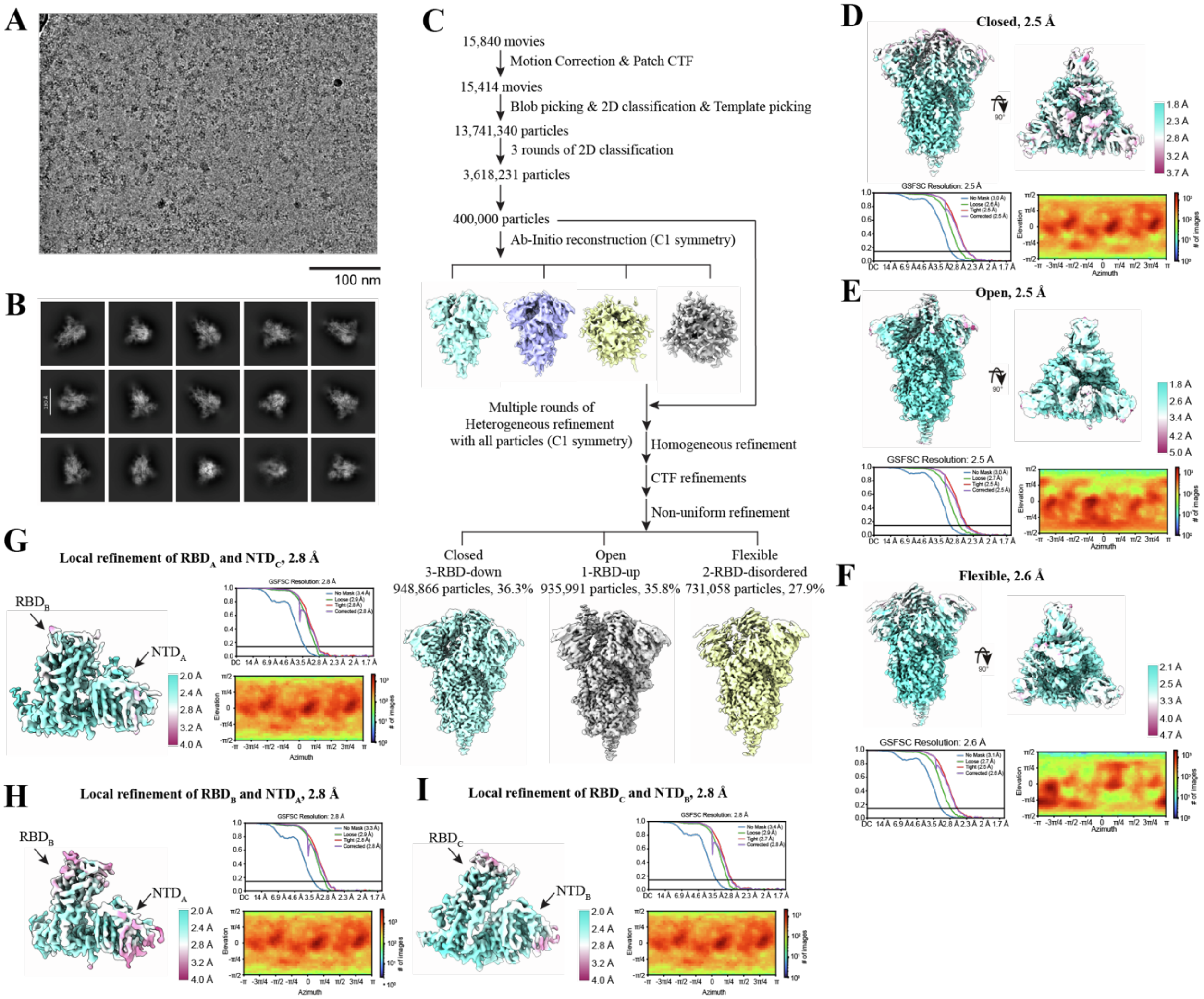

Supplemental Figure S4. Cryo-EM processing workflow for the LP.8.1 spike.

(A) Representative micrograph of the LP.8.1 spike. Scale bar, 100 nm.

(B) Representative 2D class averages of selected particles.

(C) Data processing flowchart.

(D-F) Global map quality for the non-uniform refinement maps of LP.8.1 in the closed (D), open (E), and flexible (F) conformations, showing local-resolution distributions, FSC curves, and particle angular distributions.

(G-I) Local refinements for the closed conformation, with corresponding local-resolution distributions, FSC curves, and angular distributions for: RBD_A and NTD_C (G), RBD_B and NTD_A (H), RBD_C and NTD_B (I).

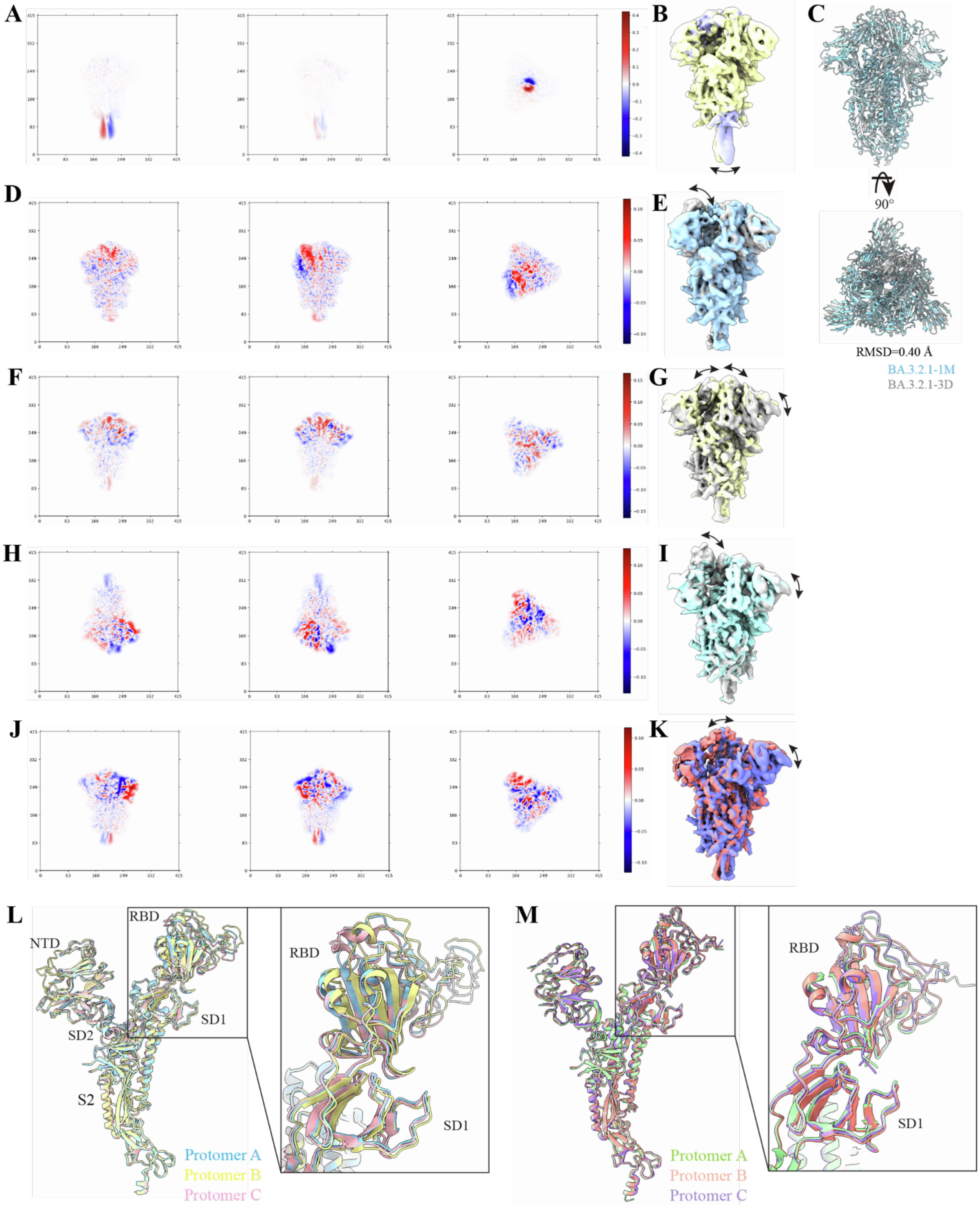

Supplemental Figure S5. Structural analysis of BA.3.2.1 and LP.8.1 spikes.

(A) Scatter plots showing the dispersion of particle latent coordinates throughout the closed BA.3.2.1 spike ensemble.

(B) Representative cryo-EM maps of the closed BA.3.2.1 spike after 3D flexible-refinement training.

(C) Structural alignment of the BA.3.2.1 spike in the closed (gray) and flexible (blue) conformations.

(D) Scatter plots showing the dispersion of particle latent coordinates throughout the flexible BA.3.2.1 spike ensemble.
(E) Representative cryo-EM maps of the flexible BA.3.2.1 spike after 3D flexible-refinement training.
(F) Scatter plots showing the dispersion of particle latent coordinates throughout the closed LP.8.1 spike ensemble.
(G) Representative cryo-EM maps of the closed LP.8.1 spike after 3D flexible-refinement training.
(H) Scatter plots showing the dispersion of particle latent coordinates throughout the open LP.8.1 spike ensemble.
(I) Representative cryo-EM maps of the open LP.8.1 spike after 3D flexible-refinement training.
(J) Scatter plots showing the dispersion of particle latent coordinates throughout the flexible LP.8.1 spike ensemble.
(K) Representative cryo-EM maps of the flexible LP.8.1 spike after 3D flexible-refinement training.
(L-M) Superposition of the main-chain protomers of the BA.3.2.1 spike (L) or LP.8.1 spike (M) in the closed conformations, aligned using S2 residues 908-1035 of the HR1-CH region. Right panels: close-up views of the RBD and SD1 regions.

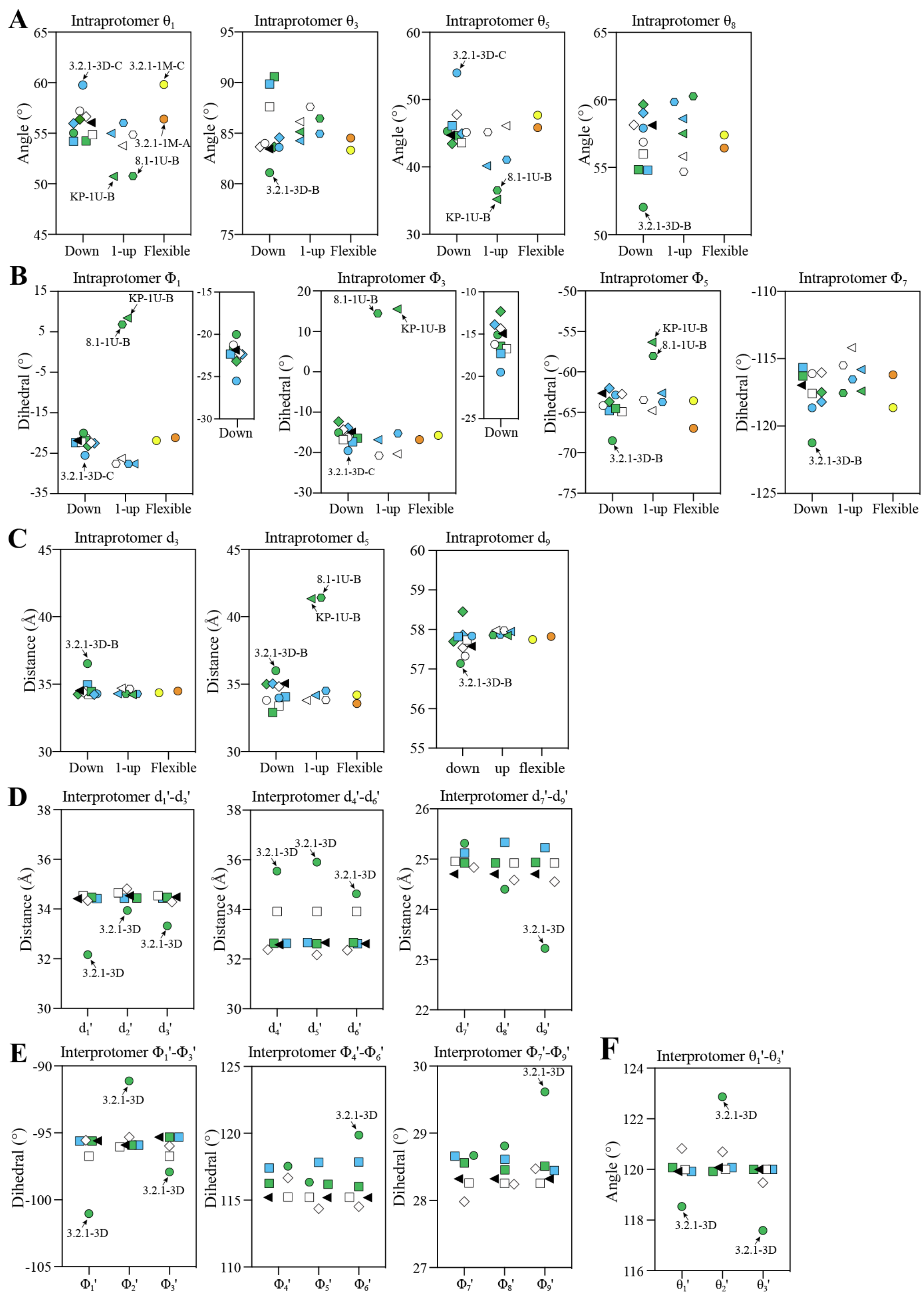

Supplemental Figure S6. Vector analysis of the BA.3.2.1 spike.

(A) Intraprotomer angles θ_1, θ_3, θ_5, and θ_8 describing angular motion of SD2 relative to RBD about SD1, NTD' relative to SD2 about SD1, SD1 relative to RBD about RBDa, and CD relative to SD2 about SD1, respectively.

(B) Intraprotomer Φ_1, Φ_3, Φ_5, Φ_7 dihedral angles describing rotation of the SD2 and RBDa about SD1 to RBD axis, NTD' and RBD about SD2 to SD1 axis, the NTD and SD1 about SD2 to NTD′ axis, and the SD1 and S2s about SD2 to CD axis, respectively, within the same protomer.

(C) Intraprotomer d_3, d_5, and d_9 describing distances between SD2-SD1, SD1-RBD, and CD-SD2, respectively, within the same protomer.
(D) Interprotomer d_1'-d_3', d_4'-d_6', and d_7'-d_9' describing the distances between RBDs, RBD-NTD, and SD1-NTD measured across protomer pairs A-C, C-B, and B-A, respectively.
(E) Interprotomer Φ_1'-Φ_3', Φ_4'-Φ_6', and Φ_7'-Φ_9' dihedral angles describing rotation between two RBDa from neighboring protomers about the RBD-RBD axis, SD1a-NTD'a from neighboring protomers about the SD1-NTD' axis, and SD2a from neighboring protomers about the SD1-NTD' axis, respectively.
(F) Interprotomer θ_1'-θ_3' angles formed by RBD_A-RBD_C-RBD_B (θ_1'), RBD_C-RBD_B-RBD_A (θ_2'), and RBD_B-RBD_A-RBD_C (θ_3').

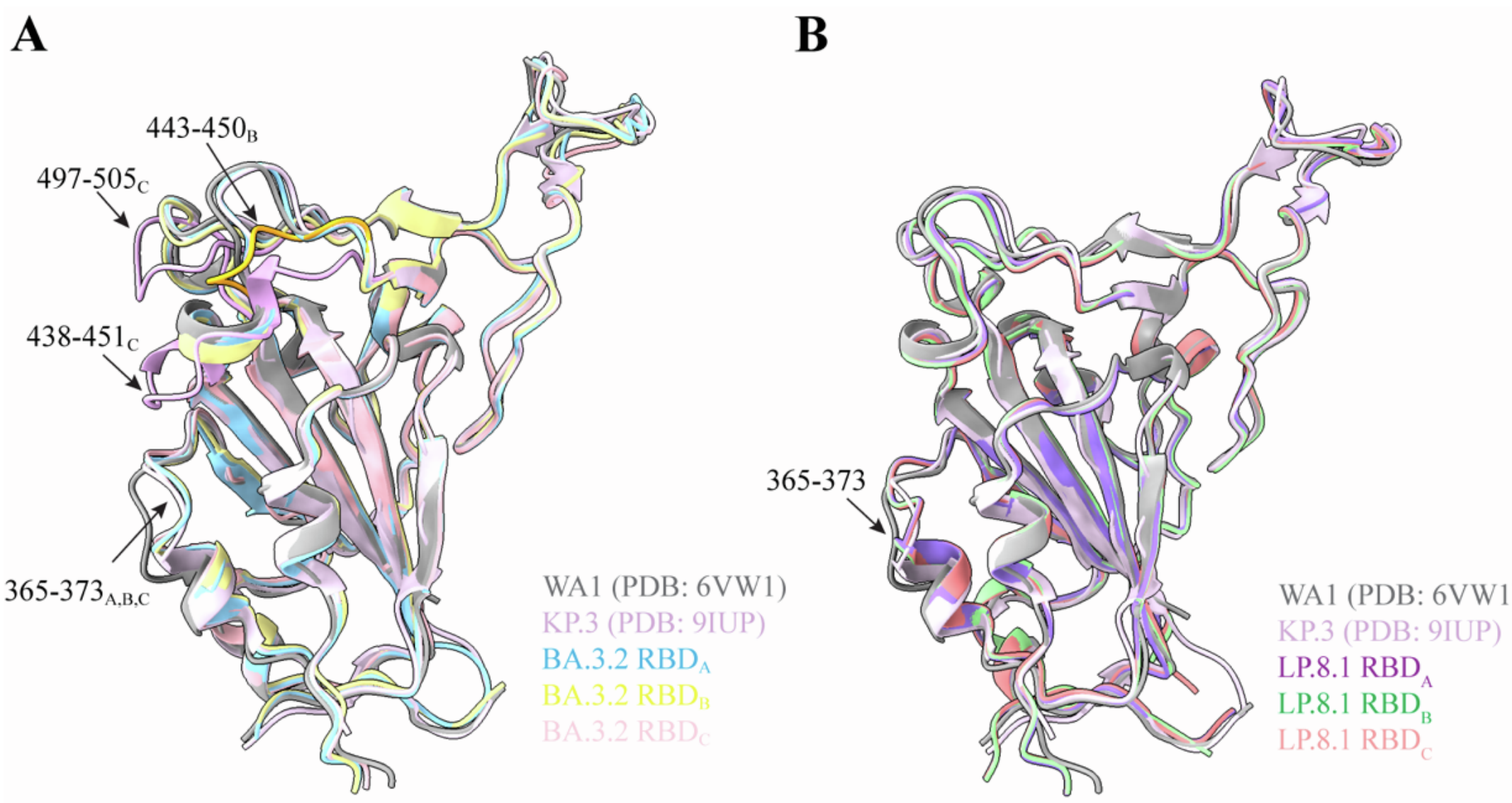

Supplemental Figure S7. Structural rearrangements of the BA.3.2.1 RBD.
(A) Structural alignment of the BA.3.2.1 (closed; 3D) RBDs from the three protomers with WA1 and KP.3 RBDs. An arrow and colored orange indicate the 443-450 loop in protomer B of closed BA.3.2.1; the 438-451 and 497-505 loops in protomer C of closed BA.3.2.1 are indicated by arrows and colored plum.
(B) Structural alignment of the LP.8.1 (closed; 3D) RBDs from the three protomers with WA1 and KP.3 RBDs. The loop 365-373 in (A-B) is annotated.

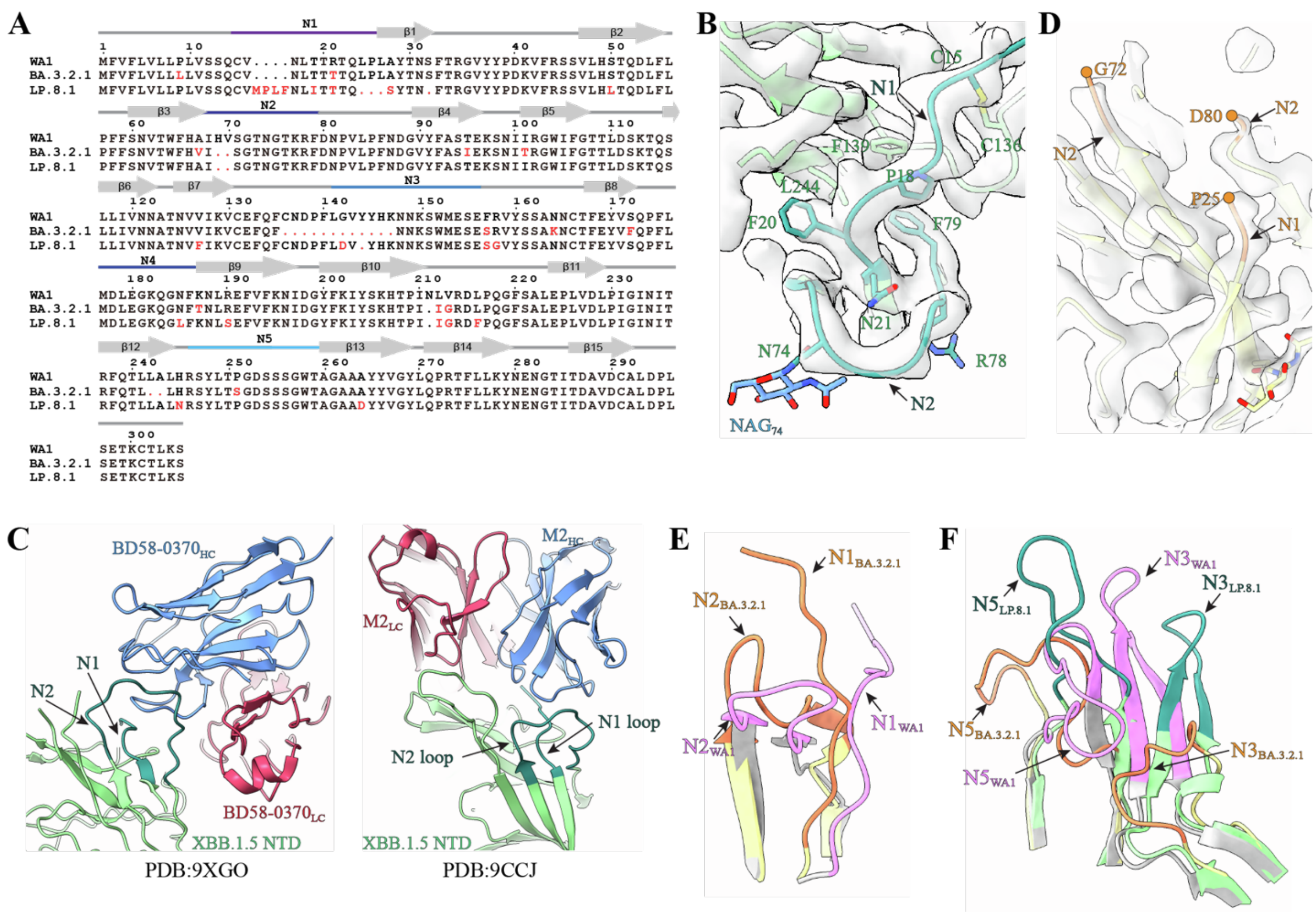

Supplemental Figure S8. Structural rearrangements of the BA.3.2.1 NTD.
(A) Secondary-structure maps and sequence alignment of WA1, BA.3.2.1, and LP.8.1 NTDs. Mutations are highlighted in red.
(B) Close-up views of the interaction network stabilizing the thread-through arrangement of N1 and N2 loops in the LP.8.1 NTD, with the cryo-EM density shown in gray.
(C) N1-N2 loop crossing in XBB.1.5 NTD stabilized by nAbs BD58-0370 (left) or M2 (right).
(D) Close-up views of the N1-N2 loop region in the BA.3.2.1 NTD, with the cryo-EM density shown in gray.
(E) Zoomed-in superposition of N1 and N2 loops from WA1 NTD (plum, PDB 7B62) and BA.3.2.1 NTD (orange).
(F) Zoomed-in superposition of N3 and N5 loops from WA1 NTD (plum), BA.3.2.1 NTD (orange), and LP.8.1 NTD (teal).

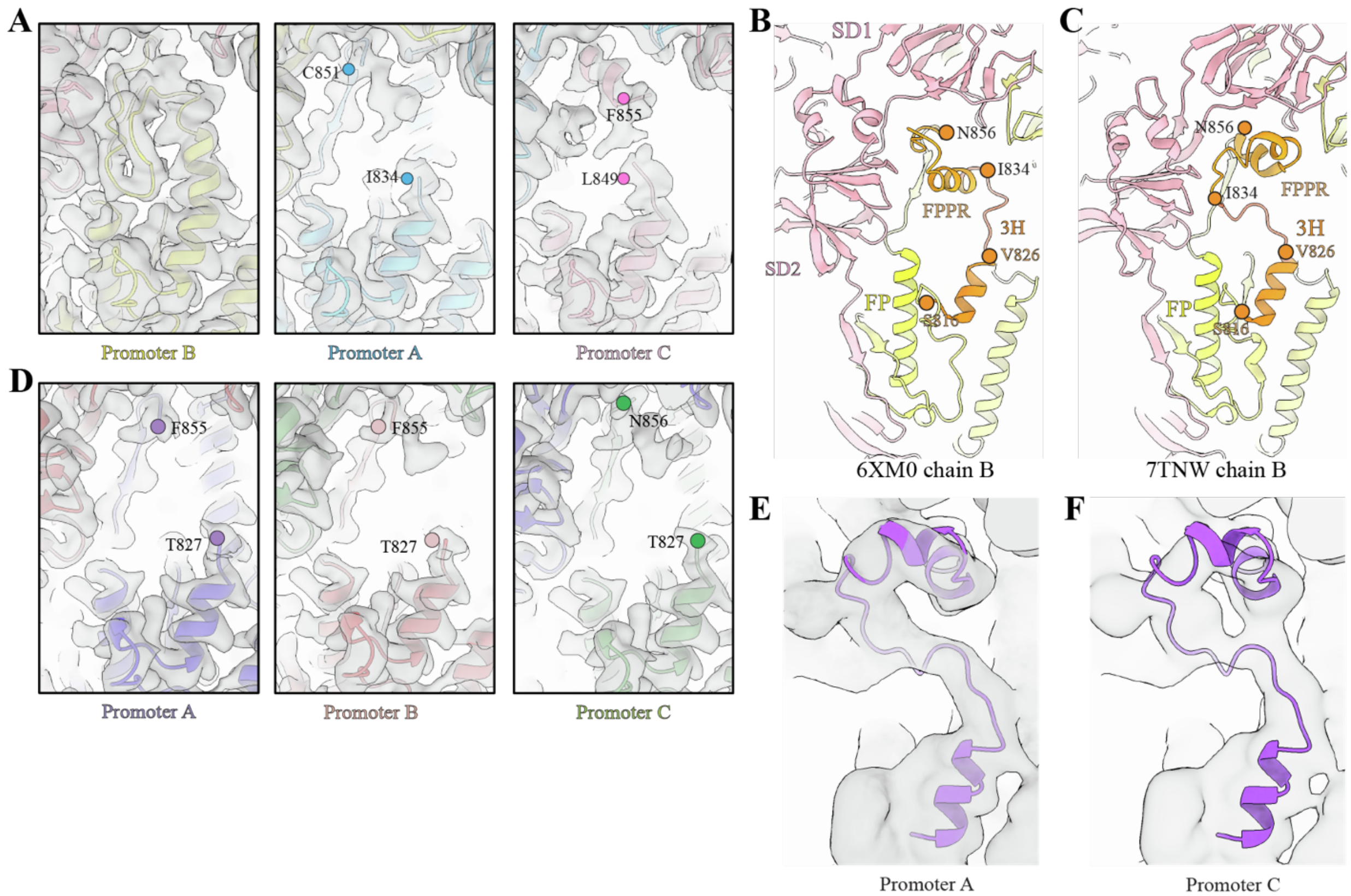

Supplemental Figure S9. Structures of FPPRs.
(A) Close-up views of the FPPR region within three protomers of the closed BA.3.2.1 spike with cryo-EM density in gray.
(B-C) The FPPR (orange) and FP (yellow) in WA1 (B) and Omicron B.1.1.529 (C) spike structures.
(D) Close-up views of the FPPR region within three protomers of the closed LP.8.1 spike with cryo-EM density in gray.
(E-F) Fitting of the 3H and loop-helix-loop FPPR from Omicron B.1.1.529 (PDB: 7TNW) into low-resolution cryo-EM maps of protomers A (E) and C (F) of the closed BA.3.2.1 spike, generated by 3DVA.

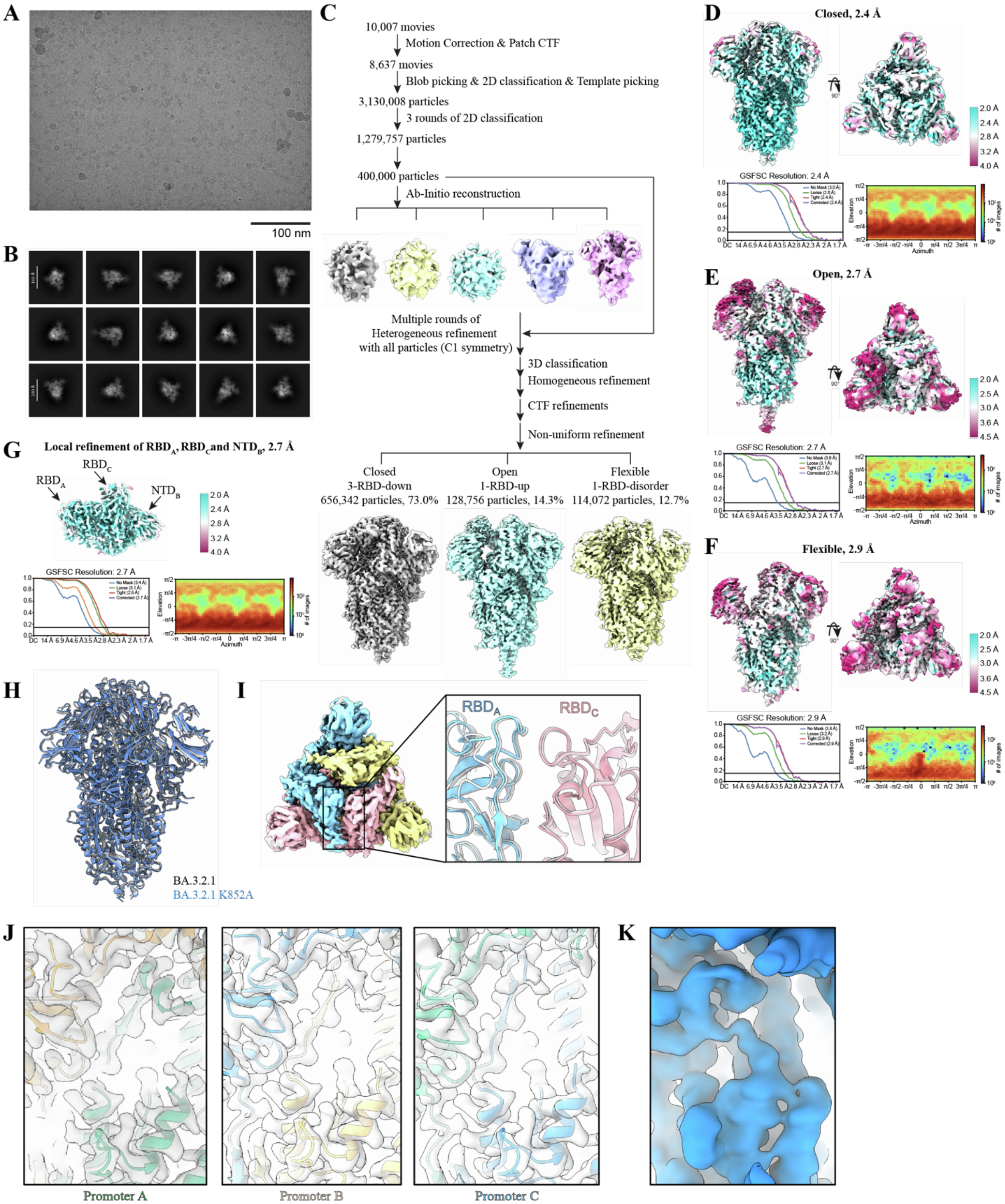

Supplemental Figure S10. Cryo-EM processing workflow and analysis for BA.3.2.1 K852A spike.
(A) Representative micrograph. Scale bar, 100 nm.
(B) Representative 2D class averages of selected particles.
(C) Data processing flowchart.
(D-F) Global map quality for the non-uniform refinement maps of BA.3.2.1 K852A spike in the closed (D), open (E), and flexible (F) conformations, showing local-resolution distributions, FSC curves, and particle angular distributions.

(G) Local refinements for the RBD_A, RBD_C, and NTD_B in closed conformation, with corresponding local-resolution distributions, FSC curves, and angular distributions.
(H) Structural alignment of the closed WT (gray) and K852A (blue) BA.3.2.1 spikes.
(I) Top view of the closed BA.3.2.1 K852A spike cryo-EM map, highlighting the inter-RBD interaction between RBD_A and RBD_C (black box). The enlarged view shows the K852A spike structure aligned with the WT BA.3.2.1 structure (gray).
(J) Close-up view of the FPPR region within three protomers of the closed BA.3.2.1 K852A spike with cryo-EM density in gray.
(K) Low-resolution cryo-EM map of the FPPR region in protomer B of the closed BA.3.2.1 K852A spike, generated by 3D variability analysis.

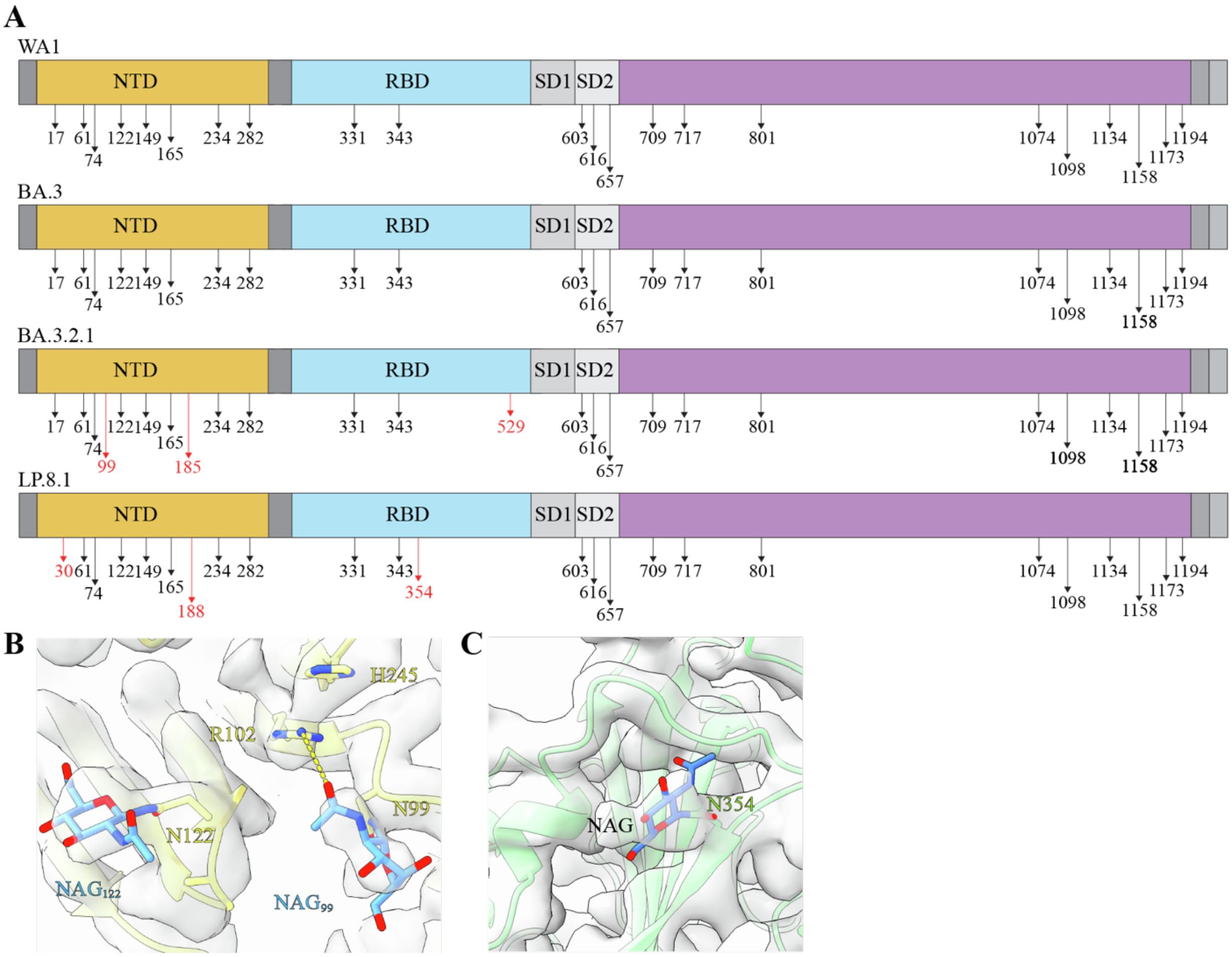

Supplemental Figure S11. N-linked glycans on BA.3.2.1 and LP.8.1 spikes.

(A) Schematic map of *N*-linked glycosylation sites on the BA.3.2.1 and LP.8.1 spikes. Sites that differ from WA1 are highlighted in red.

(B) Cryo-EM map surrounding the N529 glycan in protomer B of the BA.3.2.1 spike, with the cryo-EM density shown in gray.

(C) Close-up view of the N354 glycan on the LP.8.1 spike, with the cryo-EM density shown in gray.

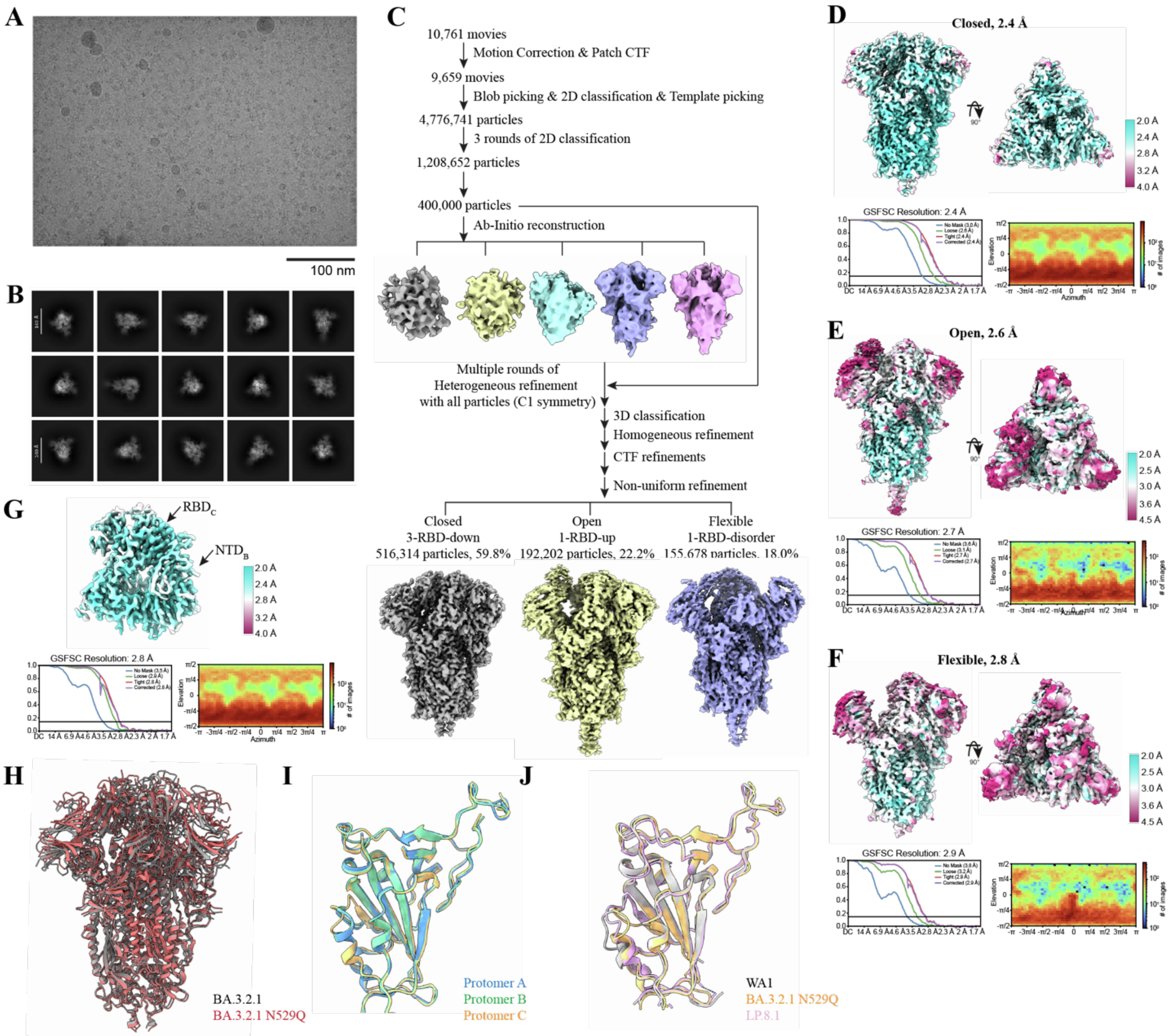

Supplemental Figure S12. Cryo-EM processing workflow of BA.3.2.1 N529Q spike.

(A) Representative micrograph. Scale bar, 100 nm.

(B) Representative 2D class averages of selected particles.

(C) Data processing flowchart.

(D-F) Global map quality for the non-uniform refinement maps of BA.3.2.1 spike with N529Q mutation in the closed (D), open (E), and flexible (F) conformations, showing local-resolution distributions, FSC curves, and particle angular distributions.

(G) Local refinements for the RBD_C and NTD_B in closed conformation, with corresponding local-resolution distributions, FSC curves, and angular distributions.

(H) Structural alignment of the closed WT (gray) and N529Q (red) BA.3.2.1 spikes.

(I) Alignment of three RBDs from the full-length model of closed BA.3.2.1 N529Q spike.

(J) Alignment of WA1 RBD, LP.8.1 RBD, and BA.3.2.1 N529Q RBD from protomer C.

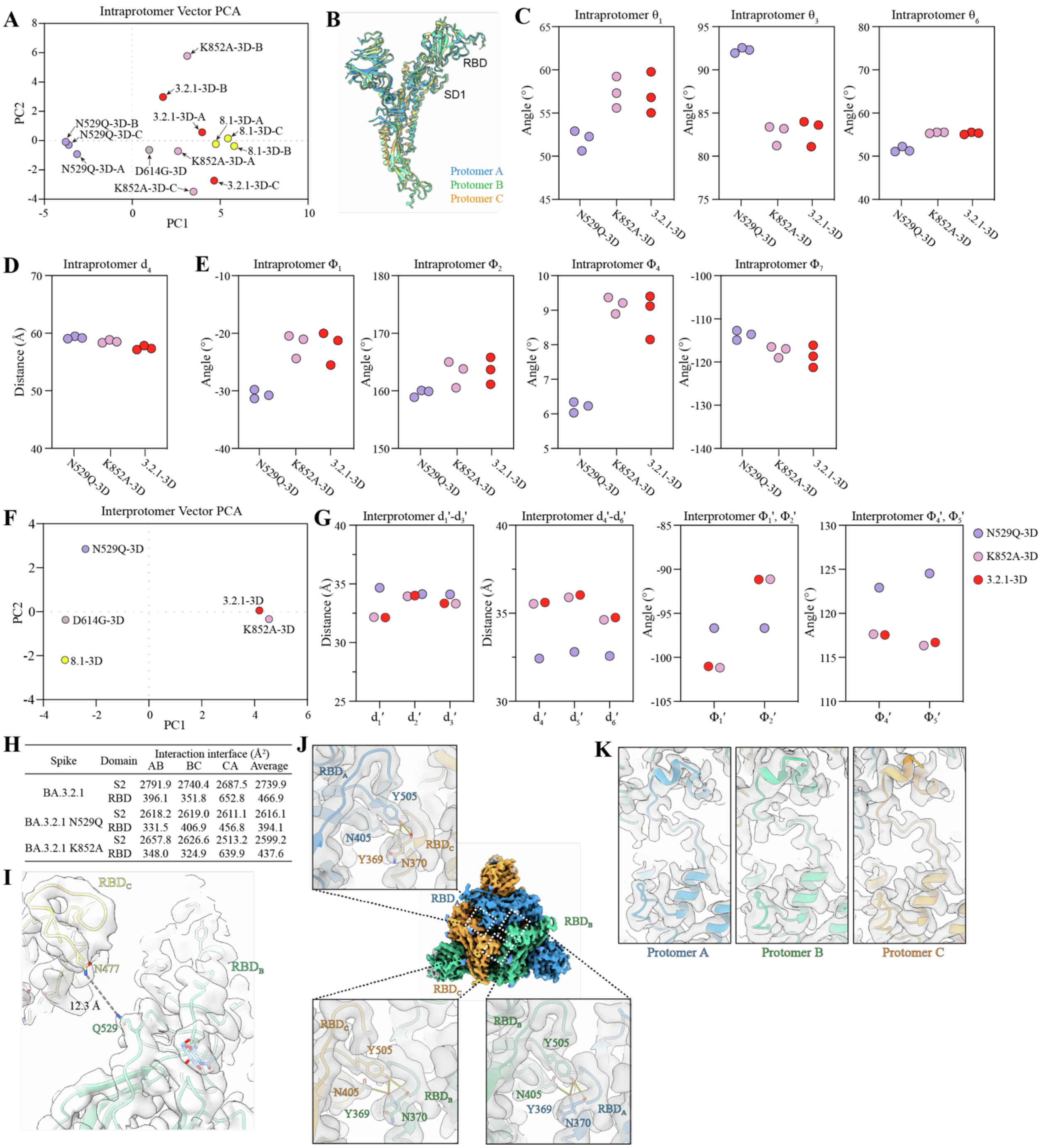

Spike	Domain	Interaction interface (Å²) AB	BC	CA	Average
BA.3.2.1	S2	2791.9	2740.4	2687.5	2739.9
	RBD	396.1	351.8	652.8	466.9
BA.3.2.1 N529Q	S2	2618.2	2619.0	2611.1	2616.1
	RBD	331.5	406.9	456.8	394.1
BA.3.2.1 K852A	S2	2657.8	2626.6	2513.2	2599.2
	RBD	348.0	324.9	639.9	437.6

Supplemental Figure S13. Structural analysis of BA.3.2.1 N529Q spike

(A) PCA of intraprotomer vector magnitudes, angles, and dihedrals of closed BA.3.2.1 N529Q and K852A spikes, with closed D614G, LP.8.1, BA.3.2.1 spikes shown as reference.

(B) Superposition of main-chain protomers from the closed BA.3.2.1 N529Q spike.

(C-E) Intraprotomer angles θ_1, θ_3, θ_6 (C), distance d_4 (D), and dihedral angles Φ_1, Φ_2, Φ_4 and Φ_7 (E) in the three protomers of closed N529Q, K852A, and WT BA.3.2.1 spikes.

(F) PCA of interprotomer vector magnitudes, angles, and dihedrals for closed BA.3.2.1 N529Q and K852A spikes, with closed D614G, LP.8.1, BA.3.2.1 spikes shown as reference.

(G) Interprotomer distances d_1'-d_6' and dihedral angles Φ_1', Φ_2', Φ_4', and Φ_5' in closed N529Q, K852A, and WT BA.3.2.1 spikes.

(H) Binding surface areas between neighboring S2 subunits and RBDs in closed N529Q, K852A, and WT BA.3.2.1 spikes.
(I) Magnified view of Q529 in protomer B of the closed BA.3.2.1 N529Q spike, with cryo-EM density shown in gray.
(J) Top view of the N529Q spike cryo-EM maps, with zoomed-in views showing reorganized inter-RBD interactions.
(K) Close-up view of the FPPR region within three protomers of the closed BA.3.2.1 N529Q spike, with cryo-EM density shown in gray.

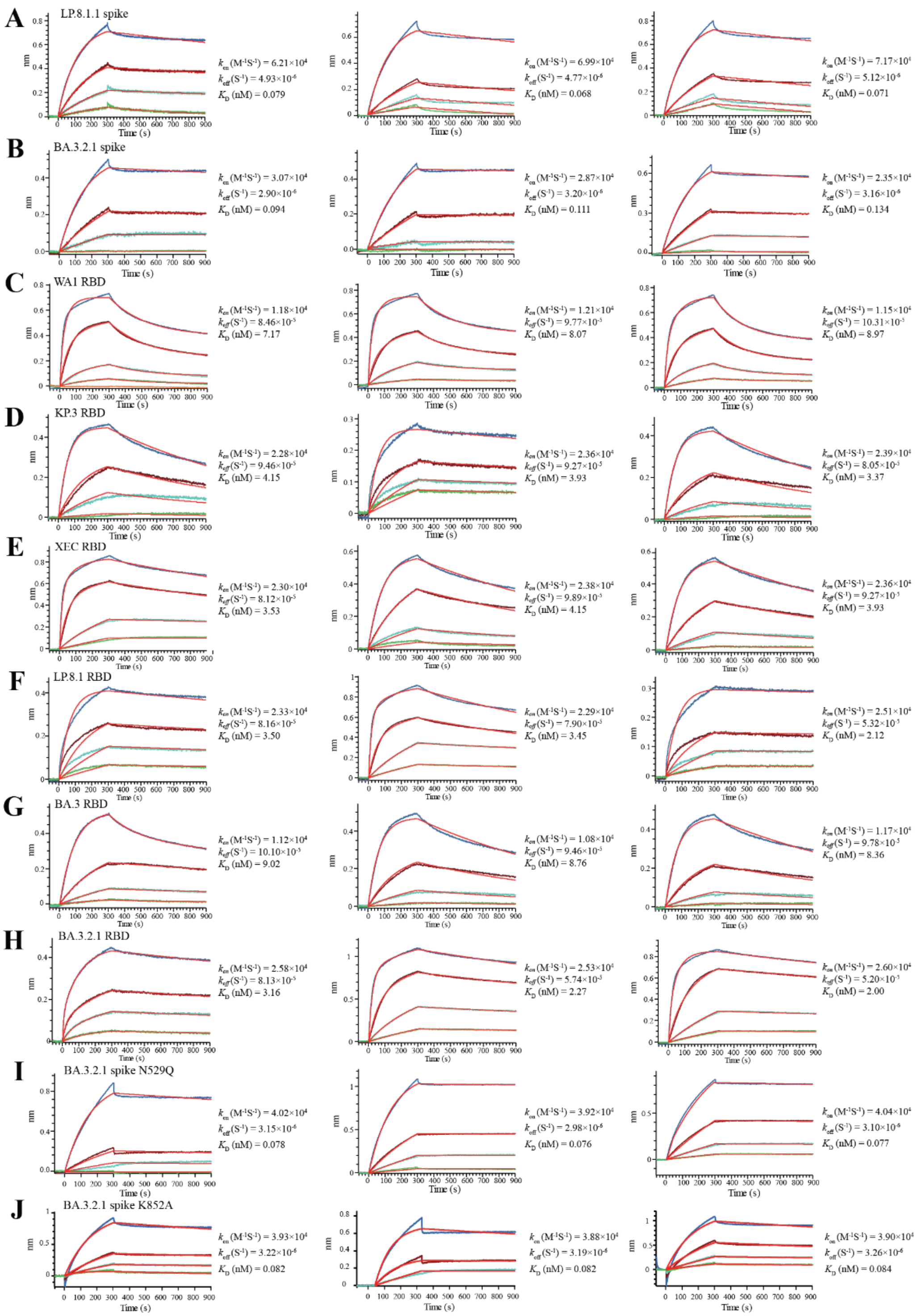

Supplemental Figure S14. BLI analysis of hACE2-RBD binding.
(A-B) Sensorgrams showing binding of trimeric spikes to hACE2: LP.8.1 spike (A) and BA.3.2.1 spike (B).
(C-H) Sensorgrams for hACE2 binding to RBDs from WA1 (C), KP.3 (D), XEC (E), LP.8.1 (F), BA.3 (G), and BA.3.2.1 (H).
(I-J) Sensorgrams showing binding of trimeric spikes to hACE2: BA.3.2.1 N529Q spike (I) and BA.3.2.1 K852A spike (J). For each panel, k_{on}, k_{off}, and K_D from each independent repeat are listed to the right. Thin

colored traces represent raw association/dissociation signals; red curves indicate the global fit to the binding model. All measurements were performed in triplicate under the same buffer and temperature conditions.

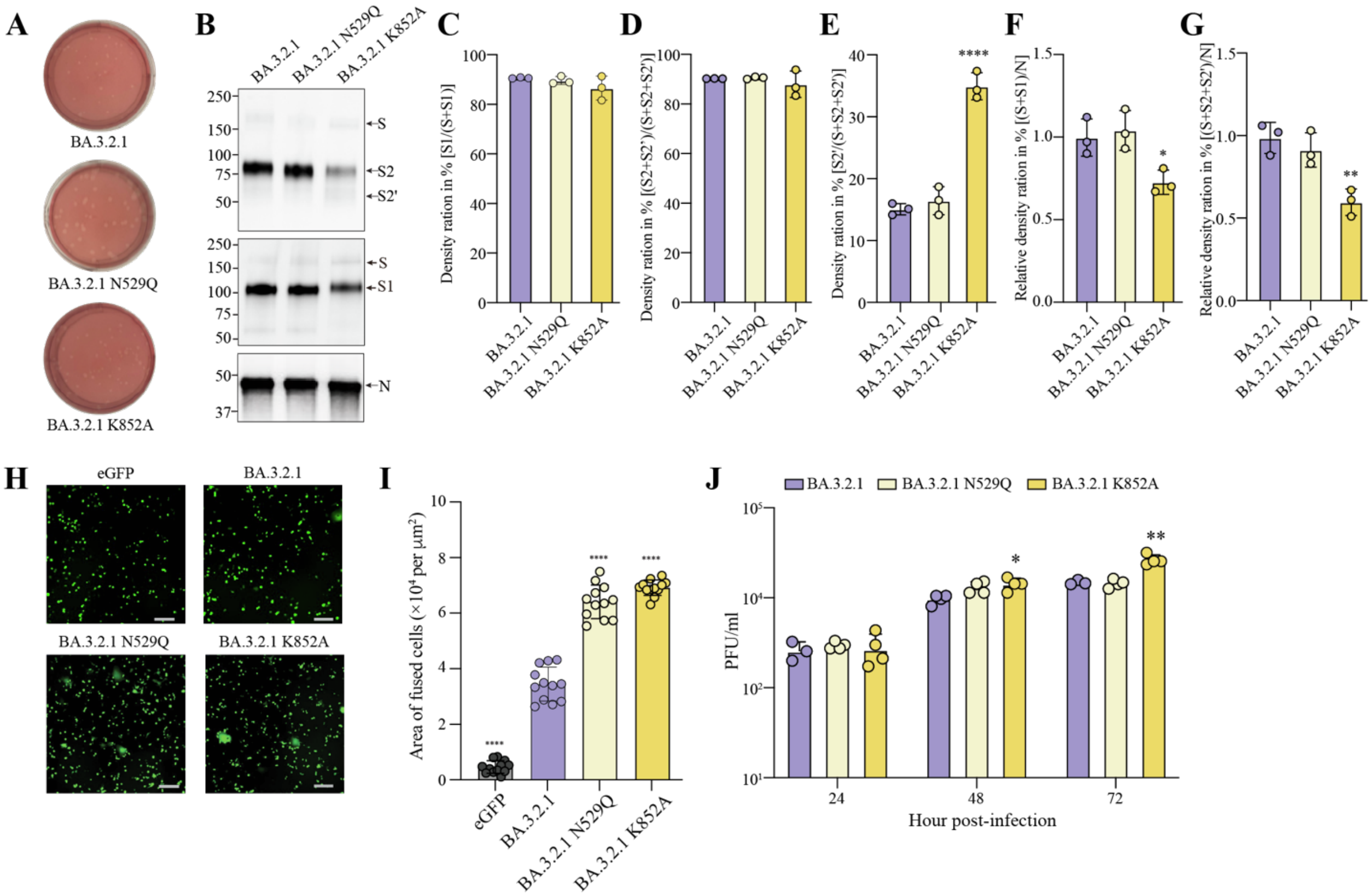

Supplemental Figure S15. Virological characterization of BA.3.2.1 spike N529Q or K852A mutant.

(A) Representative plaque morphologies of mNG SARS-CoV-2 BA.3.2.1 N529Q and K852A on Vero E6-TMPRSS2 cells.

(B) Western blot analysis of spike and nucleocapsid proteins in BA.3.2.1 N529Q and K852A virions. S, full-length spike; S1, N-terminal furin cleavage fragment of spike; S2, C-terminal furin cleavage fragment of spike; S2', N-terminal TMPRSS2 cleavage fragment of S2; N, nucleocapsid.

(C) Ratio of S1 to total spike (S+S1) in virions.

(D) Ratio of S2 and S2' to total spike (S+S2+S2') in virions.

(E) Ratio of S2' to total spike (S+S2+S2') in virions.

(F-G) Ratio of total spike to N in quantified virions using anti-S1 (F) or anti-S2 (G) antibody. Data in (C)-(G) are represented as mean ± SD. *$p < 0.05$; **$p < 0.01$; ****$p < 0.0001$, one-way ANOVA with multiple-comparison corrections.

(H) Representative images of fusion are depicted for 293T-ACE2 cells. Scale bar, 200 μm.

(I) Quantification of fusion efficiency. The mean and standard deviations from 12 independent replicates are shown. (C-G and I) Statistical significance analysis was conducted using one-way ANOVA with multiple comparison corrections. ****$p < 0.0001$.

(J) Growth kinetics of mNG WT BA.3.2.1 spike, BA.3.2.1 N529Q spike, and BA.3.2.1 K852A spike on Calu-3 cells. Data (Mean ± SD) are representative of four replicates. Statistical significance analyses were conducted using two-way ANOVA with Dunnett's multiple comparison corrections. *$p < 0.05$; **$p < 0.01$.

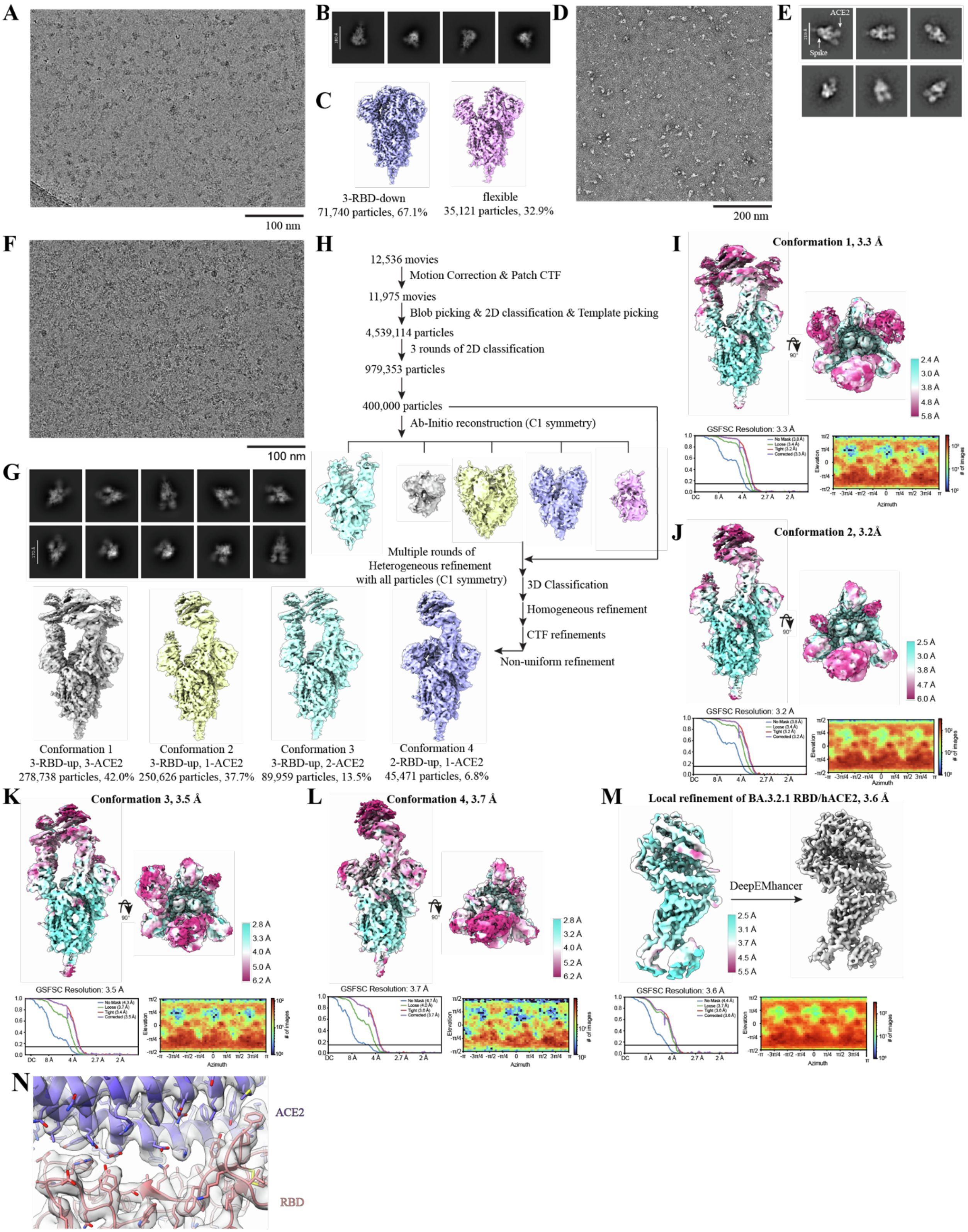

Supplemental Figure S16. Cryo-EM processing workflow for the BA.3.2.1 spike-hACE2 complex.
(A) Representative cryo-EM micrograph of the BA.3.2.1 spike/hACE2 incubated at 4°C. Scale bar, 100 nm.
(B) Representative 2D class averages of selected particles in (A).

(C) Cryo-EM reconstructions from the dataset in (A), showing only apo BA.3.2.1 spike in the closed (left) and flexible (right) conformations (no hACE2 bound).
(D) Representative negative-stain EM micrograph of the BA.3.2.1 spike/hACE2 incubated at 37°C. Scale bar, 200 nm.
(E) Representative 2D class averages of selected particles from the 37°C dataset in (D).
(F) Representative micrograph of the BA.3.2.1 spike-hACE2 complex (37°C incubation). Scale bar, 100 nm.
(G) Representative 2D class averages of selected particles.
(H) Data-processing flowchart.
(I-L) Global map quality for the non-uniform refinement maps of the BA.3.2.1 spike–hACE2 complex in conformation 1 (I), conformation 2 (J), conformation 3 (K), and conformation 4 (L), showing local-resolution distributions, FSC curves, and particle angular distributions.
(M) Local refinement of the RBD-hACE2 region for conformation 1, with corresponding local-resolution distribution, FSC curve, and angular distribution of the locally refined map.
(N) Overall cryo-EM density at the BA.3.2.1 RBD-hACE2 binding interface. Interface residues are shown as sticks with the corresponding cryo-EM density rendered for clarity.

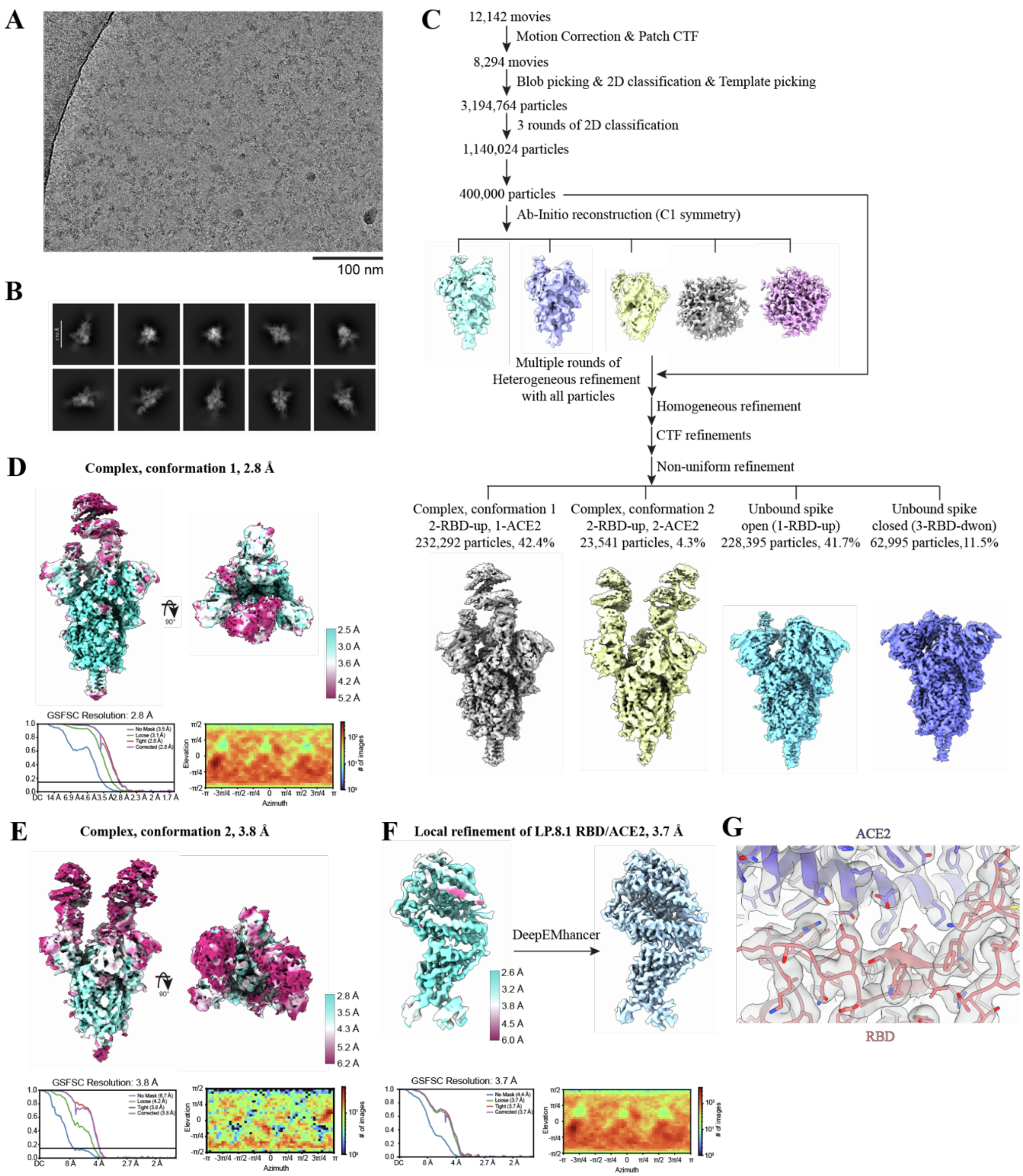

Supplemental Figure S17. Cryo-EM processing workflow for the LP.8.1 spike-hACE2 complexes.
(A) Representative micrograph of the LP.8.1 spike-hACE2 complex (ice incubation). Scale bar, 100 nm.
(B) Representative 2D class averages of selected particles.
(C) Data-processing flowchart.
(D-E) Global map quality for the LP.8.1 spike-hACE2 complex in conformation 1 (D) and conformation 2 (E), showing local-resolution distributions, FSC curves, and particle angular distributions.
(F) Local refinement of the LP.8.1 RBD-hACE2 region for conformation 1, with corresponding local-resolution distribution, FSC curve, and angular distribution for the locally refined map.
(G) Overall cryo-EM density at the LP.8.1 RBD-hACE2 binding interface. Interface residues are shown as sticks with the corresponding cryo-EM density rendered for clarity.

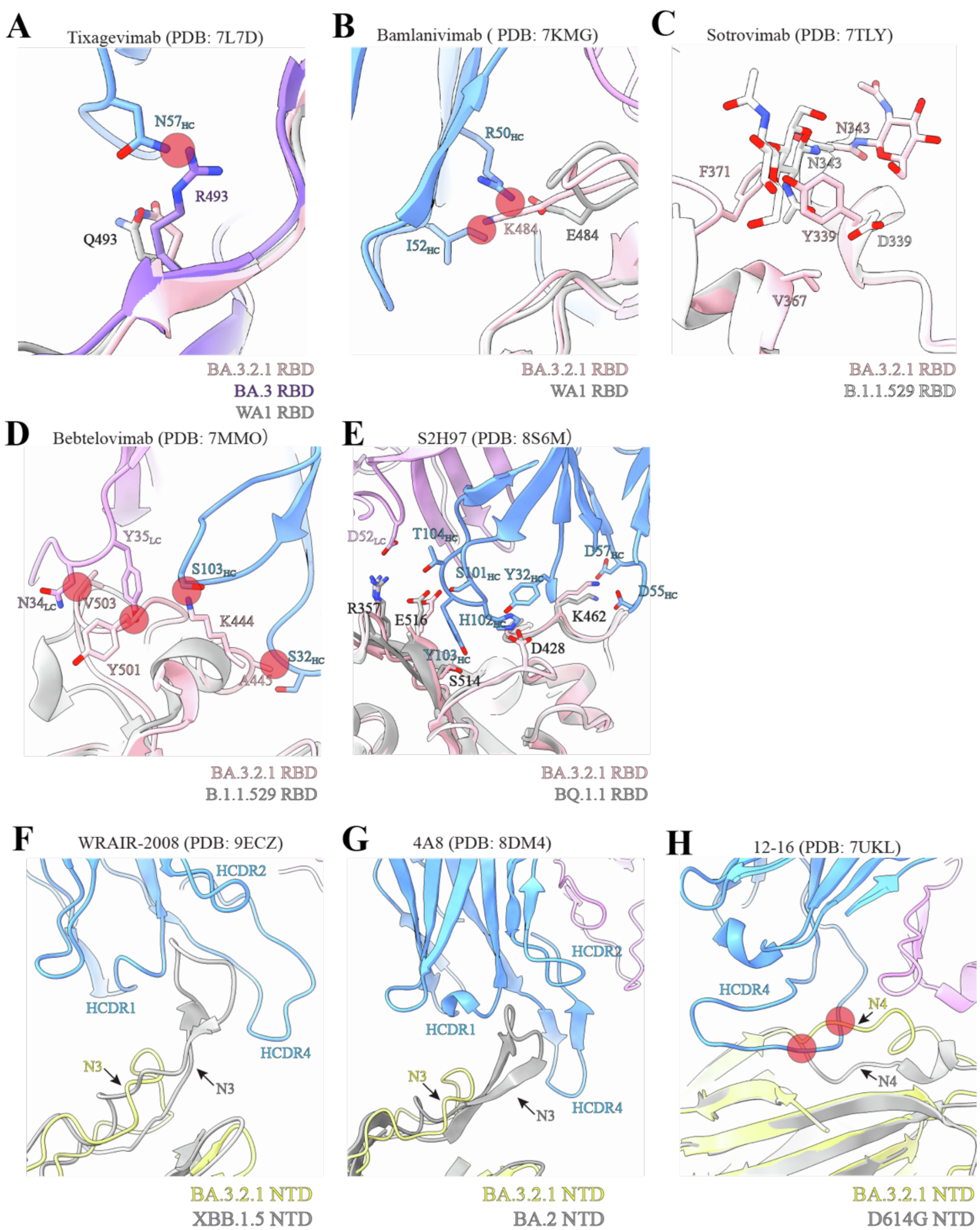

Supplemental Figure S18. Structural basis for altered antigenic properties of the BA.3.2.1 spike.
(A) Superposition of Tixagevimab/WA1 RBD with BA.3 RBD (PDB 7XB1) and BA.3.2.1 RBD.
(B) Superposition of Bamlanivimab/WA1 RBD with BA.3.2.1 RBD.
(C) Structural alignment of BA.3.2.1 RBD and B.1.1.529 RBD at the hydrophobic cluster around Y339, V367, and F371, and N343 glycan.
(D) Superposition of Bebtelovimab/B.1.1.529 RBD with BA.3.2.1 RBD.
(E) Superposition of S2H97/WA1 RBD with BA.3.2.1 RBD.
(F) Superposition of XBB.1.5 NTD/WRAIR-2008 onto the BA.3.2.1 NTD.
(G) Superposition of BA.2 NTD/4A8 onto BA.3.2.1 NTD.
(H) Superposition of D614G NTD/12-16 onto the BA.3.2.1 NTD. In all panels, RBD/NTD residues that interfere with or participate in antibody binding and the corresponding antibody residues are shown as sticks. Red circles indicate steric clashes. Antibody heavy and light chains are colored blue and plum, respectively.

Supplementary Table S1. Twenty-six human serum samples collected 21-99 days after KP.2-infection

Serum ID	Age (year)	Gender (F/M)	Race or Ethnicity	Serum collection day (post-KP.2 PCR$^+$)	Serum collection date	Doses of parental mRNA vaccine	*$FFRNT_{50}$					
							BA.3.2.1-spike	KP.3-spike	XEC-spike	LP.8.1-spike	BA.3-spike	KP.2-spike
1	23	F	Hispanic	41	6/5/24	1 dose	226	160	101	80	905	160
2	22	F	Black	58	5/29/24	2 doses	40	80	100	320	160	80
3	35	F	Black	55	6/5/24	2 doses	80	160	100	160	640	160
4	65	M	Black	59	6/9/24	3 doses	40	80	101	113	80	80
5	23	F	Black	28	7/23/24	none	10	160	107	453	80	226
6	42	F	Hispanic	24	7/29/24	2 doses	640	453	105	640	5120	1280
7	79	F	American Indian	23	8/7/24	2 doses	905	2560	98	5120	1280	3620
8	70	M	White	36	7/28/24	none	40	40	89	80	80	80
9	30	F	White	21	8/12/24	2 doses	160	160	93	160	640	160
10	76	F	Hispanic	39	7/25/24	3 doses	1810	1280	93	905	5120	1280
11	80	F	White	42	7/25/24	4 doses	226	320	87	640	640	320
12	29	F	White	66	7/1/24	none	40	160	84	113	640	320
13	82	F	White	27	8/10/24	none	20	320	84	320	113	320
14	22	F	Hispanic	43	7/24/24	none	40	80	84	80	160	40
15	75	F	Hispanic	54	7/17/24	4 doses	640	1280	91	1280	3620	1280
16	55	F	White	83	6/19/24	2 doses	113	40	85	80	640	113
17	74	F	White	27	8/15/24	2 doses	113	320	91	320	640	320
18	53	F	White	48	7/26/24	none	57	40	88	40	160	40
19	34	F	Hispanic	36	8/12/24	none	80	80	97	160	640	160
20	34	F	Hispanic	52	8/10/24	2 doses	40	80	106	80	320	80
21	29	M	Black	32	8/30/24	2 doses	80	80	111	28	640	113
22	62	M	Hispanic	99	6/28/24	4 doses	113	320	137	160	453	160
23	26	F	Hispanic	73	7/25/24	3 doses	160	320	105	640	640	320
24	28	F	Asian	41	8/28/24	4 doses	40	20	84	28	453	40
25	46	M	Hispanic	62	9/2/24	2 doses	14	113	98	80	320	226
26	28	M	Hispanic	62	9/4/24	none	57	40	113	20	640	28
Median	38.5	-	-	42.5	-	-	-	-	-	-	-	-
#GMT	-	-	-	-	-	-	91	156	104	178	484	188
†95% CI	-	-	-	-	-	-	55-153	97-251	91-101	128-292	412-741	166-340

*Individual $FFRNT_{50}$ value is the geometric mean of duplicate $FFRNT_{50}$ results.

#Geometric mean neutralizing titers (GMT).

†95% confidence interval (95% CI) for the GMT.

Supplementary Table S2. Twenty-one human serum samples collected 23-105 days after KP.3-infection

Serum ID	Age (year)	Gender (F/M)	Race or Ethnicity	Serum collection day (post-KP.3 PCR^{+})	Serum collection date	Doses of parental mRNA vaccine	*$FFRNT_{50}$					
							BA.3.2.1-spike	KP.3-spike	XEC-spike	LP.8.1-spike	BA.3-spike	KP.2-spike
1	91	F	White	23	8/8/24	5 doses	14	57	99	80	20	57
2	54	M	White	48	8/15/24	2 doses	160	320	104	320	905	453
3	67	M	White	35	8/17/24	3 doses	40	40	104	80	320	40
4	26	F	Black	33	8/22/24	none	28	160	106	226	160	160
5	34	F	Black	33	8/22/24	none	80	320	106	320	1280	640
6	68	F	Black	36	8/25/24	6 doses	80	80	102	160	640	113
7	29	F	White	34	8/28/24	2 doses	320	905	103	1280	2560	1280
8	58	F	Hispanic	79	9/7/24	3 doses	20	40	84	40	113	28
9	59	F	Black	53	9/9/24	6 doses	320	80	89	80	1280	80
10	59	M	White	50	9/9/24	5 doses	80	80	82	80	640	113
11	48	F	Hispanic	40	9/16/24	2 doses	160	160	95	320	905	320
12	62	F	White	56	9/17/24	3 doses	80	640	100	453	5120	453
13	37	M	Hispanic	54	9/20/24	3 doses	40	40	98	40	320	28
14	27	F	Black	53	10/3/24	none	40	113	110	160	320	80
15	45	F	White	39	10/3/24	2 doses	113	160	131	160	226	160
16	71	M	White	72	10/5/24	none	10	1280	135	5120	1280	1280
17	53	M	White	27	10/8/24	none	40	320	91	320	1280	160
18	82	F	White	87	10/31/24	6 doses	160	640	107	640	1280	453
19	96	F	Black	105	11/8/24	3 doses	80	320	147	453	640	453
20	48	F	White	87	12/2/24	2 doses	10	80	226	57	40	40
21	52	M	Hispanic	81	12/13/24	2 doses	226	1280	1280	1280	640	1280
Median	54	-	-	50	-	-	-	-	-	-	-	-
#GMT	-	-	-	-	-	-	63	186	108	230	508	186
†95% CI	-	-	-	-	-	-	51-108	134-264	97-150	180-365	364-764	171-326

*Individual $FFRNT_{50}$ value is the geometric mean of duplicate $FFRNT_{50}$ results.
#Geometric mean neutralizing titers (GMT).
†95% confidence interval (95% CI) for the GMT.

Supplementary Table S3. *P* values for group comparison of GMTs in Figures 1L and 1M.

Figure Panels	*P* values (two-tailed) from the Wilcoxon matched-pairs signed-rank test
Fig. 1L	KP.2- versus KP.3-, XEC-, LP.8.1-, BA.3-, BA.3.2.1-spike: 0.0387, <0.0001, 0.9099, 0.0003, 0.001; KP.3- versus XEC-, LP.8.1-, BA.3-, BA.3.2.1-spike: 0.0002, 0.2415, 0.0002, 0.0268; XEC- versus LP.8.1-, BA.3-, BA.3.2.1-spike: 0.0002, <0.0001, 0.2673; LP.8.1- versus BA.3-, BA.3.2.1-spike: 0.0021, 0.0063; BA.3- versus BA.3.2.1: <0.0001.
Fig. 1M	KP.3- versus KP.2-, XEC-, LP.8.1-, BA.3-, BA.3.2.1-spike: 0.6819, 0.0005, 0.049, 0.0006, 0.001; KP.2- versus XEC-, LP.8.1-, BA.3-, BA.3.2.1-spike: 0.0012, 0.1310, 0.0007, 0.0002; XEC- versus LP.8.1-, BA.3-, BA.3.2.1-spike: <0.0001, 0.0003, 0.1197; LP.8.1- versus BA.3-, BA.3.2.1-spike: 0.0078, 0.0002; BA.3- versus BA.3.2.1 spike: <0.0001.

Supplementary Table S4. Statistics for 3D reconstruction and model refinement for BA.3.2.1 spike and BA.3.2.1 spike-ACE2 complex.

	BA.3.2.1 spike		BA.3.2.1 spike-hACE2			
	Closed	Flexible	Class 1	Class 2	Class 3	Class 4
EMD	73393	73395	73404	73405	73408	73409
PDB	9YSJ	9YX6	-	-	-	-
**Data collection and processing**						
Microscope	Krios		Krios			
Camera	G3i		G3i			
Voltage (keV)	300		300			
Defocus range (- μm)	0.9-2.5		0.9-2.5			
Pixel size (Å)	0.832		0.832			
Electron dose ($^{-}$ Å^{-1})	39.84		39.90			
Micrographs (no.)	12,806		11,294			
**Refinement**						
Symmetry imposed	C1	C1	C1	C1	C1	C1
Particles (no.)	673,009	143,152	278,738	250,626	89,959	45,471
Map resolution (Å)	2.6	3.0	3.3	3.2	3.5	3.7
Initial model (PDB code)	7XIY	7XIY	-	-	-	-
**Model composition**						
Chains	3	3	-	-	-	-
Atoms	24,392	21,789	-	-	-	-
Residues (Protein)	3,021	2,706	-	-	-	-
Ligands (NAG)	47	41	-	-	-	-
**R.m.s. deviations**						
Bond lengths (Å)	0.005	0.003	-	-	-	-
Bond angles (°)	0.569	0.573	-	-	-	-
**Model statistics**						
Clash score	6.93	8.84	-	-	-	-
MolProbity score	1.66	1.73	-	-	-	-
Rotamer outliers (%)	0.75	0.71	-	-	-	-
**Ramachandran plot**						
Outliers (%)	0.00	0.00	-	-	-	-
Allowed (%)	4.08	3.73	-	-	-	-
Favored (%)	95.92	96.27	-	-	-	-

Supplementary Table S5. Statistics for 3D reconstruction and model refinement for LP.8.1 spike and LP.8.1 spike-ACE2 complex.

	LP.8.1 spike			LP.8.1 spike-ACE2			
	Closed	Open	Flexible	Complex		Apo Spike	
				Class 1	Class 2	Open	Closed
EMD	73394	73535	73396	73427	73428	73430	73439
PDB	9YSK	9YW0	-	-	-	-	-
**Data collection and processing**							
Microscope	Krios			Krios			
Camera	G3i			G3i			
Voltage (keV)	300			300			
Defocus range (- µm)	0.9-2.5			0.9-2.5			
Pixel size (Å)	0.832			0.832			
Electron dose ($^{-}$ Å^{-1})	39.9			39.8			
Micrographs (no.)	15,414			8,294			
**Refinement**							
Symmetry imposed	C1	C1	C1	C1	C1	C1	C1
Particles (no.)	948,866	935,991	731,058	232,292	23,541	228,395	62,995
Map resolution (Å)	2.5	2.5	2.6	2.8	3.8	2.8	3.2
Initial model (PDB code)	9ELI	9ELL	-	-	-	-	-
**Model composition**							
Chains	3	3	-	-	-	-	-
Atoms	24,010	24,827	-	-	-	-	-
Residues (Protein)	2,989	3,083	-	-	-	-	-
Ligands	41	46	-	-	-	-	-
**R.m.s. deviations**							
Bond lengths (Å)	0.003	0.002	-	-	-	-	-
Bond angles (°)	0.517	0.528	-	-	-	-	-
**Model statistics**							
Clash score	6.62	6.92	-	-	-	-	-
MolProbity score	1.53	1.67	-	-	-	-	-
Rotamer outliers (%)	0.76	0.96	-	-	-	-	-
**Ramachandran plot**							
Outliers (%)	0.00	0.00	-	-	-	-	-
Allowed (%)	2.91	4.19	-	-	-	-	-
Favored (%)	97.09	95.81	-	-	-	-	-

Supplementary Table S6. Intra-protomer vector descriptions

Distance	Centroids	Description
d_1	NTD, NTD′	Term describing the distance between NTD and NTD′ within the same protomer
d_2	NTD′, SD2	Term describing the distance between NTD′ and SD2 within the same protomer
d_3	SD2, SD1	Term describing the distance between SD2 and SD1 within the same protomer
d_4	SD2, CD	Term describing the distance between SD2 and CD within the same protomer
d_5	SD1, RBD	Term describing the distance between SD1 and RBD within the same protomer
d_6	CD, S2s	Term describing the distance between CD and S2s within the same protomer
d_7	NTDa, NTD	Term describing the distance between NTDa and NTD within the same protomer
d_8	RBD, RBDa	Term describing the distance between RBD and RBDa within the same protomer
d_9	CD, SD2	Term describing the distance between CD and SD2 within the same protomer
d_{10}	SD1, SD2	Term describing the distance between SD1 and SD2 within the same protomer
Angle		
θ_1	SD2, SD1, RBD	Term describing the angular motion of SD2 relative to RBD about SD1
θ_2	NTDa, NTD, NTD′	Term describing the angular motion of NTDa relative to NTD about NTD′
θ_3	NTD′, SD2, SD1	Term describing the angular motion of NTD′ relative to SD2 about SD1
θ_4	NTD, NTD′, SD2	Term describing the angular motion of NTD relative to NTD′ about SD2
θ_5	SD1, RBD, RBDa	Term describing the angular motion of SD1 relative to RBD about RBDa
θ_6	NTD′, SD2, CD	Term describing the angular motion of NTD′ relative to SD2 about CD
θ_7	SD2, CD, S2s	Term describing the angular motion of SD2 relative to CD about S2s
θ_8	CD, SD2, SD1	Term describing the angular motion of CD relative to SD2 about SD1
Dihedral		
Φ_1	SD2, SD1, RBD, RBDa	Dihedral describing rotation of SD2 and RBDa about SD1 to RBD axis
Φ_2	NTDa, NTD, NTD′, SD2	Dihedral describing rotation of NTDa and SD2 about NTD to NTD′ axis
Φ_3	NTD′, SD2, SD1, RBD	Dihedral describing rotation of NTD′ and RBD about SD2 to SD1 axis
Φ_4	NTD′, SD2, CD, S2s	Dihedral describing rotation of NTD′ and S2s about SD2 to CD axis
Φ_5	NTD, NTD′, SD2, SD1	Dihedral describing rotation of NTD and SD1 about SD2 to NTD′ axis
Φ_6	NTD, NTD′, SD2, CD	Dihedral describing rotation of NTD and CD about SD2 to NTD′ axis
Φ_7	SD1, SD2, CD, S2s	Dihedral describing rotation of SD1 and S2s about SD2 to CD axis

Supplementary Table S7. Inter-protomer vector descriptions

Distance	Centroids	Description
d_1'	RBD_A, RBD_C	Term describing the distance between RBD from protomer A and C
d_2'	RBD_C, RBD_B	Term describing the distance between RBD from protomer B and C
d_3'	RBD_A, RBD_B	Term describing the distance between RBD from protomer A and B
d_4'	RBD_A, NTD_C	Term describing the distance between RBD from protomer A and NTD from protomer C
d_5'	RBD_C, NTD_B	Term describing the distance between RBD from protomer C and NTD from protomer B
d_6'	RBD_B, NTD_A	Term describing the distance between RBD from protomer B and NTD from protomer A
d_7'	$SD1_A$, NTD'_C	Term describing the distance between SD1 from protomer A and NTD′ from protomer C
d_8'	$SD1_C$, NTD'_B	Term describing the distance between SD1 from protomer C and NTD′ from protomer B
d_9'	$SD1_B$, NTD'_A	Term describing the distance between SD1 from protomer B and NTD′ from protomer A
Angle		
θ_1'	RBD_A, RBD_C, RBD_B	Term describing the angular motion of RBD_A relative to RBD_B about RBD_C
θ_2'	RBD_C, RBD_B, RBD_A	Term describing the angular motion of RBD_C relative to RBD_A about RBD_B
θ_3'	RBD_B, RBD_A, RBD_C	Term describing the angular motion of RBD_B relative to RBD_C about RBD_A
Dihedral		
Φ_1'	$RBDa_A$, RBD_A, RBD_C, $RBDa_C$	Dihedral describing rotation of $RBDa_A$ and $RBDa_C$ about RBD_A to RBD_C axis
Φ_2'	$RBDa_C$, RBD_C, RBD_B, $RBDa_B$	Dihedral describing rotation of $RBDa_C$ and $RBDa_B$ about RBD_C to RBD_B axis
Φ_3'	$RBDa_B$, RBD_B, RBD_A, $RBDa_A$	Dihedral describing rotation of $RBDa_B$ and $RBDa_A$ about RBD_B to RBD_A axis
Φ_4'	$SD1a_A$, $SD1_A$, NTD'_C, $NTD'a_C$	Dihedral describing rotation of $SD1a_A$ and $NTD'a_C$ about $SD1_A$ to NTD'_C axis
Φ_5'	$SD1a_C$, $SD1_C$, NTD'_B, $NTD'a_B$	Dihedral describing rotation of $SD1a_C$ and $NTD'a_B$ about $SD1_C$ to NTD'_B axis
Φ_6'	$SD1a_B$, $SD1_B$, NTD'_A, $NTD'a_A$	Dihedral describing rotation of $SD1a_B$ and $NTD'a_A$ about $SD1_B$ to NTD'_A axis
Φ_7'	$SD2a_A$, $SD1_A$, NTD'_C, $SD2a_C$	Dihedral describing rotation of $SD2a_A$ and $SD2a_C$ about $SD1_A$ to NTD'_C axis
Φ_8'	$SD2a_C$, $SD1_C$, NTD'_B, $SD2a_B$	Dihedral describing rotation of $SD2a_C$ and $SD2a_B$ about $SD1_C$ to NTD'_B axis
Φ_9'	$SD2a_B$, $SD1_B$, NTD'_A, $SD2a_A$	Dihedral describing rotation of $SD2a_B$ and $SD2a_A$ about $SD1_B$ to NTD'_A axis

Supplementary Table S8. Statistics for 3D reconstruction and refinement for BA.3.2.1 spike local refinement

	RBD_C /RBD_A/NTD_B Closed spike	RBD_A/NTD_C Closed spike	RBD_B/NTD_A Closed spike	NTD_B Closed spike	RBD/hACE2 Conformation 1
EMD	73599	73597	73619	73795	73426
PDB	9YX8	9YX7	9YXX	9Z3Y	9YSR
**Refinement**					
Symmetry imposed	C1	C1	C1	C1	C1
Particles (no.)	673,009	673,009	673,009	673,009	278,738
Map resolution (Å)	2.9	2.9	3.0	3.0	3.6
**Model composition**					
Chains	3	2	2	1	2
Atoms	4,848	3,609	3,737	1,936	6,273
Residues (Protein)	585	434	453	255	757
Ligands (NAG)	6	6	6	7	6
**R.m.s. deviations**					
Bond lengths (Å)	0.004	0.003	0.004	0.003	0.003
Bond angles (°)	0.622	0.613	0.623	0.633	0.654
**Model statistics**					
Clash score	6.65	4.23	4.90	5.77	9.14
MolProbity score	1.63	1.53	1.69	1.69	1.71
Rotamer outliers (%)	0.76	0.51	0.74	0.49	0.60
**Ramachandran plot**					
Outliers (%)	0.00	0.00	0.00	0.00	0.00
Allowed (%)	3.85	4.74	6.58	5.48	3.47
Favored (%)	96.15	95.26	93.42	94.52	96.53

Supplementary Table S9. Statistics for 3D reconstruction and model refinement for LP.8.1 local refinement

	RBD_A/NTD_C Closed spike	RBD_B/NTD_A Closed spike	RBD_C/NTD_B Closed spike	RBD/hACE2 complex Conformation 1
EMD	73441	73443	73444	73429
PDB	9YT4	9YT6	9YT7	9YSS
**Refinement**				
Symmetry imposed	C1	C1	C1	C1
Particles (no.)	948,866	948,866	948,866	232,292
Map resolution (Å)	2.8	2.8	2.8	3.7
**Composition**				
Chains	2	2	2	2
Atoms	4,229	4,192	4,205	6,312
Residues (Protein)	510	508	510	761
Ligands (NAG)	11	9	9	8
**R.m.s. deviations**				
Bond lengths (Å)	0.004	0.003	0.004	0.004
Bond angles (°)	0.654	0.582	0.690	0.717
**Model statistics**				
Clash score	3.97	5.83	6.05	8.60
MolProbity score	1.36	1.50	1.45	1.69
Rotamer outliers (%)	0.22	0.22	0.22	0.75
**Ramachandran plot**				
Outliers (%)	0.00	0.00	0.00	0.00
Allowed (%)	3.02	3.04	2.62	3.44
Favored (%)	97.18	96.96	97.38	96.56

Supplementary Table S10. Statistics for 3D reconstruction and model refinement for BA.3.2.1 N529Q or K852A spikes

	BA.3.2.1 N529Q			BA.3.2.1 K852A		
	Closed	Open	Flexible	Closed	Open	Flexible
EMD	76493	76850	76706	76501	76839	76694
PDB	12JT	-	-	12JZ	-	-
**Data collection and processing**						
Microscope	Krios			Krios		
Camera	G4			G4		
Voltage (keV)	300			300		
Defocus range (- µm)	0.9-2.5			0.9-2.5		
Pixel size (Å)	0.832			0.832		
Electron dose ($^{-}$ Å^{-1})	41.1			41.1		
Micrographs (no.)	10,761			10,007		
**Refinement**						
Symmetry imposed	C1	C1	C1	C1	C1	C1
Particles (no.)	516,314	192,202	155,678	656,342	128,756	114,972
Map resolution (Å)	2.4	2.6	2.8	2.4	2.7	2.9
Initial model (PDB code)	9YSJ	-	-	9YSJ	-	-
**Model composition**						
Chains	3	-	-	3	-	-
Atoms	24,611	-	-	23,958	-	-
Residues (Protein)	3,053	-	-	2,966	-	-
Ligands	44	-	-	45	-	-
**R.m.s. deviations**						
Bond lengths (Å)	0.004	-	-	0.003	-	-
Bond angles (°)	0.535	-	-	0.594	-	-
**Model statistics**						
Clash score	2.85	-	-	3.90	-	-
MolProbity score	1.28	-	-	1.26	-	-
Rotamer outliers (%)	0.56	-	-	0.69	-	-
**Ramachandran plot**						
Outliers (%)	0.00	-	-	0.00	-	-
Allowed (%)	3.28	-	-	2.42	-	-
Favored (%)	96.72	-	-	97.58	-	-

Supplementary Table S11. Statistics for 3D reconstruction and model refinement for BA.3.2.1 N529Q and K852A local refinement

	RBD_C/NTD_B Closed N529Q spike	$RBD_C/RBD_A/NTD_B$ Closed K852A spike
EMD	76936	76713
PDB	13BD	12RP
**Refinement**		
Symmetry imposed	C1	C1
Particles (no.)	516,314	656,342
Map resolution (Å)	2.7	2.8
**Composition**		
Chains	2	2
Atoms	3,825	4,903
Residues (Protein)	461	595
Ligands (NAG)	8	4
**R.m.s. deviations**		
Bond lengths (Å)	0.004	0.004
Bond angles (°)	0.647	0.554
**Model statistics**		
Clash score	4.13	3.65
MolProbity score	1.54	1.47
Rotamer outliers (%)	0.97	0.94
**Ramachandran plot**		
Outliers (%)	0.00	0.00
Allowed (%)	4.94	4.52
Favored (%)	95.06	95.48

Supplementary Table S12. List of primers used in this study.

Primer Name	Sequence (5'-3')	Notes
CoV-21115V	CATTTGTGGGTTTATACAACAAAAG	For RT-PCR
CoV-YH5	AGCATCCTTGATTTCACC	For RT-PCR
K529N-F	CCACTTATGGTGTTGGTTACCAACC	For competition assay of BA.3 and BA.3.2.1
K529N-R	GCAATGTCTCTGCCAAATTGTTGG	For competition assay of BA.3 and BA.3.2.1
A852K-F	CAACAAAGTGACACTTGCAGATGC	For competition assay of BA.3.2.1 and LP.8.1
A852K-R	GTGATTGTACCCGCTAACAGTGC	For competition assay of BA.3.2.1 and LP.8.1
K1086-F	AAGGGCTATCATCTTATGTCCTTCC	For competition assay of LP.8.1 and XEC
K1086-R	CAAATGTGTTGTCTGTAGTAATGATTTGTGG	For competition assay of LP.8.1 and XEC
F59S-F	CTTTCACACGTGGTGTTTATTACCC	For competition assay of KP.3 and XEC
F59S-R	CTTCTCAGTGGAAGCAAAATAAACACC	For competition assay of KP.3 and XEC
N529Q-F	GTGGACCTAAACAGTCTACTAATTTGG	For generation of BA.3.2.1 spike mutation N529Q
N529Q-R	AGTAGACTGTTTAGGTCCACAAACAGTTG	For generation of BA.3.2.1 spike mutation N529Q
K852A-F	CTCATTTGTGCTCAAAAGTTTAACGGCC	For generation of BA.3.2.1 spike mutation K852A
K852A-R	AACTTTTGAGCACAAATGAGGTCTCTAGC	For generation of BA.3.2.1 spike mutation K852A
2019-nCoV_N2-F	TTACAAACATTGGCCGCAAA	For RT-qPCR
2019-nCoV_N2-R	GCGCGACATTCCGAAGAA	For RT-qPCR
